# Multiplex generation and single-cell analysis of structural variants in mammalian genomes

**DOI:** 10.1126/science.ado5978

**Published:** 2025-01-31

**Authors:** Sudarshan Pinglay, Jean-Benoît Lalanne, Riza M. Daza, Sanjay Kottapalli, Faaiz Quaisar, Jonas Koeppel, Riddhiman K. Garge, Xiaoyi Li, David S. Lee, Jay Shendure

**Affiliations:** 1 Department of Genome Sciences, University of Washington, Seattle, WA, USA.; 2 Brotman Baty Institute for Precision Medicine, Seattle, WA, USA.; 3 Seattle Hub for Synthetic Biology, Seattle, WA, USA.; 4 Wellcome Sanger Institute, Hinxton, UK.; 5 Institute for Protein Design, University of Washington, Seattle, WA, USA.; 6 Allen Discovery Center for Cell Lineage Tracing, Seattle, WA, USA.; 7 Howard Hughes Medical Institute, Seattle, WA, USA.

## Abstract

Studying the functional consequences of structural variants (SVs) in mammalian genomes is challenging because (i) SVs arise much less commonly than single-nucleotide variants or small indels and (ii) methods to generate, map, and characterize SVs in model systems are underdeveloped. To address these challenges, we developed Genome-Shuffle-seq, a method that enables the multiplex generation and mapping of thousands of SVs (deletions, inversions, translocations, and extrachromosomal circles) throughout mammalian genomes. We also demonstrate the co-capture of SV identity with single-cell transcriptomes, facilitating the measurement of SV impact on gene expression. We anticipate that Genome-Shuffle-seq will be broadly useful for the systematic exploration of the functional consequences of SVs on gene expression, the chromatin landscape, and three-dimensional nuclear architecture, while also initiating a path toward a minimal mammalian genome.

## Abstract (summary)

**INTRODUCTION:**

The structural variants (SVs) present in mammalian genomes include deletions, insertions, inversions, duplications, translocations, extrachromosomal DNA circles (ecDNAs), and complex rearrangements. In an individual human genome, SVs collectively affect more nucleotides than any other class of genetic variation and have been associated with myriad rare and common diseases as well as normal phenotypic variation. However, it has been very challenging to study the functional consequences of SVs at scale, largely because methods to generate, map, and characterize SVs in model systems (e.g., mammalian cell lines) are grossly underdeveloped.

**RATIONALE:**

To address this challenge, we developed Genome-Shuffle-seq, a method designed for the multiplex generation, mapping, and characterization of several major SV classes (deletions, inversions, translocations, and ecDNAs) throughout a mammalian genome. Genome-Shuffle-seq leverages barcoded, genomically integrated “shuffle cassettes” whose design facilitates: (i) bp resolution, genome-wide mapping of the coordinates of shuffle cassette integrations, through T7 in vitro transcription; (ii) site-specific recombination of shuffle cassettes with one another, mediated by recombinases such as Cre or Bxb1; (iii) conversion of barcodes from parental to novel pairings upon recombination, with each novel pair reflecting the breakpoints and class of event that generated it; and (iv) a co-assay of mRNAs and barcode pairings by coupling of T7 in situ transcription and single-cell RNA-seq. These design attributes are intended not only to generate a diversity of SVs in a single experiment, but also to facilitate their efficient and cost-effective mapping and quantification, i.e., forgoing the need for cell line cloning or whole-genome sequencing.

**RESULTS:**

In this proof of concept, we applied Genome-Shuffle-seq to mouse embryonic stem cells and human cancer cells, generating and mapping hundreds to thousands of SVs per experiment. We find that cells in which SVs are generated through Cre-mediated recombination of symmetric loxP sites are rapidly depleted, potentially due to toxicity of Cre and/or of the SVs themselves. By contrast, cells in which SVs are generated by Bxb1-mediated recombination at asymmetric attB/P sites are stable to propagation. This stability enabled us to investigate selection pressures acting on different classes of Bxb1-induced SVs, as well as to begin characterizing their functional consequences. First, we find that cells bearing large deletions, but not inversions, are preferentially lost from a proliferating cellular population and that this is partly attributable to intolerance for centromere loss. Second, we observe that although balanced translocations are tolerated in vitro, unbalanced translocations, particularly those that are acentric, are rapidly depleted. Finally, by co-assaying the transcriptome and shuffle cassette barcode pairings in a bottlenecked population of genome-shuffled cells, we demonstrate that we can measure the consequences of specific, induced SVs on gene expression.

**CONCLUSION:**

Genome-Shuffle-seq is a straightforward method that unlocks the possibility of pooled cellular screens to quantify the functional consequences of SVs spanning the entire human genome on fitness, gene expression, chromatin state, and three-dimensional nuclear architecture. Such data may (i) facilitate the interpretation and mechanistic investigation of SVs associated with human phenotypes; (ii) advance our understanding of the functional architecture of mammalian genomes; and (iii) inform efforts to design and construct a minimal human genome.

## Graphical Abstract

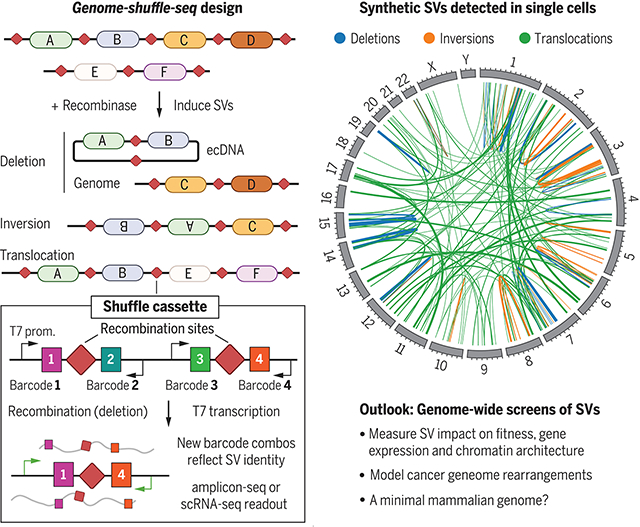

**Genome-shuffle-seq enables the generation and characterization of thousands of structural variants (SVs) in mammalian genomes, even at single-cell resolution**. The method uses “shuffle-cassettes” containing site-specific recombinase sites, unique DNA barcodes, and phage T7 promoters. Recombination between shuffle cassettes results in novel barcode combinations that reflect SV identity, which is detectable in bulk through polymerase chain reaction–amplicon sequencing or with single-cell RNA-seq after T7 transcription. BioRender.com was used to create some schematics in this figure.

Major classes of human genetic variation include single-nucleotide variants (SNVs), indels and structural variants (SVs) [e.g., deletions, insertions, inversions, and duplications >50 base pairs (bps), as well as chromosomal translocations] (1, 2). For both human and experimental genetics, SVs are much more challenging to study than SNVs or indels.

For human genetics, de novo SVs are greater than 100 times less frequent than de novo SNVs per generation (3). The lower rate of de novo occurrence in SVs, together with a greater likelihood of fitness effects (as SVs disrupt orders-of-magnitude more bps per event), contribute to their numerical paucity among standing genetic variants in human populations (3–7). SVs are less likely to recur, and even when they affect a shared region they may have different breakpoints. Whereas SNVs or indels typically disrupt one gene or regulatory element, SVs often affect multiple genes or elements, limiting resolution to assign causality for an associated phenotype. Far fewer SVs than SNVs or indels reach the allele frequencies necessary for well-powered genome-wide association studies. Althosugh every possible SNV compatible with life is likely present in a living human (8), this is certainly not the case for every possible SV.

For experimental genetics, numerous strategies exist to introduce SNVs or indels into model systems for functional analysis, including chemical mutagenesis, base editing (9), and saturation mutagenesis (10, 11). The resulting data are useful for functionally annotating genes (12), characterizing the distribution of effect sizes of regulatory or coding variants (10, 11, 13), adjudicating clinical variants of uncertain significance (11), optimizing immuno-therapies (14), etc.

However, SVs are again at a clear disadvantage, as methods to experimentally generate, map, and quantify SVs in model systems remain immature. For example, site-specific recombinase (SSR) recognition sites can be introduced to specific genomic locations, such that their recombination results in a specific SV or even an extrachromosomal circle (ecDNA) species of interest (15–17). However, this is labor intensive and yields only one or a few SVs to study. Alternatively, CRISPR/Cas9-mediated double-stranded breaks (DSB) can induce larger numbers of SVs, potentially even genome-wide (18–20). But this approach is challenged by inefficiency, imprecision, DSB toxicity, and an inability to efficiently map which cells harbor which (if any) induced SVs. Sauvageau and colleagues used retrovirally integrated SSR recognition sites to generate a panel of mouse embryonic stem cells (mESC) clones bearing nested deletions covering ~25% of the mouse genome (21, 22). However, this method lacked a means to efficiently map SSR recognition site locations and post induction SVs. In yeast, chromosome-specific or genome-wide “scrambles” were achieved by first building synthetic chromosomes bearing many SSR recognition sites (23–25), but for mammalian genomes, whole-chromosome or genome synthesis remains impractical. Finally, all current approaches aiming for multiplex SV generation rely on inefficient and/or expensive methods for the verification and quantification of SVs (e.g., single-cell cloning, whole-genome sequencing, karyotyping).

Consequent to these disadvantages, there remain numerous unanswered “structure-function” questions about the human genome that relate to its properties at the scale of SVs rather than SNVs or indels. Genes, exons, and cis-regulatory elements are scattered over vast distances, but our understanding of the functional implications of their distances, orders, and orientations remains shallow. One-quarter of the human genome is composed of gene deserts, with conservation patterns suggesting that at least some elements therein are functional (26). However, deletion of even megabase-sized deserts can yield viable mice with no discernable phenotype (27, 28). Other nongenic SVs clearly cause Mendelian disorders, contribute to complex disease risk, or underlie evolutionary adaptations (29), but there are few cases in which we understand precisely how. Although gene content may be relatively stable, most mammalian genomes differ from the human genome by >1 billion bps in turnover (gain and loss) of noncoding regions, largely through SVs (30, 31). Beyond the germline, somatic SVs play critical but poorly understood roles in the initiation and progression of human cancer, including cancer-specific forms of SV like chromothripsis and ecDNAs (32, 33).

Motivated by these gaps, we developed Genome-Shuffle-seq, a straightforward method for multiplex generation of large-scale SVs throughout a mammalian genome (Fig. 1). Genome-Shuffle-seq enables the facile mapping and genotyping of induced SVs breakpoints at bp resolution. As a proof of concept, we induce, quantify, and map the breakpoints of thousands of SVs (deletions, inversions, chromosomal translocations, ecDNAs) in two mammalian cell lines. We also demonstrate co-capture of the identities of induced SVs through single-cell RNA-seq (scRNA-seq), laying the foundation for pooled cellular screens of thousands of mammalian SVs.

## Design of Genome-Shuffle-seq

**Fig. 1.**
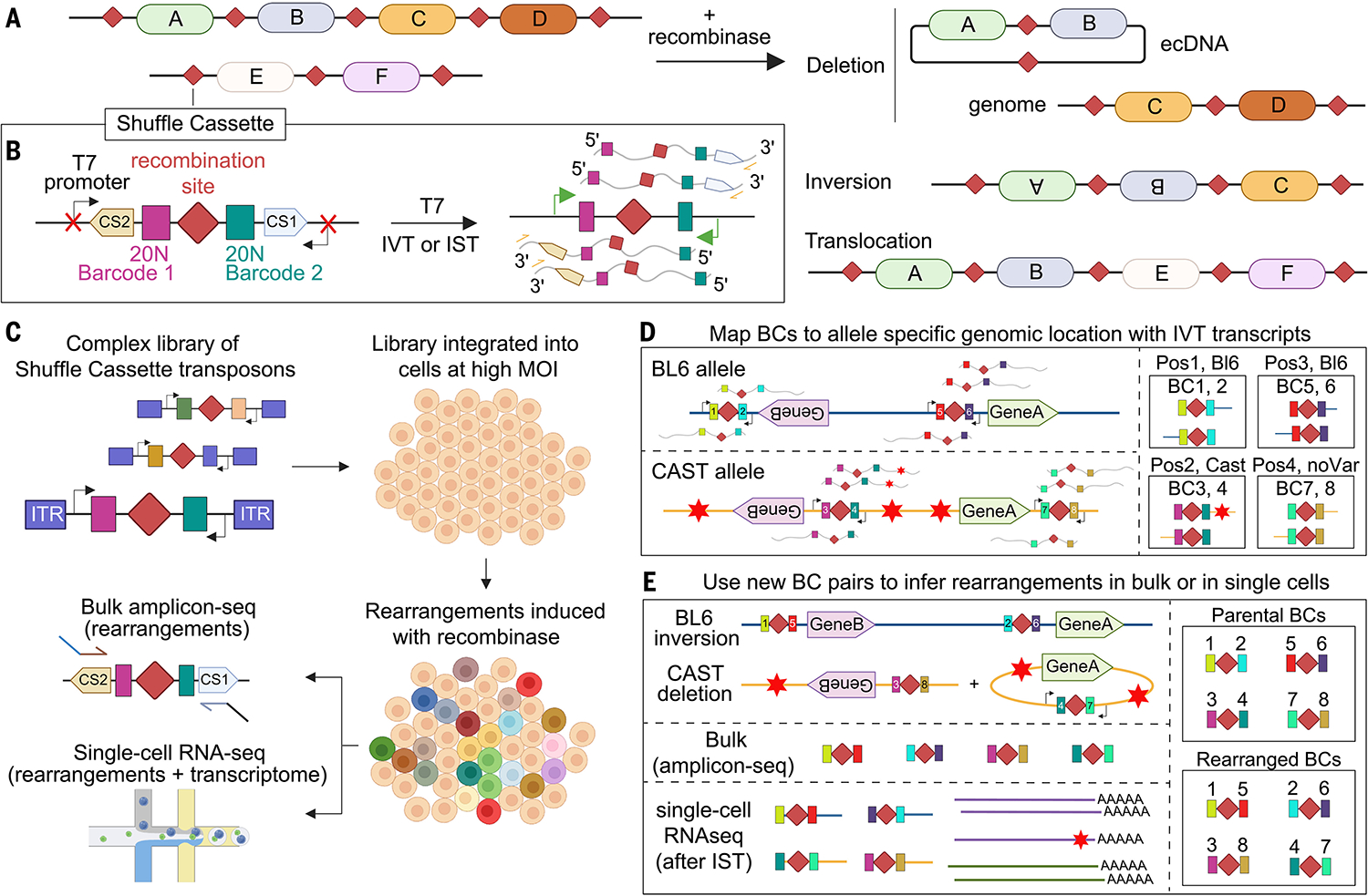
Schematic of Genome-Shuffle-seq for the pooled construction and efficient characterization of rearranged mammalian genomes at single-cell resolution. (**A**) Arrays of integrated recombinase sites can be recombined by SSRs to yield three classes of SVs. (**B**) Schematic of the shuffle cassette, which contains a recombinase site flanked by unique 20N barcodes, capture sequences for scRNA-seq (CS1, CS2), and phage polymerase promoters that are inert in live mammalian cells but activated upon in vitro (IVT) or in situ (IST) transcription with T7 polymerase. (**C**) Workflow of a Genome-Shuffle-seq experiment. (**D**) Shuffle cassette insertion sites can be mapped by sequencing T7-derived transcripts from IVT or IST and associating a pair of unique barcodes (numbered 1 to 8 in the schematic) to a genomic location. Allele-specific integration sites can be determined in hybrid cells such as BL6XCAST mESCs. Red stars indicate variants between the BL6 and Castaneus haplotypes in genomic DNA flanking an example integration site. (**E**) Induced SVs can be inferred by novel barcode combinations that are only observed in amplicons or scRNA-seq data from cells that have been exposed to recombinase. As the genomic coordinates of the parental barcodes are known from IVT-based mapping of their locations in the parental cell population, the identity of the barcodes making up each novel combination is sufficient to infer both the class (deletion, inversion, translocation) as well as the precise genomic coordinates involved in each induced SV.

Genome-Shuffle-seq is based on the integration of “shuffle cassettes” to a mammalian genome (Fig. 1, A to C). Shuffle cassettes are designed to facilitate (i) mapping genomic coordinates of integration sites, (ii) generation of SVs through SSR between pairs of shuffle cassettes, and (iii) efficient recovery of genotype information. Our initial shuffle cassette design was 176 bps with four key features (Fig. 1B): (i) A loxPsym site, in which Cre-mediated recombination between pairs of this symmetric variant of the canonical loxP site is expected to yield deletions and inversions at roughly equal frequencies (23, 34), as well as translocations. (ii) Flanking loxPsym, a pair of degenerate 20 nucleotide (nt) barcodes, to uniquely tag each shuffle cassette integration or its recombined derivatives. (iii) Flanking the barcodes is a pair of primer binding sites (PBSs) (35) (fig. S1A) and (iv) flanking the PBSs is a pair of convergently oriented phage T7 RNA polymerase promoters, inert in living mammalian cells but activatable with T7 polymerase for in vitro transcription (IVT) on genomic DNA (gDNA) or in situ transcription (IST) on fixed cells (36, 37) (fig. S1B).

Following shuffle cassette integration [e.g., randomly through transposition at a high multiplicity of infection (MOI) or, alternatively, in a targeted fashion], their locations are precisely mapped by sequencing T7 IVT-derived transcripts spanning both cassette-specific barcodes and flanking genomic sequence, with a straight forward protocol that we recently described (37) (Fig. 1D and fig. S1B). Starting from a parental cell population wherein each cell contains a distinct repertoire of integrated, mapped shuffle cassettes, Cre recombinase induces SVs by driving recombination between pairs of SSR recognition sites. Because these recombination events shuffle which 20-nt barcodes are linked, specific SVs are detected and quantified simply by sequencing shuffle cassette-derived polymerase chain reaction (PCR) amplicons, with novel, nonparental barcode combinations expected only in “post shuffle” cells (Fig. 1E and fig. S1A). To genotype SVs at single-cell resolution, T7 IST is performed after fixation but prior to scRNA-seq, creating an RNA fingerprint of which barcode combinations are present in association with each single-cell transcriptome (36, 37) (Fig. 1E). Altogether, this strategy is designed to enable (i) multiplex SV generation in a population of mammalian cells, (ii) straightforward identification of the class and breakpoints of each induced SV, and (iii) efficient genotyping and quantitation of SVs, either in bulk (from total DNA or RNA) or at single-cell resolution (through scRNA-seq).

## Multiplex generation and haplotype-resolved mapping of thousands of SVs in mouse ESCs

As a proof of concept, we leveraged piggyBac (37) to randomly transpose a shuffle cassette library into the genome of an F1 hybrid C57BL6/6J × CAST/EiJ (BL6xCAST) male diploid mESC cell line at a high MOI (Fig. 1C) (38, 39). This cell line was chosen because (i) a heterozygous SNV or indel is present every ~150 bps, facilitating assignment of shuffle cassette integrations to one haplotype or the other (40); (ii) large rearrangements are probably less toxic in diploid versus haploid cells; and (iii) this mESC line can be differentiated into diverse cell types or organoids to facilitate study of cell type–specific SV effects.

**Fig. 2.**
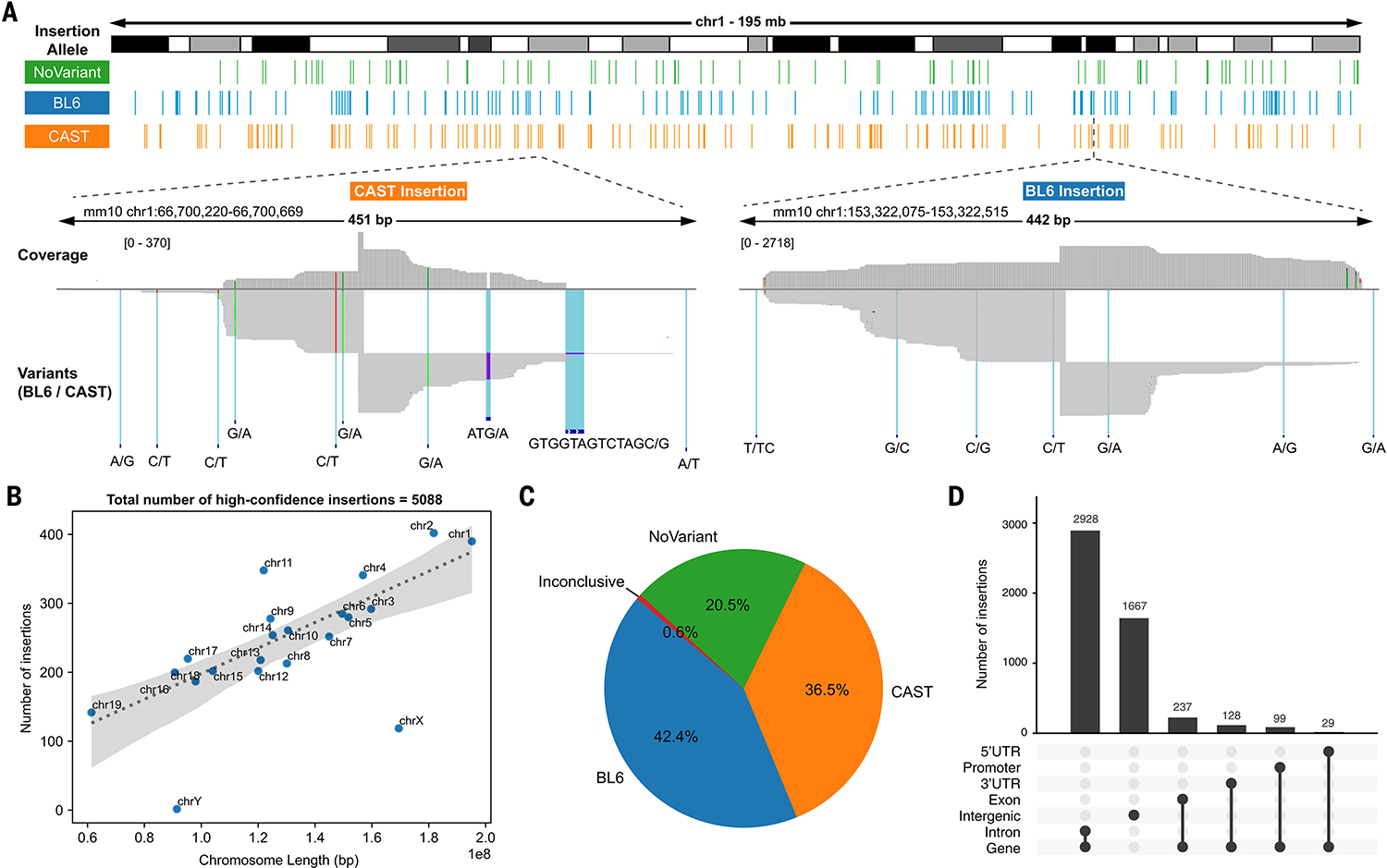
Allele-specific mapping of shuffle cassette insertions. (**A**) Insertion sites detected across chromosome 1 in a bottlenecked population of BL6xCAST mESCs, colored by allele. Insets depict pileups of sequencing reads from T7 transcripts for exemplary integrations to the CAST (left) or BL6 (right) haplotype. Alleles are distinguished by the presence of known variants between them. (**B**) Number of insertion sites with unique barcodes (*y*-axis) across chromosomes of varying lengths (*x*-axis). The dotted line indicates a linear regression model fit and the shaded gray areas indicate the 95% confidence interval. We have not corrected for copy number here as the X chromosome is single-copy in this male cell line. (**C**) Pie chart depicting the distribution of assignments to BL6 or CAST alleles for shuffle cassettes whose genomic coordinates were mapped with high confidence. (**D**) UpSet plot of intersection of shuffle cassette integration sites with genomic features.

After bottlenecking to ~100 founding clones followed by expansion, we estimated an average MOI of 123 though quantitative polymerase chain reaction (qPCR) (fig. S2, A and B). We identified 9416 parental barcode combinations in the bottlenecked population by amplicon-seq of shuffle cassettes (figs. S1A and S2C). We performed T7 IVT based mapping (37) on gDNA to identify the location and orientation of each shuffle cassette integration (Fig. 2A and fig. S2D). After filtering out those mapping ambiguously or to multiple locations, we retained 5088 barcoded shuffle cassettes, confidently mapped at bp resolution. chrX and chrY had lower insertion densities than that of autosomes, presumably due to their single copy in these male cells and difficulties mapping to the repetitive chrY (Fig. 2B). We used allele-specific SNVs and indels to assign nearly 80% of shuffle cassettes to either the BL6 or CAST haplotype (Fig. 2C and fig. S3). Shuffle cassettes largely mapped to introns and intergenic regions (Fig. 2D).

**Fig. 3.**
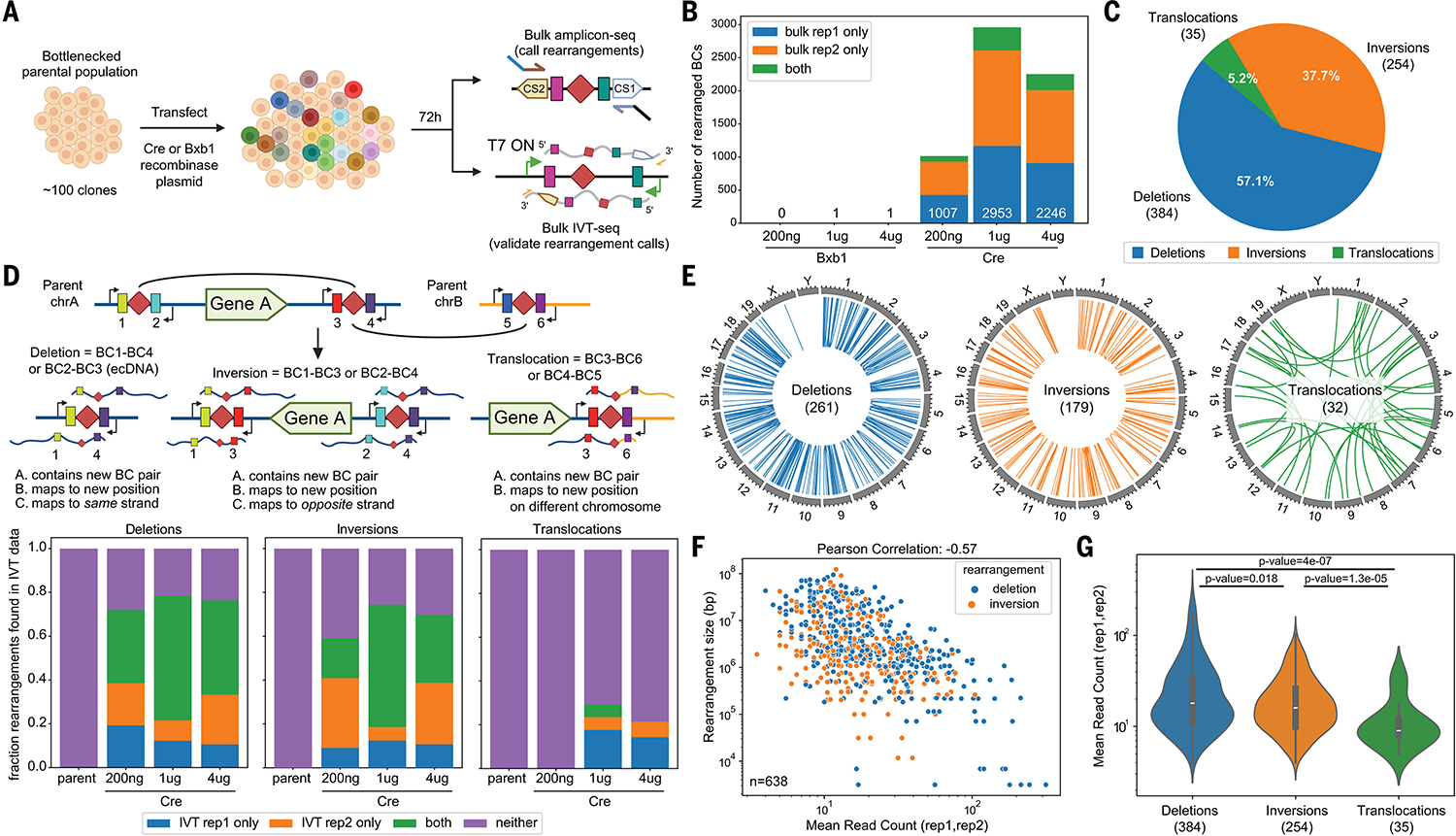
Multiplex induction and efficient genotyping of large-scale rearrangements throughout a mammalian genome. (**A**) Experimental schematic. (**B**) Number of novel (i.e., nonparental) barcode combinations at ≥2 UMI detected in each condition from amplicon-seq data. Different colors indicate those rearranged barcode combinations found in one technical replicate or both within each condition. (**C**) Pie chart showing the distribution of SV types that are detected in both technical replicates of a Cre transfection sample. SVs detected in multiple conditions are counted independently. (**D**) Schematic of approach for validation of SV calls using matched IVT-seq data from the same sample (top). The proportion of each SV type that is supported by at least one read in the IVT-seq data are depicted below. (**E**) Circos plots of the unique set of SVs that are shared between technical replicates of each sample. SVs detected in multiple conditions are counted once. (**F**) Scatter plot of rearrangement size (*y*-axis) versus mean read count (*x*-axis) for deletions and inversions detected at day 3. Pearson correlation is calculated between the log10 values of the two metrics. (**G**) Violin plots depicting the distribution of read counts for deletions, inversions, and translocations detected at day 3. Inset within each violin plot is a box plot of the distribution with the median value depicted as a white line, the length of the box depicts the interquartile range, and the whiskers depict the extent of the distribution. *P*-values are calculated using the nonparametric Mann-Whitney U test.

We next sought to induce and genotype SVs (Fig. 1). We transfected varying quantities of either a plasmid expressing Cre recombinase or, as a negative control, nontargeting Bxb1 recombinase, into cells derived from the bottlenecked population. At 72 hours post transfection (day 3), cells were harvested, gDNA isolated, and amplicon-seq of shuffle cassettes performed (Fig. 3A). As we hoped, while nonparental barcode combinations were nearly absent from the nontargeting Bxb1(+) condition, >5000 novel, nonparental barcode combinations were detected across Cre(+) conditions (Fig. 3B). As we sometimes detected both nonparental barcode combinations generated by a single recombination event (Fig. 1E and fig. S1, C and D), these were reduced to 4856 unique SVs. Approximately 50% of rearrangements between loxPsym sites are expected to result in shuffle cassettes with the same PBS on either side. These may be undetectable as a result of suppression PCR (41, 42), a challenge that we return to further below (fig. S1, C and D).

Our analyses suggest we induced and detected only a small fraction of the SVs that could potentially be generated from these bottlenecked cells. First, ~99.9% of amplicons in Cre(+) conditions matched “parental” barcode combinations, suggesting that each detected SV is rare within this cell population (fig. S4A). Second, most novel barcode combinations were not shared between technical replicates prepared from different gDNA aliquots from the same Cre(+) condition, nor across Cre(+) conditions. Thus, we would have likely detected many more SVs simply by processing more Cre-exposed cells from this same population of ~100 founding clones.

## Genome-Shuffle-seq induces thousands of unique SVs

For each novel barcode combination, we inferred the class and size of the corresponding SV based on the relative genomic coordinates and orientation of its parental shuffle cassettes (Fig. 1E and fig. S1, C and D). For the subset of SVs shared by both technical replicates of a Cre(+) condition (*n* = 673), 53% were observed in at least one other condition (fig. S4B), and deletions/inversions were much more common than translocations (Fig. 3C). However, if we consider all detected SVs (*n* = 6879), translocations comprised the majority (fig. S4C). We return to the interpretation of this difference further below.

SVs involving all chromosomes except chrY were detected (Fig. 3E and figs. S5 and S6). The number of SVs detected per chromosome was correlated with chromosome size (fig. S6, A and B). Some chromosomes appeared enriched or depleted for certain rearrangement classes (fig. S6, C to H).

For deletions/inversions, there was an inverse exponential relationship between SV size and abundance, the latter inferred by the number of reads supporting the novel barcode combination (Fig. 3F and fig. S4D). The subset of deletion/inversion SVs supported by both technical replicates (*n* = 638) had a read-counted weighted median event size of ~1 Mb, while the complete set (*n* = 3163) had a larger median event size ~2.5 Mb (fig. S4E). This may simply be because Cre recombination efficiency drops exponentially with genomic distance (17), although selection against large genomic deletions or inversions may also contribute.

To orthogonally validate SVs inferred from novel barcode combinations, we performed IVT-seq (37) on “post rearrangement” gDNA. Given the convergent orientation of the T7 promoters, IVT transcripts should span the novel barcode combination and flanking genomic sequence, thereby providing direct validation (Fig. 1B). For deletions and inversions, a substantial portion (~40 to 80%) of either the technically replicating (*n* = 638) or full (*n* = 3163) deletion sets were validated by 1+ IVT-seq read from the same condition (Fig. 3D and fig. S4F). By contrast, although translocations composed the majority of all detected SVs, fewer translocations (~5 to 30%) were validated (Fig. 3D and fig. S4F). Consistent with that, translocations were supported by substantially fewer reads than deletions/inversions in the amplicon-seq data in which each SV was originally detected (Fig. 3G and fig. S4G). Artifactual explanations such as chimeric PCR were ruled out by the dearth of reads supporting any type of SV, including translocations, in Bxb1(+) control cells (Fig. 3B). However, a simple alternative explanation is that many detected deletions/inversions were being generated recurrently even within a single condition/replicate, whereas detected translocations occur independently, lowering their read counts and precluding validation in independent aliquots of “post rearrangement” gDNA. Another possibility is that translocations were occurring at similar rates but were strongly selected against, either indirectly (through generalized Cre toxicity) or directly (through phenotypic consequences of the translocation itself).

Altogether, these results show that we can induce, detect, quantify, and characterize thousands of deletions, inversions, and translocations in a pool of cells in a single multiplex experiment with Genome-Shuffle-seq, without any single-cell cloning, genotyping or whole genome sequencing.

## SVs mediated by Cre at symmetric recognition sites are rapidly depleted from mESCs in vitro

To evaluate the stability of induced SVs, we sampled Cre(+) cells at days 5 and 7 post transfection, and sequenced shuffle cassette-derived amplicons (fig. S7 and methods). We observed a notable sharp decline, with almost no SVs detected by day 7 (fig. S7, C and D). We hypothesized this was due to the toxicity of Cre recombinase to mammalian cells, which is thought to impose a fitness cost in proportion to the number of target sites in the genome, in a p53-dependent manner (43–45). In our experiment, this might lead to poorly or untransfected cells outcompeting transfected cells by day 7. As a potential solution, tamoxifen-inducible Cre variants (CreERT2 and ERT2CreERT2) could be used to temporally restrict Cre activity, limiting toxicity (43, 46). To test this, we transfected parental cells with inducible Cre variants, treated with 0.5 μM tamoxifen for 24 hours at 1 day post transfection, collected samples at days 3, 5, and 7, and performed amplicon-seq of shuffle cassettes (fig. S7A). Both inducible Cre variants induced far fewer SVs than constitutive Cre and failed to facilitate survival of cells bearing SVs at day 7 (fig. S7, C and D). As an alternative strategy, we treated cells with the p53 inhibitor Pifithrin-α (20 μM) for 48 hours post Cre transfection. Treatment was limited to this timeframe as a result of the toxicity and adverse impacts of p53 inhibition on stem cell maintenance and differentiation (47, 48). However, although Pifithrin-α treatment increased the number of SVs detected at day 3, their abundance sharply declined by day 5 (fig. S7, E and F).

An alternative explanation is that Cre-induced SVs were causing fitness defects, such that cells lacking SVs outcompete them. To evaluate this hypothesis, we sought to clonally expand “post rearrangement” single cells. We co-transfected either Cre or Bxb1, together with a Cre-reporter that conditionally expresses red fluorescent protein (RFP), into the bottlenecked parental population, and treated with either Pifithrin-α or no drug for 48 hours post transfection. On day 3, 720 RFP-positive, Cre-treated cells were sorted into single wells that contained either Pifithrin-α or no drug (fig. S8, A to C). Pifithrin-α treatment markedly increased the likelihood of growth after sorting of Cre-treated cells, consistent with p53 inhibition reducing cell death (fig. S8D). However, no SV-supporting barcode combinations were detected upon amplicon-seq of shuffle cassettes in 86 single cell clones. The median number of parental shuffle cassettes detected per Cre-treated sample was lower than that for Bxb1 samples (fig. S8E), suggesting that clones with higher numbers of integrated shuffle cassettes may be selected against after Cre transfection.

## Genome-Shuffle-seq with Bxb1 recombinase and in human cancer cells

The rapid depletion of Cre-induced SVs represents a major limitation, as it precludes the isolation of subclones for functional analysis of induced SVs. As our attempts to address this limitation by restricting Cre activity, inhibiting p53, and/or circumventing clonal competition were unsuccessful, we sought to develop a version of Genome-Shuffle-seq relying on Bxb1 rather than Cre recombinase, as well as to expand our evaluation to include human K562 cells, which derive from a p53-null, chronic myelogenous leukemia.

Bxb1 recombinase, which is less toxic than Cre recombinase in mammalian cells (49, 50), utilizes two heterotypic recombinase sites, attB and attP. Recombination between these sites results in novel attL and attR sites, which are resistant to further Bxb1-mediated recombination. Therefore, SVs resulting from Bxb1 acting at attB/attP sites are expected to be more stable than those resulting from Cre acting at loxPsym sites. Although the use of heterotypic sites means that only 50% of all possible pairs of integrated shuffle cassettes can recombine with one another, this is balanced by an advantage with respect to detection rate. In particular, the directional nature of these Bxb1-target sites ensures that all recombined shuffle cassettes will be flanked by heterotypic PBSs, which eliminates the aforementioned concern about suppression PCR (41, 42) such that all induced SVs are detectable (fig. S1, C and D, and fig. S9).

We introduced a Bxb1 attB/P shuffle cassette library to both mESCs and K562s, and in parallel, a loxPsym shuffle cassette library into K562s, at high MOI (fig. S10A). As before, we expanded bottlenecked founder populations and mapped insertion sites with IVT-seq (37). We confidently mapped the precise genomic locations of 904, 3644, and 2688 shuffle cassettes in attB/P+ mESCs, attB/P+ K562s and loxPsym+ K562s, respectively (fig. S10B). 74% of mapped attB/attP cassettes in mESCs were confidently assigned to either the BL6 or CAST haplotype, and mapped attB versus attP cassettes were present in roughly equal proportions (fig. S10, C to E).

**Fig. 4.**
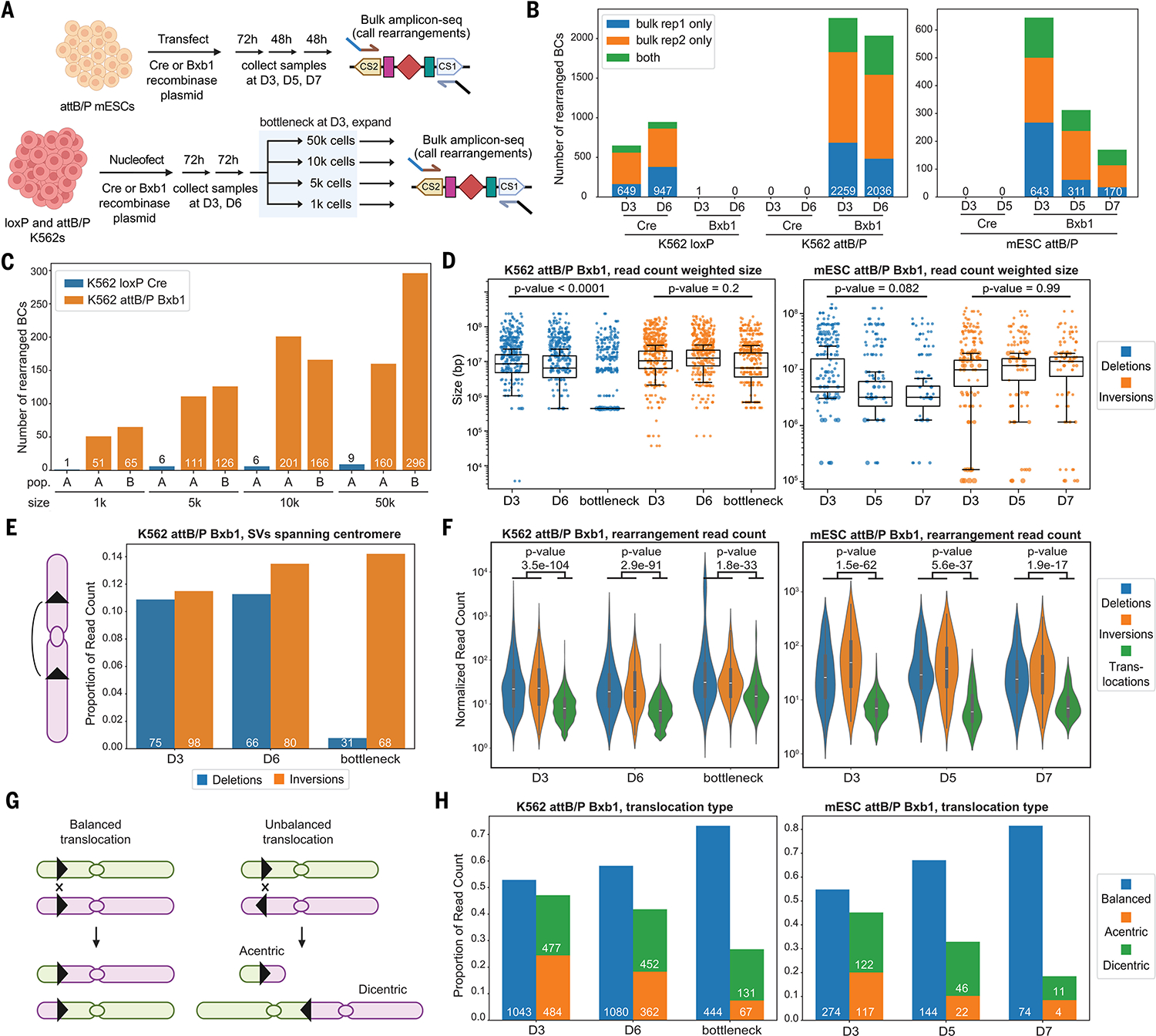
Bxb1 recombinase mediates induction of long-lived SVs in two mammalian cell types and reveals selection pressures. (**A**) Experimental schematic. Libraries of shuffle cassettes bearing Bxb1 attB/P sites or Cre loxP sites were integrated into human K562s or mouse ESCs. Rearrangements were induced by transient transfection of a recombinase-expressing plasmid and cells were collected at the indicated time points for rearrangement detection by amplicon-seq. (**B**) Number of novel (i.e., nonparental) barcode combinations at ≥2 UMI detected in each experimental condition by amplicon-seq. Colors indicate those rearranged barcode combinations found in one technical replicate or both. (Left) K562s, bearing either loxP or attB/P shuffle cassettes, exposed to either Cre or Bxb1 recombinase, from day 3 or 6. (Right) mESCs, bearing attB/P shuffle cassettes, exposed to either Cre or Bxb1 recombinase, from days 3, 5, or 7. Note that technical replicates were performed for Bxb1-treated populations but not for Cre-treated populations. (**C**) Number of novel barcode combinations at ≥2 UMI detected in each sample of the indicated K562 bottlenecked populations. The letters A and B correspond to two independent bottlenecked populations that were sampled for Bxb1-treated attB/P+ K562s per starting cell size, whereas only one bottlenecked population (A) was sampled for Cre-treated loxPsym+ K562s. Note that for each sample, data are from a single technical replicate from cells collected after expansion. (**D**) Log-scale boxplots of inversion and deletion sizes, weighted by read count, at days 3 or 5 or post bottlenecking/expansion. The horizontal solid line indicates the median, the length of the box depicts the interquartile range, and the whiskers depict the extent of the distribution minus outliers. The underlying distribution is depicted by the overlaid points, with the size of each bubble reflecting the relative read count. Depicted *P*-values were calculated using a bootstrap analysis with 10,000 iterations, resampling the distribution with replacement. (Left) K562s. (Right) mESCs. (**E**) Barplots depicting the proportion of total deletion and inversion reads at each time point that reflect a recombination between sites inferred to reside on different arms of the same chromosome. Number of events are indicated at the base of each bar. (**F**) Log-scale violin plots depicting the distribution of read counts for the complete set of deletions, inversions, and translocations detected at the indicated time points. Inset within each violin plot is a box plot of the distribution with the median value depicted as a white line, the length of the box depicting the interquartile range and the whiskers depicting the extent of the distribution. Depicted *P*-values were calculated using the nonparametric Mann-Whitney U test, comparing the read count distributions of translocations, deletions, and inversions. (Left) K562s. (Right) mESCs. (**G**) Schematic depicting the formation of balanced and unbalanced translocations. Unbalanced translocations can lead to formation of acentric or dicentric chromosomes. (**H**) Barplot depicting the proportion of total translocation reads at each indicated time point that are inferred to derive from balanced, acentric, or dicentric translocations. Number of events of each type are indicated at the base of each bar.

Once these lines were established, we transiently transfected them with Cre or Bxb1 expressing plasmids (Fig. 4A). After transfection, mESCs were sampled at days 3, 5, and 7, and K562s at days 3 and 6. We also bottlenecked Bxb1(+) attB/P+ K562s at day 3 to 50,000, 10,000, 5000, or 1000 cells, harvesting two independent replicates per bottleneck size after expansion. For Cre(+) loxPsym+ K562s, we similarly bottlenecked at day 3, expanded, and harvested, but without replicates. We extracted gDNA from all samples and performed amplicon-seq of shuffle cassettes.

Hundreds to thousands of novel, rearrangement-indicative barcode combinations were detected in cell lines transfected with a targeting recombinase, with close to zero background in controls transfected with a nontargeting recombinase (Fig. 4B). In both Bxb1(+) attB/P+ cell lines, novel barcode combinations flanked attL or attR sites, rather than attB or attP sites (fig. S11A). 100% of novel barcode combinations in K562s (*n* = 6394) and mESCs (*n* = 1399) could be matched to pairs of parental attB and attP sites. Overall, these results highlight the generalizability of Genome-Shuffle-seq to diverse mammalian cell lines and SSR systems.

## SVs mediated by Bxb1 at asymmetric recognition sites are tolerated and survive bottlenecking

In contrast to their complete depletion after several days in Cre(+) loxPsym+ mESCs, rearranged barcodes continued to be detected in Bxb1(+) attB/P+ mESCs at day 7 (fig. S7C versus Fig. 4B). Consistent with this, the ratio of amplicon reads bearing rearranged versus parental barcode combinations decreased by >99.99% in Cre(+) loxPsym+ mESCs, but only by ~75% in Bxb1(+) attB/P+ mESCs, by day 7 (fig. S7D versus fig. S11B). These results indicated that SVs induced by Bxb1 at asymmetric recognition sites are much better tolerated than SVs induced by Cre at symmetric recognition sites, at least in mESCs.

In human K562 cells transfected with a targeting recombinase, the number of rearranged barcode combinations as well as the ratio of rearranged versus parental barcode combinations remained relatively stable at days 3 and 6 (Fig. 4B and fig. S11C). Furthermore, Bxb1 appeared more effective at inducing SVs than Cre (fig. S11C), potentially reflecting its greater efficiency in mammalian cells (51).

To summarize the contrast between cell lines, both Cre- and Bxb1-mediated SVs persisted for 6 days in K562 cells, whereas in mESCs, SVs were either completely (Cre) or partially (Bxb1) depleted within a week. Possible explanations for this difference include the following: (i) p53-null K562 cells are less sensitive to Cre toxicity; (ii) K562 cells divide more slowly than mESCs, such that the recombinase-expressing plasmid may still be present and inducing new rearrangements at later time points; and/or (iii) K562 cells are grown in suspension, which increases the chance of dead/dying cells to contaminate the sample, in contrast to adherent mESCs with which unhealthy cells are lost in the supernatant.

To distinguish between these possibilities, we examined the number of rearrangements in recombinase-treated K562 populations that had undergone bottlenecking and expansion (Fig. 4A). Here, the contrast between recombinases was stark, possibly because these conditions are the least prone to contamination by dead or dying cells. Whereas hundreds of SVs were readily detected in Bxb1(+) attB/P+ K562s following bottlenecking and expansion, Cre(+) loxPsym+ exhibited a substantial reduction in the number of surviving rearrangements (Fig. 4C and fig. S11D).

Overall, these results suggest that Cre and/or the rearrangements that it induced at symmetric recognition sites are toxic in not only mESCs but also K562 cells. By contrast, Bxb1 and the rearrangements that it induced at asymmetric recognition sites were markedly better tolerated and more stable in at least two mammalian cell lines.

## Bxb1-mediated SVs exhibit signatures of selection

We detected deletions, inversions, and translocations in both Bxb1(+) attB/P+ K562s and mESCs (fig. S12). Although these were short, culture-based experiments in diploid (mESC) or pseudotriploid (K562) cell lines, we sought to evaluate whether any particular class of SVs was rapidly enriched or depleted as a result of fitness effects. Focusing first on deletions/inversions, we observed a clear inverse correlation between SV event size and abundance, as expected due to dependence of shuffle cassette recombination on proximity (fig. S13). The abundance-weighted size distribution of deletions, but not inversions, was reduced over time and/or with bottlenecking (Fig. 4D). Further examination suggested that this was partly attributable to centromere-spanning deletions, which presumably compromise chromosome segregation. In particular, centromere-spanning deletions were strongly depleted from bottlenecked K562 populations whereas centromere-spanning inversions were enriched (Fig. 4E).

As with Cre(+) loxPsym+ mESCs, more unique translocations were observed in Bxb1(+) attB/P+ K562s and mESCs than in deletions/inversions, but the underlying read counts once again revealed translocations to be much less abundant (Fig. 4F). Moreover, the diversity and abundance of translocations diminished over time and/or with bottlenecking (fig. S12). To further investigate this, we classified each translocation SV as (i) balanced; (ii) unbalanced, leading to an acentric chromosome; or (iii) unbalanced, leading to a dicentric chromosome (Fig. 4G). Although balanced and unbalanced translocations occurred at roughly equal frequencies, the proportion of unbalanced translocations of both subtypes decreased over time in both cell types (Fig. 4H). Furthermore, acentric chromosomes were depleted more rapidly than dicentric chromosomes (Fig. 4H), presumably because dicentric chromosomes can survive a centromere crisis whereas acentric chromosomes, lacking a centromere, cannot (52).

Taken together, these results show that cells bearing Bxb1-mediated SVs survive long enough to experience fitness effects caused by SVs; our results also highlight the potential for selective pressures on individual SVs generated by Genome-Shuffle-seq to be quantified.

## Hundreds of ecDNAs are launched and detected by Genome-Shuffle-seq

Each Bxb1-mediated intrachromosomal deletion between directly oriented sites is expected to leave a genomic scar composed of a shuffle cassette bearing a novel barcode combination, but also to create a single extrachromosomal DNA circle (ecDNA) composed of the deleted sequence and a shuffle cassette bearing the reciprocal barcode combination (Fig. 1E and fig. S14A). Moreover, although both species are expected to be present in equal stoichiometry at the time of their formation, ecDNAs may be depleted over time as a result of their reliance on asymmetric segregation for inheritance. Of note, recombination between shuffle cassettes on sister chromatids after genome replication could potentially yield duplications that are indistinguishable from ecDNAs based on amplicon sequencing (17). However, because the sites involved are in trans, such duplications are expected to arise at much lower frequencies than deletions and are not considered for the analyses that follow.

For most deletions induced in Bxb1(+) attB/P+ mESCs and K562s, we readily detected reciprocal barcode combinations derived from a “matched” genomic scar and ecDNA species in the same biological sample (fig. S14B). Reciprocal barcode combinations were initially found at roughly equal frequencies. But as predicted, ecDNA barcode combinations were depleted over time, both overall and when only considering putative deletions for which both members of a reciprocal pair were detected (fig. S14, C and D).

Notably, although results from Bxb1-mediated Genome-Shuffle-seq followed expectations for bona fide mammalian ecDNAs, results from Cre-mediated Genome-Shuffle-seq did not. First, there were lower proportions of cases in which we detected both genomic scar and ecDNA-derived barcode combinations in the same sample (fig. S14, B and C). Second, in both mESCs and K562s, barcodes derived from ecDNAs were detected at approximately two times higher read counts than barcodes derived from genomic scars, rather than the expected 1:1 stoichiometry (fig. S14D). Finally, after weighting by abundance, the inferred sizes of Cre-mediated ecDNAs tended to be larger than the inferred sizes of Cre-mediated deletion scars (fig. S14E).

These observations are not easily explained by differential recovery of ecDNA versus genomic DNA, fluctuations in ecDNA copy number, or misattribution of some duplications to ecDNAs, as these features are shared between Bxb1- and Cre-derived ecDNAs. However, there were two key differences: Symmetric sites (loxPsym) were used with Cre and asymmetric sites (attB/P) with Bxb1, and post-recombination loxP sites (including the symmetrical version used here) can undergo further recombination events whereas the attL/R sites that result from Bxb1-mediated recombination of attB/P sites cannot. These differences may result in the formation of novel genomic structures with Cre-based Genome-Shuffle-seq that are not easily decoded from shuffle cassette combinations. Additionally, given that few Cre-mediated recombinants are detectable at later time points (Fig. 4C and fig. S14B), it is possible that some of these excess ecDNA barcode combinations originated from dead or dying cells, which may not accurately represent the distribution of induced SVs in (still) living cells.

In sum, these results indicate that Bxb1-mediated Genome-Shuffle-seq may be a powerful tool to generate and study hundreds of ecDNAs launched from deletions throughout the genome.

## Genotyping induced SVs at single-cell resolution

Genome-Shuffle-seq was designed to facilitate genotyping of induced SVs on widely available scRNA-seq platforms (Fig. 1E). Specifically, after fixation and T7 IST (36, 37), cells are expected to contain both endogenous mRNAs and T7-derived transcripts that span shuffle cassette barcode pairs. On the 10X Genomics platform, it should be possible to capture both sets of transcripts to a common cell barcode (cell BC) through 3′ scRNA-seq with feature barcoding (fig. S15A).

To test this scheme, we co-transfected loxPsym+ mESCs with a plasmid expressing Cre and a Cre-reporter that conditionally expresses RFP, sorted RFP+ cells at 72 hours, and then performed methanol fixation, T7 IST, and scRNA-seq. For this experiment, we combined Pifithrin-α-treated and untreated cells and included an independent sample from parental cells as a control (fig. S15B). We recovered ~15,000 and ~19,000 profiles (T7 IST + scRNA-seq) from Cre-treated and parental samples, respectively. To assess rearrangements, we compared barcode combinations observed in (T7 IST + scRNA-seq) data to parental barcode pairs, which identified 1123 novel barcode combinations. To rule out artifacts of library construction, we performed a downsampling QC analysis; at similar sequencing depths, 280 and 0 novel barcode combinations were detected in Cre-treated parental scRNA-seq profiles, respectively (fig. S15C).

**Fig. 5.**
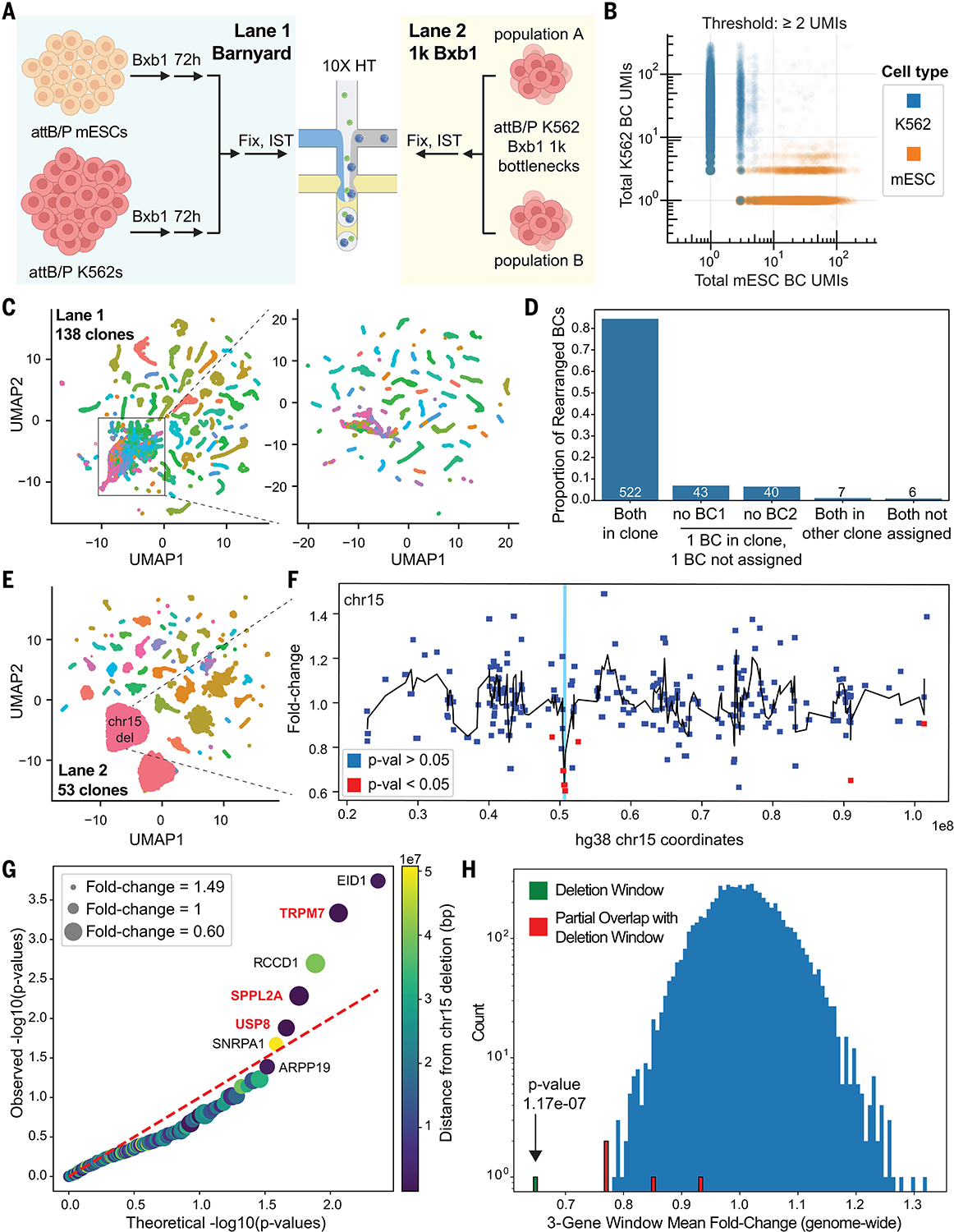
Detection of SV identity and associated gene expression changes in single cells. (**A**) Experimental schematic. The indicated populations were mixed prior to fixation and in situ transcription (IST) with T7 polymerase, after which cells were loaded onto two independent lanes of a 10X Genomics high-throughput (HT) chip. (**B**) Barnyard plot of total T7 UMIs detected in mESCs or K562 cells at a threshold of ≥2 UMI per barcode pair. Each point represents a cell, colored by cell-type assignment. The *x*-axis represents counts from shuffle-cassette barcodes originating from mESCs and the *y*-axis represents counts from shuffle-cassette barcodes originating from K562s. (**C**) In Lane 1, 11,252 cells were assigned to 138 independent clonotypes based on the complement of T7 barcodes detected within them. Here, these cells are visualized in UMAP space, colored by clone assignment. The plot on the right was generated following iterative dimensionality reduction on the indicated subset of cells from the global UMAP on the left. (**D**) Barplot depicting the fraction of rearranged barcodes detected in cells that are congruent with the clonotype assignment of that cell. Some barcodes were not assigned to a clone. (**E**) In Lane 2, 14,727 cells were assigned to 53 clonotypes. Here, these cells are visualized in UMAP space and colored by clone assignment. The clone to which cells bearing the most frequent inferred rearrangement in this dataset, a ~447 kb deletion on chromosome 15, is labeled. (**F**) Map of fold changes in gene expression versus genomic coordinates for genes across chromosome 15. Genes with nominally significant (i.e., uncorrected) decreases in expression (one-sided Wilcoxon Rank Sum Test) are in red and other genes are in blue. The *y*-axis depicts fold change in expression between the cells in which the barcodes corresponding to the deletion were detected versus cells from the same clonotype in which parental barcode combinations were detected. The vertical region shaded in light blue indicates the span of the inferred ~447-kb deletion. The black line indicates a moving average of fold change with a window size of three genes. (**G**) Quantile-quantile plot of observed log10 *P*-values from a one-sided Wilcoxon Rank Sum Test of fold changes against expected −log10 *P*-values from a uniform distribution for genes across chromosome 15, in cells from a single clonotype inferred to either have or not have the ~447-kb deletion. The red dotted line represents the expected relationship under the null hypothesis. Point size is proportional to the decrease in gene expression (1 per fold change) for that gene in cells with the rearrangement. Points are colored according to their proximity to the deletion. Names of genes encompassed by the deletion are colored red. (**H**) Histogram of the mean fold changes for rolling windows of three genes throughout the genome, comparing the same sets of cells, i.e. those from a single clonotype inferred to either have or not have the ~447 kb deletion on chromosome 15. The three-gene window fully overlapping with the deletion (*USP8, TRPM7, SPPL2A*) is indicated in green, and windows with one or two of these genes are indicated in red. The depicted *P*-value (uncorrected) is calculated from the z-score of the deletion window with the rest of the normal distribution.

Although this preliminary experiment suggested that our scheme was working as intended, two aspects required further investigation. First, because we permeabilize cells for T7 IST, our protocol may be more susceptible to ambient RNA contamination, a pervasive issue in scRNA-seq (53, 54). Second, co-capturing SV identity should allow us to test for gene expression changes caused by induced rearrangements. To explore these aspects, we conducted a second Genome-Shuffle-seq scRNA-seq experiment (Fig. 5A). In a first tranche (“Lane 1”), we performed a “barnyard” experiment by mixing Bxb1-treated attB/P+ mESCs (mouse) and K562s (human) prior to fixation and IST to quantify ambient T7 transcripts. In a second tranche (“Lane 2”), we mixed two independent populations of Bxb1-treated attB/P+ K562s that had previously been bottlenecked to 1000 cells, expanded, and subjected to shuffle cassette amplicon-seq (Fig. 4C). In theory, cells with SVs passing the bottleneck should be expanded in the profiled population, potentially providing power for detecting gene expression changes caused by individual SVs.

After filtering on mitochondrial content and transcriptome unique molecular identifier (UMI) counts, requiring detection of 1+ T7 transcripts, and performing doublet removal, we recovered 18,418 (11,113 K562 and 7305 mESC) and 20,798 K562 single-cell profiles from the first and second tranches, respectively (fig. S16, A and B). We also recovered a median of 51 and 56 T7 UMIs, which reflected a median of 36 and 35 unique shuffle barcode combinations, per cell (fig. S16, C to F). A median of 3965 and 6465 transcriptome UMIs were detected and these counts were correlated with the number of T7 UMIs detected per cell (fig. S16, G and H). In the barnyard experiment, >75% of T7 barcodes were associated with a single cell of the expected species, and a simple threshold of ≥2 UMIs per T7 barcode combination per cell was sufficient to achieve >98% species specificity, mitigating concerns about ambient T7 transcripts (Fig. 5B and fig. S17, A and B).

To further investigate the sensitivity of T7 barcode detection, we performed iterative clustering on combinations of barcodes observed in single cells from the barnyard experiment, to yield “clonotypes.” We anticipated that these clonotypes would correspond to individual cells that survived the original bottlenecking of the parental population (fig. S10A). Indeed, a more stringently filtered set of 11,252 cells clustered neatly in UMAP (uniform manifold approximation and projection) space into 138 species-coherent clonotypes, based solely on their complement of T7 IST-derived barcode combinations (Fig. 5C and fig. S18, A and C to E). A precision-recall analysis found that once again a simple threshold of ≥2 UMIs per T7 barcode combination per cell was sufficient to achieve high specificity, now with respect to clonotype rather than species (fig. S17C).

To assess rearrangements in the barnyard experiment, we compared the shuffle cassette barcode combinations observed in T7 IST + scRNA-seq data to parental barcode pairs. We identified 3098 novel barcode combinations, 618 of which were found at ≥2 UMIs in cells of the correct species that were assigned to a clonotype (fig. S19A). The barcodes contributing to these novel combinations were highly congruent with expectation based on clonotype identity. Specifically, 84% involved a pair of parental barcodes from the same clonotype, while 13% involved one parental barcode from the same clonotype, and one parental barcode that was unassigned to any clone (Fig. 5D). Collectively, these data suggest that we can detect, map and confidently assign induced SVs to single cells with associated transcriptomes.

For Lane 1, most rearrangements were only detected in only one cell (range 1 to 115) and most cells only contained a single detected rearranged BC pair (range 1 to 3) (fig. S19, C to E). Similar to our analysis shown in Fig. 3, we were able to infer the nature of each SV based on the parental locations of the barcodes contributing to each novel pair (fig. S20A). As before, deletions and inversions were more replicable across samples than translocations (fig. S20B).

Similarly analyzing the Lane 2 bottlenecking experiment, we assigned 14,727 cells to 53 clonotypes (Fig. 5E fig. S18, B and F), and detected 584 rearranged barcodes at ≥2 UMIs across 566 cells that were correctly assigned to a clone (fig. S19B). These rearranged barcodes corresponded to 24 unique novel barcode combinations, of which 23 were detected in bulk amplicon-seq data from the same bottlenecked population. The inferred abundances of these 23 combinations were markedly higher than those of other novel combinations detected by bulk amplicon-seq (fig. S20, B and C).

Despite the bottleneck, most of these 24 novel barcode combinations were detected in fewer than 10 cells (fig. S19, D and F), precluding the sensitive detection of gene expression changes consequent to a given SV. However, there was one exception, a novel barcode combination reflecting the genomic scar of a ~447-kb deletion on human chr15, detected at ≥2 UMIs in 465 cells that were assigned to the correct clonotype and had associated transcriptomes (fig. S21, A and B). Three genes within this interval were detected in >10 cells in the entire dataset (*USP8, TRPM7, SPPL2A*). We compared expression of these genes in rearrangement-bearing cells with 680 cells from the same clonotype in which the parental barcodes of this rearrangement were instead detected (Fig. 5E). Each of the three genes exhibited a ~33% decrease in expression, precisely matching expectation for deletion of a single allele of a triploid chromosome such as chr15 in K562s (Fig. 5F). These three genes, when considered individually, were nominally significant, as were two other genes located ~1.5 Mb away (*EID1* and *ARPP19*) (Fig. 5, F and G). It is plausible that some long-range regulatory elements for the latter two genes lie within the ~447 kb deletion, although this was not obvious from public datasets (fig. S21A).

To assess robustness, we performed a downsampling analysis, reducing the number of cells bearing the ~447 kb deletion used in the comparison. Although statistical significance unsurprisingly declined, the estimate of a ~33% reduction in expression for each gene was highly stable to downsampling (fig. S21C). Furthermore, if we consider the mean fold change of a rolling window of three genes, scanning throughout the entire genome, the trio of genes encompassed by the deletion is a clear, significant outlier (Fig. 5H). Taken together, these results demonstrate that Genome-Shuffle-seq is compatible with scRNA-seq, and that co-assays of single cell transcriptomes and SV-informative barcode combinations can facilitate the quantification of gene expression changes resulting from induced SVs.

## Discussion

We describe a method for the inducible generation and facile characterization and quantitation of thousands of mammalian SVs within a pool of cells. We show how the method can be used to produce synthetic ecDNAs and to quantify selection acting on the landscape of induced SVs. Finally, we demonstrate that SV identities can be captured alongside single-cell transcriptomes, and establish the potential for such data to reveal changes in gene expression caused by induced SVs.

In its current form, Genome-Shuffle-seq has several key limitations: First, capture rates of T7-derived transcripts in scRNA-seq remain limited; higher rates would facilitate more comprehensive detection of induced SVs, and ideally a complete in silico karyotype of each profiled cell. Improvements may be possible by modifying cassette design and/or the co-assay protocol.

Second, although cells bearing Bxb1-induced SVs are clearly surviving, the proportion of cells bearing 1+ inferred SVs remains low (~4%). Avenues to improve this include increasing the rate of SV formation (e.g., longer Bxb1 exposure, more shuffle cassettes). Alternatively, a conditional selection marker reconstituted upon shuffle cassette recombination (16, 17) would ensure all cells surviving the initial selection contained at least one SV.

Third, consequent to above as well as the sparsity of scRNA-seq data, power to detect gene expression changes caused by individual SVs remains limited. Performing Genome-Shuffle-seq co-assays with higher-throughput, lower-cost modalities for scRNA-seq may be necessary to adequately power screens of the functional consequences of hundreds to thousands of induced SVs on gene expression (55, 56, 57).

Notwithstanding these limitations, we believe that Genome-Shuffle-seq lays the foundation for large-scale, single-cell genotype-to-phenotype screens of the impact of thousands to millions of mammalian SVs and ecDNA species on gene expression, chromatin structure and genome organization, analogous to Perturb-seq or CROP-seq (58, 59). As a related approach, the targeted introduction of shuffle cassettes to individual mammalian genomic loci, for instance by bottom-up assembly (60), would facilitate the dissection of regulatory element interactions and locus architecture in specifying gene regulation. Genome-shuffle-seq could also readily be adapted to study the cell type–specific impact of SVs by differentiating a single engineered population into in vitro multicellular models or in vivo using whole-organism models. Of note, in related work conducted independently, Koeppel, Ferreira, and colleagues describe a complementary strategy for the “randomization” of mammalian genomes with engineered SVs using highly multiplexed prime editing-mediated insertion of loxPsym sites (61). Beyond enabling the more systematic study of SVs and ecDNAs, these approaches may also serve as an entry point for the engineering of a “minimal genome” comprising the essential complement of genetic information required for propagation of any mammalian cell, potentially useful as a universal chassis for cell-based therapy (62).

## Materials and Methods

### Shuffle cassette library cloning

The sequence of the shuffle cassettes was ordered as single-stranded oligonucleotides from Integrated DNA Technologies (IDT) with degenerate bases at the appropriate sites to serve as barcodes (Fig. 1B). We employed the variant Bxb1-GA attB and attP sites, as they were previously shown to be more efficient than the canonical GT variant in mammalian cells (51). The pool was PCR amplified using Q5 polymerase (NEB M0492S) for eight cycles with primers that contained overhangs for subsequent cloning. PCR products for loxPsym library cloning were run on a polyacrylamide gel (PAGE) and the band at the appropriate size was excised and purified. Bxb1 attB/P library PCR products were cleaned up (1X) with Ampure XP beads (Beckman A63882). Purified product was Gibson cloned into a previously described PiggyBac transposon vector (37) using standard protocols in a 10 μl reaction (NEB E2621S). 2.5 μl of the Gibson reaction was electroporated into 25 μl of 10-beta electrocompetent *E. coli* (NEB C3020K). The transformed library was grown overnight in 50 mL of liquid Luria Broth (LB) + 50 μg/mL Ampicillin and purified using the ZymoPURE II Plasmid Midiprep kit (Zymo D4200) according to manufacturer’s instructions.

All primer sequences are listed in table S1.

### Cell culture and integration of Shuffle Cassette Library

BL6xCAST mESCs were cultured at 37°C and 5% CO2 on standard tissue culture treated plates coated with 0.1% gelatin in “80/20” medium as previously described (60). To integrate the Shuffle Cassette PiggyBac transposon library at high MOI, we reverse transfected 4 μg of library DNA with 200 ng of a PiggyBac Puro-GFP helper plasmid (63) and 200 ng of a plasmid expressing the hyperactive transposase hyPBase (64) using Lipofectamine 3000 (Thermo L3000001) into 1 million cells. 3 days post transfection, cells were selected with 2 μg/mL Puromycin. Surviving cells were bottlenecked to approximately 100 founders by dilution and expanded.

K562s were cultured at 37°C and 5% CO2 on standard tissue culture treated plates in RPMI medium supplemented with 10% FBS (Hyclone). To integrate the Shuffle Cassette PiggyBac transposon library at high MOI, we nucleofected 5 μg of library DNA with 250 ng of a PiggyBac Puro-GFP helper plasmid and 250 ng of a plasmid expressing the hyperactive transposase hyPBase using an Amaxa 4D nucleofector (Lonza, SF cell line kit, program FF-120). Cells were selected with 2 μg/mL Puromycin, starting 3 days post nucleofection. Surviving cells were bottlenecked to approximately 100 founders by dilution and expanded.

### MOI estimation by qPCR

Genomic DNA was extracted from cells using the Qiagen DNeasy kit. MOI of the shuffle cassette (primers oSP990-oSP993) and Puro-GFP (primers oJBL043-oJBL044) were measured relative to two genomic targets Tfrc (primers oJBL0276-oJBL0277) and Tert (oJBL0280-oJBL0281) which are expected to have two copies in these cells (63). qPCR was performed using 2X PowerUp Sybr Master Mix (Thermo A25742) with 50 ng of genomic DNA as template per 10 μl reaction with 0.5 μl of 5 μM primer mix. qPCRs were run on a BioRad CFX Opus Real Time PCR instrument in triplicate. Cycle threshold values were averaged across triplicates and copy number was estimated using the ΔCT method relative to each genomic target independently, corrected for the presence of two genomic copies and then averaged across the two targets.

All primer sequences are listed in table S1.

### Recombinase plasmid transfection and cell population maintenance for shuffle experiments

Recombinase expression plasmids used in this study are pCAG-iCre (Addgene #89573), pCAG-CreERT2 (Addgene #14797) and pCAG-ERT2-CreERT2 (Addgene #13777) and pCAG-Bxb1 (pSP0722, modified from Addgene #51271).

For mESC experiments, between 200,000 and 350,000 cells were reverse transfected with the specific recombinase plasmid using Lipofectamine 3000 (Thermo L3000001) in 6-well plates with the exception of the data shown in fig. S7, E and F, which was generated from a scaled-down version of this protocol performed in 12-well plates in which 300 ng of Cre plasmid was transfected into ~66000 cells. For experiments depicted in fig. S7, C and D, 1 μg of the respective Cre plasmid was transfected. Briefly, DNA and P3000 reagent (2x DNA amount, 8 μl for 4 μg etc.) were mixed with 125 μl of OptiMEM. In a separate tube, Lipofectamine reagent (3x DNA amount, 12 μl for 4 μg etc.) was mixed with 125 μl of OptiMEM. The two tubes were mixed and allowed to sit at room temperature for at least 20 min. Transfection mix was added to a gelatinized well of a 6-well plate and cells were added on top in 2mL of medium. Medium was changed every day, and treatments such as 0.5 μM Tamoxifen (Sigma) and 20 μM Pifithrin-α (Sigma) were performed at the specified times. For the Pifithrin-α experiment in which Cre-transfected cells were cultured for longer than 72h, the cell population was split using Accutase (Gibco) and ~30% of the cell population was transferred to a new plate at day 3. For the inducible Cre variant experiments, cell population was split, and 100,000 cells were transferred to a new plate at day 3 and day 5. For all experiments, genomic DNA was prepared from a minimum of 25% of the cell population.

For K562 experiments, 200,000 cells were nucleofected with 1 μg of the respective recombinase plasmid using the Lonza 4D strips in a 20 μl reaction according to the manufacturer’s protocol (V4XC-2032). Cells were incubated after pulsing at room temperature for approximately 10 min before plating. Cells were split at day 3 post-nucleofection and 30% of cells were carried forward. The entire cell population was harvested on day 6. Specified cell amounts were plated 3 days post nucleofection in a fresh 12-well plate to derive K562 bottlenecked populations. Bottlenecked populations were expanded to a 6-well plate when saturated and samples were collected once saturated in the 6-well plate (>8 days post-nucleofection). For all experiments, genomic DNA was prepared from a minimum of 25% of the cell population.

### IVT-seq library construction

An experimental protocol for mapping integration sites using T7 IVT on genomic DNA has been described recently by our group (37). In this study, we largely followed this protocol with some minor modifications. The same protocol was followed both for shuffle cassette insertion site mapping from the parental population and for rearrangement call validation post Cre transfection. Genomic DNA purified using the Qiagen DNeasy kit was used as template for all IVT reactions. For loxPsym+ mESC samples, 300 ng of template, for loxPsym+ K562, attB/P+ mESC and attB/P+ K562, 500ng of template was used per reaction. T7 IVT was performed using the HiScribe T7 High Yield RNA Synthesis Kit (NEB E2040S), in a 60 μl total reaction for loxPsym+ mESCs and in a 30 ml total reaction for all other samples. Reactions were incubated at 37°C for 16 hours in a thermocycler with the lid set to 50°C. Reactions were treated with Turbo DNase (Thermo AM2238) to remove template DNA according to manufacturer’s instructions and RNA was extracted using Trizol LS reagent (Thermo 10296010). Briefly, each sample volume was normalized to 250 μl with water, 750 μl of Trizol LS reagent was added. Samples were mixed by pipetting and incubated at room temperature for 4 min. 200 ml of chloroform was added and samples were incubated at room temperature for 3 min. Samples were then spun at 12,000 × g for 15 min and the aqueous phase was transferred to a new tube. 1 ml of 5 μg/mL Glycogen was added (Invitrogen) per sample. Next, RNA was precipitated by adding 1 volume of isopropanol. Samples were mixed by inverting and incubated at −80°C for 1 hour and subsequently spun at 21,000 × g for 1 hour at 4°C. RNA pellet was washed with ice cold 80% ethanol and resuspended in 11.5 μl of H2O. Reverse transcription was performed using the SuperScript IV Reverse Transcriptase (Thermo 18090200) with 0.5 uL 100 μM RT primer that contains an 8-bp degenerate 3′ end (oSP1012). RNA was initially incubated with 0.5 μL 100 μM RT primer (oSP1012) and 1 μL 10 mM dNTP at 65°C for 5 min and cooled on ice. Enzyme, buffer, RNAse inhibitor and DTT were added, and reactions were incubated in a thermocycler at 23°C for 10 min, 50°C for 15 min and 80°C for 10 min, followed by a hold at 10°C.

For loxPsym+ mESC replicate 1, second strand synthesis for both top and bottom strands (fig. S1B) was performed in the same reaction with primers oSP1008, oSP1021, and oSP1013. For loxPsym+ mESC replicate 2, second strand synthesis for top and bottom strands were performed separately, one with oSP1008 and oSP1013 and another with oSP1021 and oSP1013. Four 50 μl PCR reactions were performed per sample with Q5 Polymerase (NEB M0492S) with the following cycling parameters: 98°C - 3 min; 4 cycles of 98°C - 20s, 65°C - 20s, 72°C - 30s; 72°C - 60s, hold at 4°C. Reactions corresponding to a particular sample and primer pair were pooled. For replicate 1, double-sided size selection (0.5X, 1.1X) was performed with Ampure XP beads (Beckman A63882) on 200 μl of sample and eluted in 50 μl of H2O. For replicate 2, double-sided size selection (0.5X, 1.1X) was performed on 100 μl of sample and eluted in 25 μl of H2O. From each sample, a second PCR was set up with indexing primers using 12 μl of the previous eluate as input. Two 50 μl PCR reactions were performed per sample with Q5 Polymerase (NEB M0492S) with real-time tracking using SYBR green dye with the following cycling parameters 98°C - 3 min; 4 cycles of 98°C - 15s, 65°C - 15s, 72°C - 30s until the curves reached saturation (14 to 16 cycles). Running these libraries on a D1000 ScreenTape (Agilent) revealed a smear of expected size but also some lower molecular weight products that may dominate the sequencing reaction. To address this, we created three equimolar pools: samples from replicate 1, top strand samples from replicate 2 and bottom strand samples from replicate 2. Pools were run on a 6% TBE PAGE gel and DNA between 400 bp and 1000 bp was excised and purified.

For both replicates of loxPsym+ K562, attB/P+ mESC and attB/P+ K562 samples, second strand synthesis was performed according to the replicate 2 protocol described above with minor modifications. Two 50 μl PCR 1 reactions were performed per sample, one corresponding to the top strand and one for the bottom strand. After bead clean-up, one 50 μl PCR 2 reaction was performed per sample, per strand. PCR2 was performed with the previous cycling parameters for a total of 16 to 18 cycles. Libraries were run on agarose gel and DNA between 400 bp and 1000 bp was excised and purified.

Library sequencing was performed on an Illumina NextSeq2000 P2 300 cycle kit. For loxPsym+ mESCs, the read lengths were: 106 read1, 10 index1, 10 index2, and 212 on read2. For all other samples, read lengths were: 124 read1, 6 index1, 10 index2, and 198 on read2.

All primer sequences are listed in table S1.

### Amplicon-seq library construction from bulk samples

As detailed in fig. S1, we employed two different strategies for amplicon-seq library construction: 2-primer (data in Fig. 3 and figs. S2C, S4, S5, and S6) and 4-primer (data in fig. S7 and all loxPsym+ K562, attB/P+ mESC and attB/P+ K562 experiments). The major difference between these two strategies is that either 2 or 4 primers corresponding to the capture sequences were used to generate the PCR product. In the case of the 2-primer strategy, the P5 Illumina sequencing adapter can only come from the primer that binds CS2 and the P7 adapter can only come from the primer that binds CS1. This precludes identification of recombined shuffle cassettes that contain the same capture sequence on both sides of the loxPsym site (fig. S1, C and D). In the 4-primer strategy, primers with P5 and P7 adapters that bind to both CS2 and CS1 are included in the PCR. Theoretically, this would enable us to detect recombined shuffle cassettes with the same capture-sequence on both sides. However, we do not detect these events even in the case of the 4-primer strategy, probably due to suppressive PCR (figs. S1, C and D, and S7, A and B).

For the 2-primer experiments, we used 250 ng of genomic DNA prepared using the Qiagen DNeasy kit as input. PCR1 (UMI addition) was performed in a 50 μl reaction with Q5 Polymerase (NEB M0492S), with 2.5 μL of each 10 μM primer (oSP1008 and oSP997) with the following cycling parameters: 98°C - 5 min; 4 cycles of 98°C - 20s, 62°C - 20s, 72°C - 30s; 72°C - 60s. PCRs were cleaned up using AmpureXP beads (1X) and eluted in 11 μl of H2O. PCR2 (sample indexing and sequencing adapter addition) was again performed with Q5 Polymerase in a 50 μl reaction using 10 μl of eluate from the previous step as template and 2.5 μL of each 10 μM sample indexing primer. Progression of PCR2 was monitored in real-time using SYBR green dye and reactions were stopped before saturation (usually 15 to 18 cycles). Cycling conditions for PCR2: 98°C - 5 min; 15 cycles of 98°C - 10s, 65°C - 10s, 72°C - 20s. Reactions were cleaned up with AmpureXP beads (1X) and eluted in 12 μl of H2O. Sample quality was confirmed on a TapeStation D1000 ScreenTape (Agilent), after which reactions were pooled. Sequencing was performed on an Illumina NextSeq2000 100 cycle kit with the following read lengths: 69 read1, 6 index1, 10 index2 and 53 on read2.

For the 4-primer experiments shown in fig. S7, 100 ng of genomic DNA prepared using the Qiagen DNeasy kit as used as input. For the 4-primer experiments involving loxPsym+ K562, attB/P+ mESC and attB/P+ K562, 250ng of genomic DNA prepared using the Qiagen DNeasy kit was used as input. PCR1 was performed with a mix of 4 primers (oSP1008, oSP997, oSP1021, and oSP1022) with 0.125 μl of each primer at 100 μM per 50 μl reaction. The rest of the protocol was identical to the 2-primer workflow described above.

All primer sequences are listed in table S1.

### Single-cell sorting and construction of amplicon-seq libraries

Cre reporter (pSP0767 pLV-Flox-BFP-dsRed) was cloned using pLV-flox-dsRed-GFP as a template using Gibson assembly (65). 200,000 cells were reverse transfected with 1 μg of Cre or Bxb1 recombinase and 200 ng of the reporter in a 6 well plate per reaction as described above. Two transfections were performed per recombinase. One set of cells per recombinase were treated with 20 μM Pifithrin-α 24 hours post-transfection for a total time of 48 hours. Cells were harvested at 72 hours post-transfection and FACS sorted on the activity of the Cre reporter at single-cell purity into gelatinized 96 well plates containing growth medium with or without Pifithrin-α. FACS data shown in fig. S8C was analyzed using FlowJo.

Clones were allowed to grow out for 9 days before cells were frozen for genomic DNA extraction in 96-well plates. Genomic DNA was extracted in the 96-well format using the Quick-DNA/RNA MagBead kit (Zymo Research R2130) according to the manufacturer’s instructions. Amplicon-seq libraries were constructed from 9.8 μl of template genomic DNA (estimated to be between 10 to 50 ng) per well using the 4-primer strategy described above. PCR1 was performed in a 20 μl Q5 Polymerase reaction with 0.05 μl of each primer at 100 μM. After 1X clean up with Ampure XP beads and elution in 11 μl of H2O, PCR2 was performed on 10 ml of eluate in a 25 μl Q5 Polymerase reaction with 1.25 μl of each indexing primer at 10 μM. After 18 cycles, 10 μl of each reaction was pooled and purified using a Zymo Research Clean and Concentrate kit. Sample was eluted in 100 μl of H2O, run on an agarose gel and band of the appropriate size was excised and purified (Zymo Research D4007). Libraries were run on an Illumina NextSeq 2000 200 cycle kit.

All primer sequences are listed in table S1.

### Preparation of cells and libraries for single-cell RNA sequencing

For loxPsym+ mESC scRNA-seq experiment described in fig. S15, 300,000 parental cells were transfected in 6-well plates as described above with 1 μg of Cre and 200 ng of the reporter per transfection. 10 individual wells were transfected. At 24 hours post transfection, 5 wells were treated with 20 μM Pifithrin-α for 48 hours total. At 72 hours post transfection, both Pifithrin-α treated and untreated cells were harvested and approximately 500,000 RFP positive cells were FACS sorted into a combined tube based on the activity of the Cre reporter. Untransfected parental cells were harvested, and 1 million cells were used as input in parallel with Cre-sorted cells for the protocol.

For the attB/P+ K562 and attB/P+ mESC “Lane 1” experiment described in Fig. 5, 200,000 parental cells were nucleofected (2 reactions) or transfected (3 reactions) with 1 μg of Bxb1 plasmid as described above. All cells were harvested at day 3 post-transfection and used as input for the protocol. For the “Lane 2” experiment, indicated bottlenecked populations were thawed and split once before being used as input in the protocol. For both Lanes, 500,000 cells from each population were mixed as indicated in Fig. 5A and were used for the subsequent steps.

After washing twice with cold 1X PBS (Gibco), 1 million cells per sample were resuspended in 400 μl of cold PBS. Cells were fixed with 1600 μl of cold 100% methanol, added dropwise with swirling. Cells were left on ice to fix with gentle swirling to mix every 5 min during the incubation. Cells were rehydrated with 4 mL of cold 1X PBS added slowly with gentle swirling of the tube. Cells were spun down and resuspended in 60 μl of PBS. For the loxPsym+ mESC experiment, approximately 60,000 cells and 380,000 cells in total were counted in the Cre and parental samples respectively using a Countess automated cell counter (Thermo) of which all and 100,000 cells in 18 μl of PBS were used for T7 IVT respectively. For the attB/P+ K562/mESC experiment, approximately 600,000 and 450,000 cells were counted of which, 100,000 cells per sample in 18 μl of PBS were used for T7 IVT. IVT reactions were set up in 30 μl total volume with the HiScribe T7 High Yield RNA Synthesis Kit (NEB E2040S) with 2 μl of each NTP, buffer, and enzyme. Reactions were incubated at 37°C for 1 hour in a thermocycler. Cells were immediately moved to ice and 20 μl of cold PBS was added to each sample. Approximately 40,000 cells were processed per lane of a 10X Genomics Single Cell 3′ HT with Feature Barcoding kit.

Transcriptome libraries were prepared as per the manufacturer’s protocol. To prepare libraries from T7 transcripts captured using CS1 and CS2, we started with the supernatant from the cleanup after cDNA amplification of the standard 10X Genomics feature barcoding protocol for the loxPsym+ mESC experiment. For the attB/P+ K562/mESC experiment, we spiked in two construct specific primers (oSP1059, oSP1060) at 0.5 μM into the cDNA amplification step. Two rounds of PCR were performed with primers specific to the shuffle cassette. In PCR1, oSP997 (CS1-TruSeq2), oSP1022 (CS2-TruSeq2) and oSP1061 (feature-cDNA primer F) primers were used (1.25 μl of 10 μM each) in a 50 μl Q5 polymerase reaction with 5 μl of template. Cycling conditions: 98°C - 45 s; cycles of 98°C - 20s, 60°C - 5s, 72°C - 5s; 72°C - 60s, 4°C - hold. 15 cycles of PCR1 were performed for the loxPsym+ mESC experiment and 12 cycles for the attB/P+ K562/mESC experiment. loxPsym+ mESC were cleaned up with AmpureXP beads (1X) and eluted in 15 μl of Qiagen buffer EB. attB/P+ K562/mESC reactions were cleaned up with AmpureXP beads (1X) and eluted in 30 ul of buffer EB. In PCR2, 5 μl of PCR1 eluate was used as input into either one or two (loxPsym+ mESC experiment) 50 μl Q5 Polymerase reactions per sample with sample index primers (1.25 μl of 10 μM each) that add Illumina adapters. Reaction progress was monitored using SYBR green and was stopped before saturation. Cycling conditions for the loxPsym+ mESC experiment: 98°C - 5 min; 9 cycles of 98°C - 10s, 65°C - 10s, 72°C - 20s; 72°C - 60s, 4°C -hold. Cycling conditions for the attB/P+ K562/mESC experiment: 98°C - 1 min; 8 cycles of 98°C - 20s, 63°C - 20s, 72°C - 1min; 72°C - 60s, 4°C - hold. After a clean-up with AmpureXP beads (1X), quality of the libraries was confirmed (prominent single-peak) by running them on a TapeStation D1000 ScreenTape (Agilent).

For the loxPsym+ mESC experiment, T7 libraries were sequenced on an Illumina NextSeq2000 100 cycle kit with the following read lengths: 28 read1, 6 index1, 8 index2 and 96 on read2. Transcriptome libraries were sequenced on two separate NextSeq2000 100 cycle runs, initially with 28 read1, 10 index1, 10 index2 and 90 on read2 and next with 28 read1, 6 index1, 8 index2 and 96 on read2.

For the attB/P+ K562/mESC experiment, T7 libraries were sequenced on an Illumina NextSeq2000 100 cycle kit with the following read lengths: 75 read1, 6 index1, 10 index2 and 47 on read2. Transcriptome libraries were sequenced on a NextSeq2000 P3 100 cycle kit with the following read lengths: 28 read1, 10 index1, 10 index2 and 90 on read2.

All primer sequences are listed in table S1.

### IVT-seq data analysis for insertion-site mapping in the parental population

Analysis pipeline was based on the pipeline published inref(37). Briefly, reads were demultiplexed using bcl2fastq (v2.20.0.422). Read1 contains the identity of the shuffle barcodes whereas read2 contains the associated genomic sequence. Reads were passed through a custom script (IVTextractBCs.py) to extract barcode sequences based on exact matches to the expected preceding and subsequent bases. The strand of each read (top-CS2 or bottom-CS1) was also assigned at this step. PiggyBac ITR sequences were trimmed from read2 using cutadapt (v2.5) with the following parameters: -cores=4–discard-untrimmed -e 0.2 -m 10 -a CCCTAGAAAGATA (66). Trimmed reads were mapped to either the mm10 or hg38 reference genome using bwa mem (v0.7.17) with -Y option (67). SAM files were sorted using sam-tools (v1.9) and filtered out reads that do not align to known PiggyBac insertions sites (TTAA) or align to several locations (contain XA:Z flag) using a custom script (align_filter.py) (68). Filtered SAM files were converted to BED format using the sam2bed tool in bedops (v2.4.35) (69).

For mESC samples, bedtools (v2.29.2) intersect was used with the -loj -wa -wb -filenames -sorted options to extract those alignments overlapping with a known variant between the BL6 and CAST alleles from the Sanger Mouse Genome database (40, 70). A custom python script (cleanup_sort_variantcall_update.py) was used to parse the CIGAR string of each alignment intersected BED file to assign each read to each of the following categories: BL6 (read overlaps with variant and sequence of alignment at that position matches the reference allele), CAST (read overlaps with variant and sequence of alignment at that position matches variant allele) and ‘no-Variant’ (read does not overlap with variant). Inconclusive alignments that contained some incongruence in allele assignment were discarded. The position of each alignment was determined by the genomic strand it mapped to. If strand is +, position is the end of the alignment and conversely, if strand is -, position is the start of alignment. For K562 samples, alignments were simply sorted and assigned to a position using a custom python script (cleanup_sort_variantcall_update_K562.py).

Next, another custom python script (ivt_clustered_groupcollapse_iterable.py) was used to collapse alignments into groups based on: 1) a shared chromosome and position and 2) a shared set of barcodes extracted from read1. Alignments at a given position were clustered together if their associated barcodes were within a Levenshtein distance of 6. Within each cluster, the most common values for barcode1, barcode2, strand of alignment to genome (+ or −) and shuffle cassette strand (top-CS2 or bottom-CS1) were assigned as the representative values for that cluster. For mESCs, each cluster was assigned to an allele based on the most common allele value within, without taking into account the number of no-Variant assignments within that cluster. Total read count (number of alignments per cluster), total read1 UMIs (coming from forward primer of second strand synthesis), total read2 UMIs (first 8 bp of genome coming from the degenerate 3′ end of the RT primer) and total number of unique alignment lengths per cluster were also determined.

To define the list of parental insertions, the outputs from the previous step from replicate 1 and replicate 2 were merged based on shared position, barcode and strand values. Within this merged dataset for mESCs, for each cluster of alignments, allele value was assigned based on the allele value in replicate 1 and replicate 2, without considering no-Variant assignments. In the case that replicate 1 and replicate 2 allele assignments were incongruent, allele for that cluster was assigned as ‘inconclusive’. Clusters with <6 reads and <3 unique lengths aligned in replicate 2 of the parental sample were filtered out. Clusters containing barcode combinations that did not map uniquely to a genomic location were then filtered out. Within this set, bonafide insertions were defined by: 1) a pair of read clusters whose alignment position differs by exactly 4 bp, 2) the first cluster in the pair maps to the - strand and the second cluster maps to the + strand, 3) the pair does not encode the same shuffle cassette strand (top-CS2 or bottom-CS1); and 4) the barcodes detected in each member of the pair are reverse complement of one another.

Within this set for mESCs, the allele value for each insertion was assigned as inconclusive in the case that the allele assignment for the pair of read clusters that make up the insertion were incongruent. In the case that one of the clusters were marked as no-Variant, the allele of the other cluster in the pair was considered the allele of that insertion. Insertions were merged with amplicon-seq data from the parental cells based on shared barcode pairs. Only those insertions whose barcodes could be detected in the amplicon-seq data from the parental cells were kept. In some cases, there were a small number of remaining sites that had more than one pair of barcodes called at that site. By manual observation, these seemed to arise from a clustering artifact and ought to have been collapsed into one cluster. We arbitrarily chose the row that had the higher value in the number of unique lengths aligned in rep2 on the left side of the insertion at these positions for the final set.

To generate the visualization in Fig. 2A, alignments were visualized in IGV 2.16.1 (71) and BED files containing insertions assigned to each allele were loaded separately as tracks. The visualizations shown in Fig. 2D and fig. S3 were generated using the ChIPseeker package in R (72). Other plots were made using a combination of matplotlib (3.8.1) and seaborn (0.13.0) libraries in Python.

List of parental shuffle cassette insertion positions in loxPsym+ mESCs are in table S2. List of parental shuffle cassette insertion positions in attB/P+ mESCs are in table S5. List of parental shuffle cassette insertion positions in loxPsym+ K562s are in table S6. List of parental shuffle cassette insertion positions in attB/P+ K562s are in table S7.

### Amplicon-seq analysis and rearrangement calling

Reads were demultiplexed using bcl2fastq (v2.20.0.422). Reads were passed through a custom script (read_extract_iterable.py or read_extract_iterable_4primers.py or amplicon_BCExtract_20240906.py) to extract barcode sequences and UMIs based on matches to the expected preceding and subsequent bases. In the case of 4-primer amplicon-seq, the strand of each read (top-CS2 or bottom-CS1) was also assigned at this step. The identity of the recombination site (loxP, attB, attP, attL or attR) was determined from the combination of bases specific to each site expected to be detected in read 1 and read 2. Total number of reads per barcode pair was taken as the readcount and the total number of unique UMIs detected was taken as the UMI count (df_group.py or df_group_4primers.py). In 4-primer amplicon-seq, the same shuffle cassette can result in two distinct amplicons. We collapsed reads coming from the same shuffle cassette based on the shared set of barcodes and summed up the read counts and UMI counts. Those barcode pairs where we did not detect both types of amplicons were discarded. In all cases, the barcode closest to CS2 was named barcode1 and the barcode closest to CS1 in the shuffle cassette was named barcode2. Read and UMI counts were normalized for sequencing depth. For the loxPsym+ mESC parental barcode set, normalized read and UMI counts were averaged across 4 replicates and used for defining the bonafide set of insertions as detailed in the section above. For the remaining parental samples, normalized read and UMI counts were averaged across 2 replicates and used for defining the bonafide set of insertions as detailed in the section above.

For the plot in fig. S7B, we looked for amplicons with shared barcode pairs that contained the same capture sequence. To eliminate confounding through errors in PCR or sequencing, we restricted our search to those amplicons containing barcodes which were both found in the bonafide list of parental insertions. We also eliminated those amplicons that contained the same barcode (that is, within Levenshtein distance of 6) on both sides of the loxPsym site. CS1-CS1 and CS2-CS2 amplicons were not readily detected in our data, presumably due to suppressive PCR (figs. S1 and S7B).

For the amplicon-seq data generated from single-cell sorted clones (fig. S8), wells with fewer than 100k reads were discarded. Barcode sequences were extracted and read/UMI counts per barcode pair were determined as above. The set of barcodes associated with each well were determined as the barcode pairs whose readcounts were >1 standard deviation above the mean readcount for barcode pairs detected in that well using the zscore function in the scipy.stats library. Analysis was then restricted to those barcode pairs that were present in the bonafide parental insertion list (*n* = 5088). Barcode pair count per well was normalized for the number of clones observed in that well by eye.

Rearranged barcodes were identified by comparing the set of identified barcode pairs in the dataset to the bonafide list of parental insertions. Both barcodes in the rearranged pair were required to be in the bonafide list but were not found together in the parental cells. To remove any artifacts caused by PCR chimeras or errors, each rearranged barcode pair was required to be present at ≥2 UMI. The nature of each rearrangement denoted by a rearranged barcode pair was inferred based on the position and orientation of the parental insertion sites (Figs. 1 and S1). We first determined that inter-homolog translocations were rare (recombination between shuffle cassettes on the same chromosome but assigned to different alleles). Therefore, we parsimoniously identified deletions as those rearranged barcode pairs between shuffle cassettes on the same chromosome that were inserted in the same orientation. In a similar manner, inversions were identified as those rearranged barcode pairs between shuffle cassettes on the same chromosome that were inserted in the opposite orientation. The size of the rearrangement was calculated as the difference between the position of the two original insertion sites. Translocations were those rearranged barcode pairs found between shuffle cassettes on different chromosomes.

Deletions could be further classified as coming from the genomic copy or the extrachromosomal circle based on the barcodes that were detected (Figs. 1 and 2D). For example, let us consider the case of a deletion between two shuffle-insertions X and Y at pos N and pos N+100 on a chromo-some, with ‘top-CS2’ orientation (CS2 found closest to the left of the chromosome). The ecDNA would contain the CS2 barcode of insertion Y and and CS1 barcode of insertion X and the genomic copy would contain the CS2 barcode of insertion X and and CS1 barcode of insertion Y. In the case of deletions between two ‘bottom-CS1’ insertions with (CS1 found closest to the left of the chromosome), the opposite would be true.

Translocations could also be further classified as balanced and unbalanced. Barcode pairs corresponding to unbalanced translocations could further be assigned as leading to either an acentric or dicentric chromosome (Fig. 4G). The relative position of each translocation breakpoint to the centromere was determined. Based on the orientation of the insertion (top or bottom strand), each barcode was assigned to either be centromere proximal or distal. If both barcodes corresponding to the detected translocation were centromere proximal, the barcode combination was classified as having originated from a dicentric chromosome. If both barcodes were centromere distal, the translocation was classified as an acentric. In other cases the translocation was a balanced translocation.

All Circos plots were made using the pyCircos library (73). The remaining plots were made using a combination of matplotlib (3.8.1) and seaborn (0.13.0) libraries in Python.

All rearranged barcodes detected in 2-primer bulk amplicon-seq at day 3 post Cre treatment in loxPsym+ mESCs (data used in Figs. 3 and 5, and figs. S4 to S6 and S11) are in table S3. All rearranged barcodes detected in both technical replicates of 2-primer bulk amplicon-seq at day 3 post Cre treatment in loxPsym+ mESCs (data used in Figs. 3 and 5 and figs. S4 to S6 and S11) are in table S4.

All rearranged barcodes detected in 4-primer bulk amplicon-seq at days 3, 5, and 7, post Bxb1 treatment in attB/P+ mESCs (data used in Fig. 4 and figs. S11 to S14) are in table S8. All rearranged barcodes detected in 4-primer bulk amplicon-seq at days 3, 6 and bottlenecked populations, post Cre treatment in loxPsym+ K562s (data used in Fig. 4 and figs. S11 to S14) are in table S9. All rearranged barcodes detected in 4-primer bulk amplicon-seq at days 3, 6 and bottlenecked populations, post Bxb1 treatment in attB/P+ K562s (data used in Fig. 4 and figs. S11 to S14) are in table S10.

### Rearrangement call validation using IVT-seq data

IVT-seq libraries were constructed using the same genomic DNA samples used to prepare amplicon-seq libraries from Cre-transfected samples. The data was pushed through the same analysis pipeline as described above for IVT data from parental cells, until the collapsing of alignments into groups based on their shared barcodes and positions. For each rearrangement detected in the amplicon-seq data, we asked whether there was at least one transcript detected in the IVT-seq data from that sample that supported the rearrangement call (Fig. 3D). The fraction of rearrangements supported in each IVT replicate from each sample were plotted using matplotlib (3.8.1) library in Python.

### Single-cell data analysis

#### Initial pre-processing and quality filtering

10X Genomics 3′ gene expression (transcriptome) libraries were processed using cellranger-6.0.1 count function (with reference refdatacellranger-mm10–3.0.0 for mESC cells [loxPsym+ mESC experiment 1 and attB/P+ mESC/K562 experiment lane 1] and refdata-gex-GRCh38–2020-A for K562 cells [attB/P+ mESC/K562 experiment lane 1 and 2]). Resulting raw count matrices were converted to a Seurat (v4.3.1) (74) object using functions Read10X and CreateSeuratObject (options: min.cells=3, min.features=50). The mitochondrial fraction was computed and cell barcodes with >1500 and >1000 transcriptome UMI/cell (K562 and mESC respectively) and with 2–8% and 1.3–6% mitochondrial fraction (K562 and mESC respectively) (fig. S16A) were retained. For loxPsym+ mESC experiment 1, cell barcodes with >1000 transcriptome UMI/cell and with 1–12% mitochondrial fraction (fig. S15) were retained.

Scrublet 0.2.3 (75) was run on filtered cells, and cells with doublet score <0.4 were retained, leaving 12460 and 8499 (K562 and mESC respectively, lane 1) and 20800 (K562, lane 2) high-quality cells for downstream analysis.

For the lane 1 mESC+K562 barnyard experiment, the following criteria were further used to unambiguously assign species identity to cells: cell barcodes passing each separate species singlet processing thresholds were retained, and any cell barcodes removed as a putative scrublet-called doublet in one species but not the other were further marked as doublets (e.g., cell called as singlet in K562 mapping but flagged as a doublet in mESC mapping). Total transcriptome UMIs mapping to both species from all these cell barcodes were then inspected, showing clear separation between singlet and doublet. Initial assignment from the single species threshold was finely refined to stringently select cells as doublets if transcriptome UMI ratios were not sufficiently separate (threshold selected by inspection): mm10/hg38 <1.42 or hg38/mm10<3, leaving a final set of 11117 K562 and 7307 mESC cells (1172 assigned putative species doublets).

All samples displayed a cleanly separated cell population in the total transcriptome vs. mitochondrial fraction plane including cells with high confidence rearrangements (fig. S16A), indicating good cellularity despite fixation and IVT treatment to generate the T7-BC prior to emulsion and library generation.

### Generating T7-shuffle BC count matrix

To obtain the BC-by-cell-count matrix, we first modified the fastq files to separate the cell barcode and shuffle barcodes in two distinct files (original read structure–read 1: first 28 cycles [cell barcode]-umi, remaining cycles 29 to 75 to read [capture sequence]-BC-[att site]; read 2: cycles 1 to 47 cycles to [read capture sequence]-BC-[att site]). Modified fastq content–read 1: 28 cyclces [cell barcode]-umi, read 2: cycles 1 to 47 cycles to [read capture sequence]-BC-[att site] followed by original cycles 29 to 75 from original read 1. The merge was generated using seqtk version 1.4 and the following script (appending line number to join and retaining only desired information in final fastq (seqtk trimfq -b 28 file_R1_001.fastq.gz | join <(zcat file_R2_001.fastq.gz | nl) <(cat - | nl) | awk -F ‘ ‘ ‘{if ($2 ~ /^@VH00979/) {print $4” “$5;} else {print $2$3;}}’ | gzip > file_w_47bpR1R2transfer_R2_001.fastq.gz).

T7-BC sequencing data was then processed using cellranger-6.0.1 count to perform error correction on the cell barcodes. The unmapped reads with error-corrected cell barcodes were selected from the sorted BAM file output, and barcode sequences were extracted for each read from the shuffle cassette by looking for matches for constant surrounding sequences. From the read 1 portion (searching in cyles 48 to 95 appended to read 2 as described above): attP CS2 if TGAGC(.{20})GTGG, attB CS2 if TGAGC(.{20})GGCC, attP CS1 if AAAGC(.{20}) TGGG, attB CS1 if AAAGC(.{20})CCGG. From the original read 2 (searching in cycles 15 to 47): attB CS1 if AAAGC(.{20})CCGG, attP CS1 if AAAGC(.{20})TGGG, attP CS2 if AAAGC(.{20}) TGGG, attB CS2 if TGAGC(.{20})GGCC. The resulting two (barcode)-(capture sequence)-(recombinase site) combinations were stored and joined to the error-corrected cell barcode and umi from the read. Reads counts and total set of UMIs for all cellBC/BC1/BC2/capture/recombinase site combinations were then tallied, discarding likely chimeric UMIs (taken to be UMIs for which the proportion of reads associated to a given BC1/BC2/capture-orientation all other BC1/BC2/capture/recombinase site in the specified cell barcode falls below 0.2). The number of error-corrected UMIs for a given BC1/BC2/capture/recombinase site was then taken as the number of connected components in a graph created by connecting all UMIs associated with that combination with a Hamming distance ≤1. To filter out spurious molecules (sequencing errors or PCR chimeras, only cell barcode BC1/BC2 combinations with reads/UMI ≥15 and ≥9 for Lane 1 and Lane 2, respectively, were retained commensurate with the level of sequencing saturation in both libraries).

All cells detected in Lane 1 of attB/P+ K562/mESC 10X experiment with associated characteristics (data used in Figs. 5 and figs. S16 to S20) are in table S11. All cells detected in Lane 2 of attB/P+ K562 10X experiment with associated characteristics (data used in Figs. 5, and figs. S18 to S21) are in table S14.

### De novo identification of clonotypes

Similarly to previous works (63, 76, 77), we leveraged high MOI (many T7-BC pairs per cell) and clonal nature of the population to identify clonotypes (defined as the full set of BC pairs within a clone) directly from the single-cell data T7-BC data, working under the assumption that co-detection of T7-BC pairs should only happen from barcodes within the same clone given the high complexity of starting libraries.

We first summed UMI counts in the same cell with the same BC1/BC2 pair but captured from different capture sequences. To avoid improper tally of re-arranged barcodes, only the BC associated with CS1 was used for clonotype identification. We then subsetted the T7-BC UMI counts to those originating from the set of quality filtered cells as described above and retained barcode pairs with ≥ 3 UMI per barcode per cell. To further remove cells and barcode pairs with little signal for the purpose of de novo clonotype identification, we removed any cells with <11 total T7-BC UMIs (after the ≥3 UMI/BC per cell thresholding) and T7-BC pairs with <11 total UMI across all cells. We then constructed a count matrix and used Seurat v4.3.1 to perform dimensional reduction (NormalizeData with normalization.method=“RC”,scale.factor=10000, FindVariableFeatures with selection method= “vst”, nfeatures=length(T7_BC), RunPCA on the top 100 PCs with identified variable features, FindNeighbors with k.param=10, and FindClusters at resolution=1). The resulting communities in T7-BC space were used to identify the putative set of shuffleBC cassettes per clonotype.

To do so, T7-BC representation across all cells assigned from clustering in the BC space was calculated as the proportion of cells in the cluster with the barcode pair detected. These proportions were then rank ordered, and a heuristic threshold was used to demarcate barcode pairs associated with a clone: the threshold was set as the fold-change in representation from rank n BC pair to rank n+1 barcode pair became higher than 2 (Lane 1) and 1.5 (Lane 2) or 0.075 (Lane 1) and 0.1 (Lane2) whichever was highest, empirically selected as a reliable marker of the inflection point in the distribution. The resulting set of putative clonotypes were further filtered by retaining only clonotypes for which the maximum detection fraction (from the top T7-BC pair for that clonotype) was above 0.45 (Lane 1) and 0.55 (Lane 2), and in which >1 T7-BC pairs were detected. In addition, subsetted T7-BC count matrix for each putative clonotype (with only assigned cells and contained T7-BCs) was inspected, and any clonotype corresponding to a clear doublet (split in the matrix in two blocks) was not retained for downstream analysis.

As a final quality control step, because this procedure tends to redundantly create multiple clusters for the same clonotype (depending on the resolution parameter), we computed the Jaccard index (on the set of T7-BCs) for each identified clonotype pair. For Lane 1, the distribution of Jaccard indices was strongly bimodal, with the majority of pairs displaying 0 overlap, with a small minority with Jaccard index of >0.5, suggesting identical underlying clonotypes. A graph was created with nodes corresponding to putative clonotypes and connected if their Jaccard index was >0.5. The union of the T7-BC from the connected clusters (mostly singletons) was then taken as the clonotypes. All in all, this led to 91 high confidence de novo clonotypes from round 1 (see below for round 2), with a mean MOI of 31.4. For Lane 2, similar analysis Jaccard similarity revealed a set of clonotype-assigned barcodes that were broadly shared across nearly all clonotypes. Given the clonal representation in the population (two clones with >2000 cells each, making up nearly 45% of all cells in the dataset), we suspected that the shared barcodes originated from these highly represented clones. Calculating the mean UMI per BC per putative cluster in the T7-BC space indeed confirmed that the highly represented clusters were detected at a much higher proportion (mean 4 UMI/cell compared to 1.2 UMI/cell for other clusters) in one of the large clusters. To avoid these barcodes from large clones to be spuriously included in our clonotype calls, Lane 2 analysis was repeated after excluding cells and barcodes assigned to these two large clones in the first pass. Procedure for the large clone excluded set for Lane 2 then proceeded as before, leading to 53 high confidence clones (including the two large clones), with a mean MOI of 26.5.

### Iterative round of assignment and mapping to identify additional clonotypes

In order to comprehensively identify clonotypes, we performed an iterative approach whereby cells were first assigned to clonotypes (see below), as described using the round 1 clonotypes described above. Any assignable cell was then removed from the de novo pipeline described previously, and the process repeated. Doing so generated an additional set of 50 (final 141 total, Lane 1) and one (final 53 total, Lane 2) high confidence clonotypes.

List of clonotypes and associated barcodes identified from Lane 1 and Lane 2 of attB/P+ K562/mESCs single-cell RNA sequencing are in table S17.

### Single-cell assignment of cells to clonotypes

To obtain a more sensitive assignment of cells to clonotypes (in contrast to the clustering in a dimensionally reduced barcode space used for de novo clonotype calling as described above), we compared all the T7-BC detected (considering only events with ≥ 2 UMI per barcodes pairs per cell, after summing pairs captured from both CS1 and CS2, see barnyard and clonotype precision/recall analysis below) in all cells to the set of high-confidence clonotypes. In order not to bias against re-arranged cells for the purpose of assignment to clones, only the CS1 barcode was considered for this analysis or each cell-clonotype pair, the fraction of cell-detected barcodes belonging to the top clonotype (precision) was recorded, in addition to the fraction barcodes from the clonotype recovered in the cell (recall). Across all cells, the clonotype with the highest precision (similar result if selecting on recall) was retained as the best candidate (top_precision, top_recall). The resulting distribution of top scoring clonotype precision/recall assignments displayed enrichment in high precision values with a range of recall. Cells in that plane were considered assignable to a clone with high confidence if top_recall>0.1 and top_precision>0.75. To further remove the possibility of doublets, any resulting assigned cells with second-top assigned clones showing recall > 0.1 were removed from the set of high-confidence assignments. For Lane 2, as a result of the highly represented clonotypes ‘emitting’ shuffleBC IVT transcripts at substantial level in the ambient mixture that were captured in other droplets (see discussion in clonotype reconstruction section), a number of cell assignments to less well represented clonotypes were initially called as ‘low purity’. To circumvent this issue, we repeated the assignment to clonotypes but excluding barcodes originating from these large clonotypes. Any cell that was initially assigned as low purity (assignment with large clonotype barcodes) and subsequently as high purity (assignment without large clonotype barcodes) was putatively retained as a high confidence assignment. 2797/19987 cells with at least one BC with ≥ 2 UMI detected fell in that category, underscoring an opportunity for optimization in future iterations to wash cells and remove non-cell associated IVT products prior to encapsulation.

In the end, for Lane 1, 65.2% (11252/17266) of cells with at least one BC pair with ≥ 2 UMI) of cells were assignable to a clonotype. Of the remaining cells, 3526 displayed low capture (recall < 0.1) either as a result of missed clonotypes from our reconstruction procedure or from low levels of IVT for the T7-BC generation. The remaining cells, where the set of detected T7-BCs are not predominantly from a single clonotype, either originated from droplets encapsulating doublets or large quantities of ambient RNA. From the original set of 143 clonotypes, 128 had more than 10 cells assigned to them, and were considered for the precision/recall analysis. Further, 5 clonotypes had 0 highly confidently assigned cells, suggesting mixed or otherwise low quality clonotypes in our de novo reconstruction procedure, of note, these were ‘round 2’ clonotypes that tend to have low MOI, rendering assignment more challenging.

For Lane 2,73.7% (14727/19987) of cells with at least one BC pair with ≥ 2 UMI) of cells were assignable to a clonotype. Of the remaining cells, 2156 displayed low capture (recall < 0.1) and the rest low purity. 52/53 clonotypes had >10 cells assigned at high confidence.

Assignment of cells to clonotypes based on the set of T7-derived barcodes detected within them for Lane 1 of attB/P+ K562/mESC 10X experiment are in table S18. Assignment of cells to clonotypes based on the set of T7-derived barcodes detected within them for Lane 2 of attB/P+ K562 10X experiment are in table S19.

### Precision-recall analysis for T7-shuffle BC detection

In order to assess which thresholds to use to identify high-confidence detections of rearrangement events, we performed cross-detection analysis in the barnyard experiment (Lane 1, Fig. 5A).

First, at a coarse-level, we assessed the fraction of shuffleBC known to be present in the mESC population (bulk amplicon sequencing, *n* = 3058 BCs) that were captured in K562 cells in our scRNA-seq data (and vice-versa, *n* = 11166 BCs by bulk amplicon sequencing of K562 population). Specifically, only shuffle BC in the bulk amplicon sequencing set were used for the analysis (corresponding to 97.8% of scRNA-seq detected UMIs). Then, cells that were unambiguously assignable to a single species (see processing of scRNA-seq above) were retained. For varying UMI threshold value (1 to 10), the number of UMIs from the K562-BCs and mESC-BCs sets detected in these species-singleton cells (either K562 or mESC) was computed. Barnyard plots (Fig. 5B and fig. S17A) highlighted a near complete absence of cross-detection for detection events at ≥2 UMI (>98% and >99% UMIs detected in the cognate species at a threshold of ≥2 and ≥3 respectively, fig. S17B).

Since the cross-species analysis above still calls a spurious detection valid in nearly half of the cases (e.g., shuffleBC emitted in ambient medium from an mESC clonotype and detected in an mESC cell from another clonotype), we sought a more stringent performance assessment. To do so, we considered high confidence assigned cells to major clonotypes (>10 cells assigned) and determined precision and recall values for varying UMI thresholds. At each UMI threshold, the precision and recall values were averaged over all high-confidence cell-toclonotype assignments. The curves displayed a sharp increase at 2 UMI (1 UMI mean precision 0.40 to 2 UMI mean precision of 0.92, fig. S17C) with modest decrease in recall (0.55 to 0.33), providing solid empirical ground for taking the threshold of 2 UMI as associated with a <8% FDR. We note that the cross-detection performance is a function of the statistical distribution of shuffleBC ‘emitter’ source, as exemplified with the added background resulting from the highly represented clonotypes in the Lane 2 data.

### Single-cell expression analysis

The cell × gene count matrix was normalized by the library size of each cell using Scanpy (78) after removing genes that were expressed in <10 cells. Fold-changes in normalized expression were compared between the rearranged and nonrearranged groups of cells within the same clone, and these fold-changes were plotted against genomic coordinates using gene annotations from Gencode v38 (79) (Fig. 5F). The statistical significance of the reduction in expression due to the deletion was assessed using a one-sided Wilcoxon rank sum test. This test was also performed for each of the three genes in the deleted region for various sample sizes of rearranged cells. Cells were randomly sampled without replacement from the full set of cells with the confidently detected deletion, and this was repeated for 100 trials per sample size to get an estimate of the variability in statistical significance as it relates to sample size. The fold-changes for the same analysis were plotted and calculated as before (fig. S21C).

### Rearrangement calling and visualization from single-cell data

From the set of T7-BCs identified in the single-cell data, rearrangements were called as described above for bulk amplicon-seq. The rearranged BC pairs identified in the dataset were filtered to those present at ≥2 UMI, in the cells of the correct species in Lane 1, to those in cells assigned to a clonotype and finally to those rearrangements consistent with the clone of the cell they were detected in (fig. S19, A and B). The subsequent analysis focused on the validated set of rearrangements. Circos plots were made using the pyCircos library. Other plots were made using a combination of the matplotlib (3.8.1) and seaborn (0.13.0) libraries in python.

All rearranged barcodes detected in Lane 1 of attB/P+ K562/mESC 10X experiment (data used in Fig. 5 and figs. S16 and S18 to S20) table S12. Filtered rearranged barcodes detected in Lane 1 of attB/P+ K562/mESC 10X experiment at ≥2 UMI, and congruent with the assigned clone of that cell (data used in Fig. 5 and figs. S16, S19, and S20) are in table S13.

All rearranged barcodes detected in Lane 2 of attB/P+ K562 10X experiment (data used in Fig. 5 and figs S16, and S18 to S21) are in table S15. Filtered rearranged barcodes detected in Lane 2 of attB/P+ K562 10X experiment at ≥2 UMI, and congruent with the assigned clone of that cell (data used in Fig. 5 and fig. S16, and S18 to S21) are in table S16.

## Supplementary Material

Supplementary Materials

Supplementary Tables

MDAR Reproducibility Checklist

## Data Availability

Analysis pipelines and plasmid sequences can be accessed at the repository under reference (80). Raw sequencing data and processed data files are deposited to the Gene Expression Omnibus (GEO) under GSE282636. Details of all sequencing libraries are in table S20.
