# Supplementary material for: Multiplex generation and single-cell analysis of structural variants in mammalian genomes
Source: Science. Author manuscript; Available in PMC 2025 Mar 24. (PMC11931979; doi:10.1126/science.ado5978)
Supplement: Supplementary Materials [file NIHMS2066299-supplement-Supplementary_Materials.pdf]

Science AAAS

Supplementary Materials for

Multiplex generation and single-cell analysis of structural variants in mammalian genomes

Sudarshan Pinglay *et al*.

Corresponding authors: Sudarshan Pinglay, pinglay@uw.edu; Jay Shendure, shendure@uw.edu

Science **387**, eado5978 (2025)
DOI: 10.1126/science.ado5978

The PDF file includes:

Materials and Methods
Figs. S1 to S21
References

Other Supplementary Material for this manuscript includes the following:

Tables S1 to S20
MDAR Reproducibility Checklist

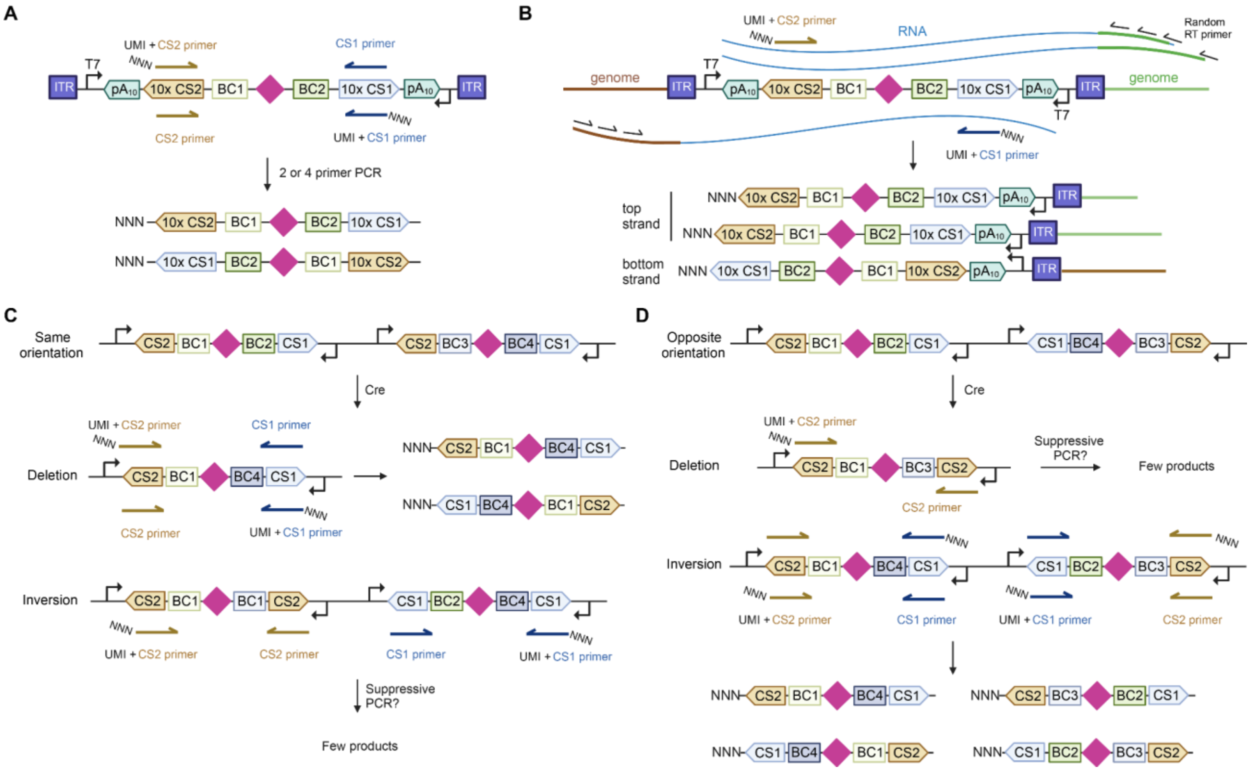

Fig. S1. Schematic of sequencing library construction strategies for amplicon-seq and IVT-seq. A) Schematic of the shuffle cassette bearing the loxPsym site (diamond) flanked by two barcodes (BC1, BC2). Two 10x Genomics capture sequences (CS1, CS2) serve as binding sites for PCR primers for sequencing library construction. Only amplicons generated using one unique molecular identifier (UMI)-containing primer and one non-UMI primer can cluster and be successfully sequenced on an Illumina flow cell due to the sequencing adapters they encode. B) After IVT, transcripts are generated from both the top and bottom strand T7 promoters and are expected to contain both BC1 and BC2 as well as adjacent genomic sequences from one side of the integrated shuffle cassette. Reverse transcription (RT) is performed with a primer containing 8 random bases at its 3' end. PCR is performed with a UMI-containing primer and one primer annealing to the constant sequence in the RT primer to yield the final sequencing library. C) and D) Recombination between two insertions *in cis* can lead to an inversion or deletion with shuffle cassettes containing either the same or different capture sequences. Theoretically, PCR products from a cassette with the same CS should amplify and cluster on an Illumina flowcell when libraries are generated using all 4 primers. However, empirically we find that these products are not readily detected, probably due to suppressive PCR (*41*, *42*) (see Fig. S7B).

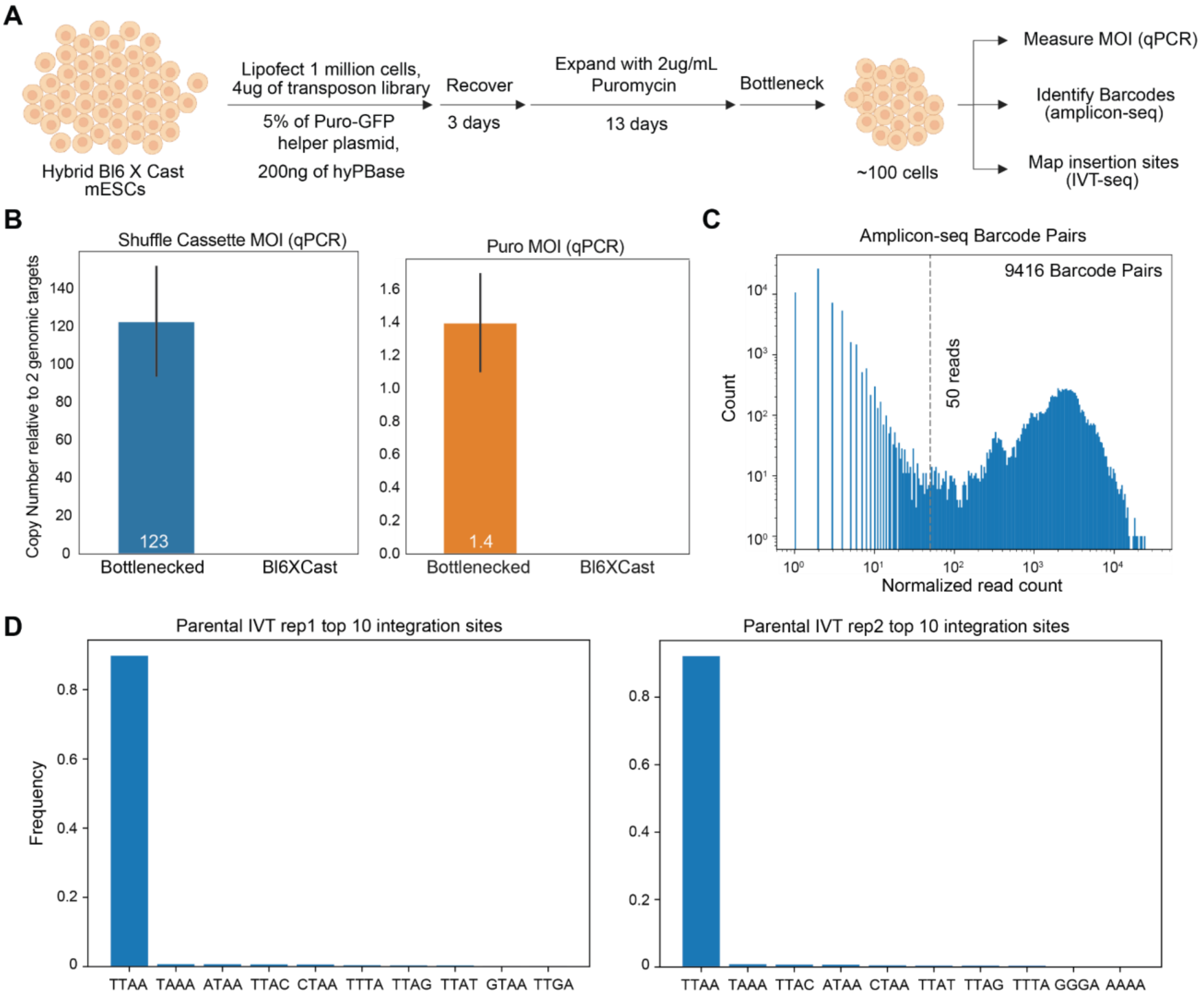

Fig. S2. Integration and characterization of shuffle cassette library into mESCs. A) Schematic of experiment to integrate shuffle cassettes to the genomes of mESCs at a high multiplicity of infection (MOI) by co-transfection with a small percentage of a helper plasmid containing the puromycin resistance gene (*39*). **B)** Copy number of shuffle cassettes and the puromycin resistance gene were estimated in the bottlenecked population via quantitative PCR (qPCR) relative to two genomic targets (Trfc, Tert). The height of the bar represents the mean and the error bars indicate the standard deviation of the copy number measured relative to the two genomic targets. **C)** Histogram of read count for each barcode pair detected in amplicon-seq data normalized to sequencing depth across 4 technical replicates. **D)** Frequency of the first 4 bp of the genomic sequence detected in IVT-seq reads in technical replicate 1 and 2 from parental cells. TTAA is the expected sequence given our use of the PiggyBac transposon.

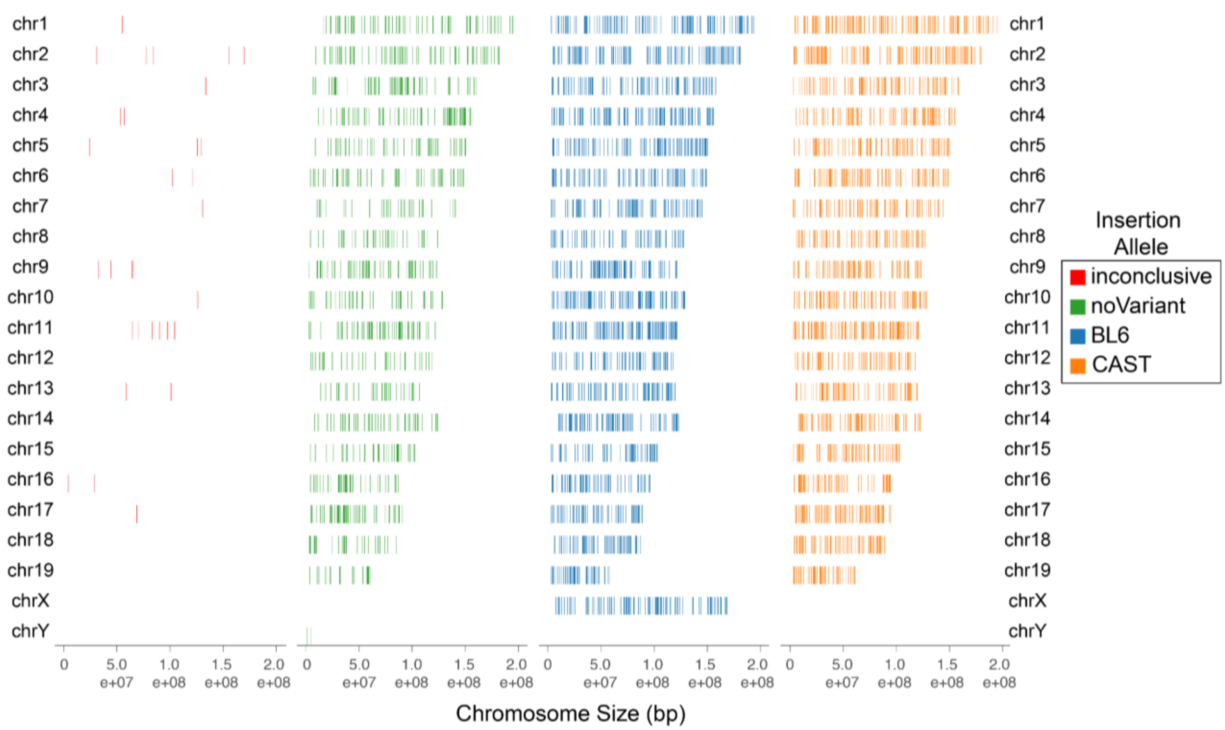

Fig. S3. Allele-specific insertion sites across all chromosomes. Insertion sites across all chromosomes for shuffle cassettes whose genomic coordinates were mapped with high confidence, colored by allele. Inconclusive indicates that there is conflicting evidence for the insertion allele, while noVariant denotes those insertions that were un-assigned due to a lack of reads that overlap with a known variant between the BL6 and CAST genomes.

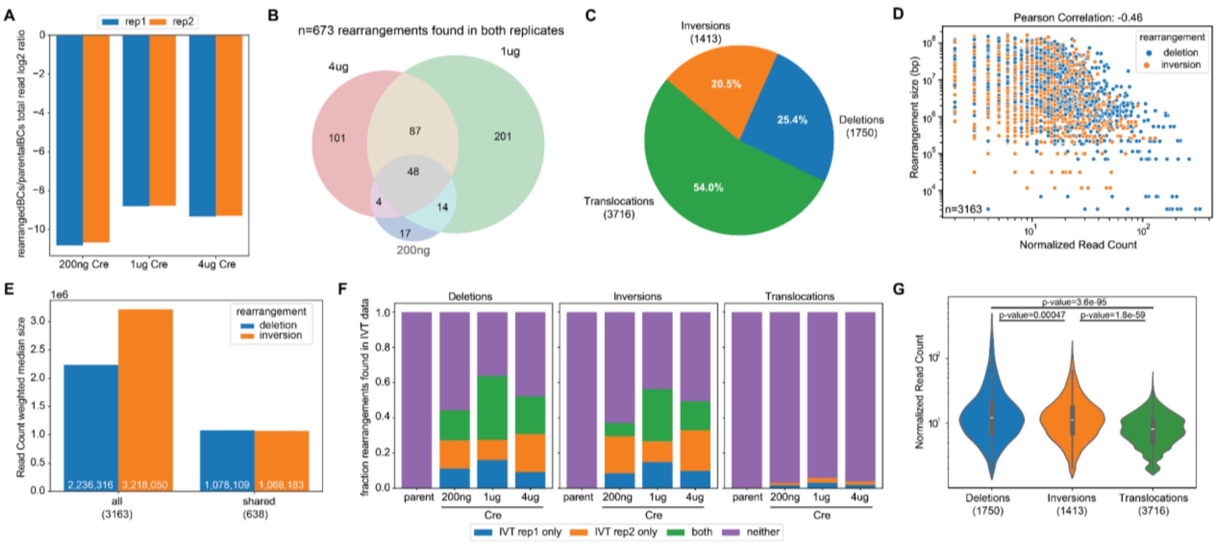

Fig. S4. Characteristics of the complete set of rearrangements detected in bulk by amplicon-seq at 72h post-Cre transfection. A) Log2 ratio of total reads that contain rearranged barcode (BC) pairs to the total number of reads that contain parental BC pairs in technical replicates of each Cre transfection sample. **B)** Venn diagram depicting the overlapping relationships between Cre transfection samples for the subset of SVs that are detected in both technical replicates of each sample. **C)** Pie chart depicting the distribution of SV type for all rearrangements detected at 72h. **D)** Scatter plot of rearrangement size (*y*-axis) vs. normalized read count (*X*-axis) for deletions and inversions detected at day 3. Pearson correlation is calculated between the log10 values of the two metrics. **E)** Median size of inversions and deletions, weighted by their read count, for both the complete set of rearrangements (left) and those shared between technical replicates for a condition (right). **F)** Similar to lower part of **Fig. 3D**, the bar plot shows the proportion of each SV type (from the complete set of rearrangements at 72h) that is supported by at least one read in the IVT-seq data. **G)** Violin plots depicting the distribution of read counts for deletions, inversions and translocations for the complete set of rearrangements detected at day 3. Inset within each violin plot is a box plot of the distribution with the median value depicted as a white line, the length of the box depicting the interquartile range and the whiskers depicting the extent of the distribution. P-values are calculated using the non-parametric Mann-Whitney U test.

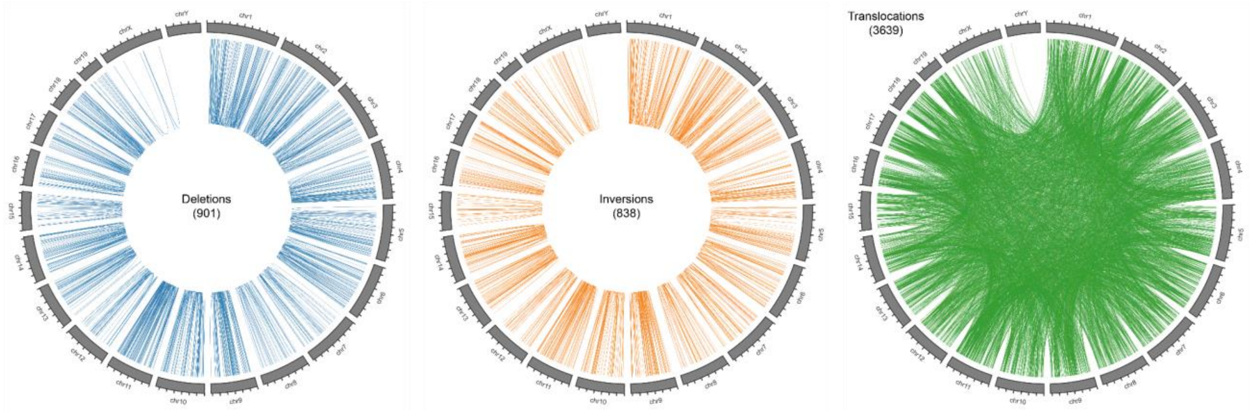

Fig. S5. Circos plots of all rearrangements detected at 72h post-Cre transfection. Depicted rearrangements are from across all samples, including those that are not shared between technical replicates.

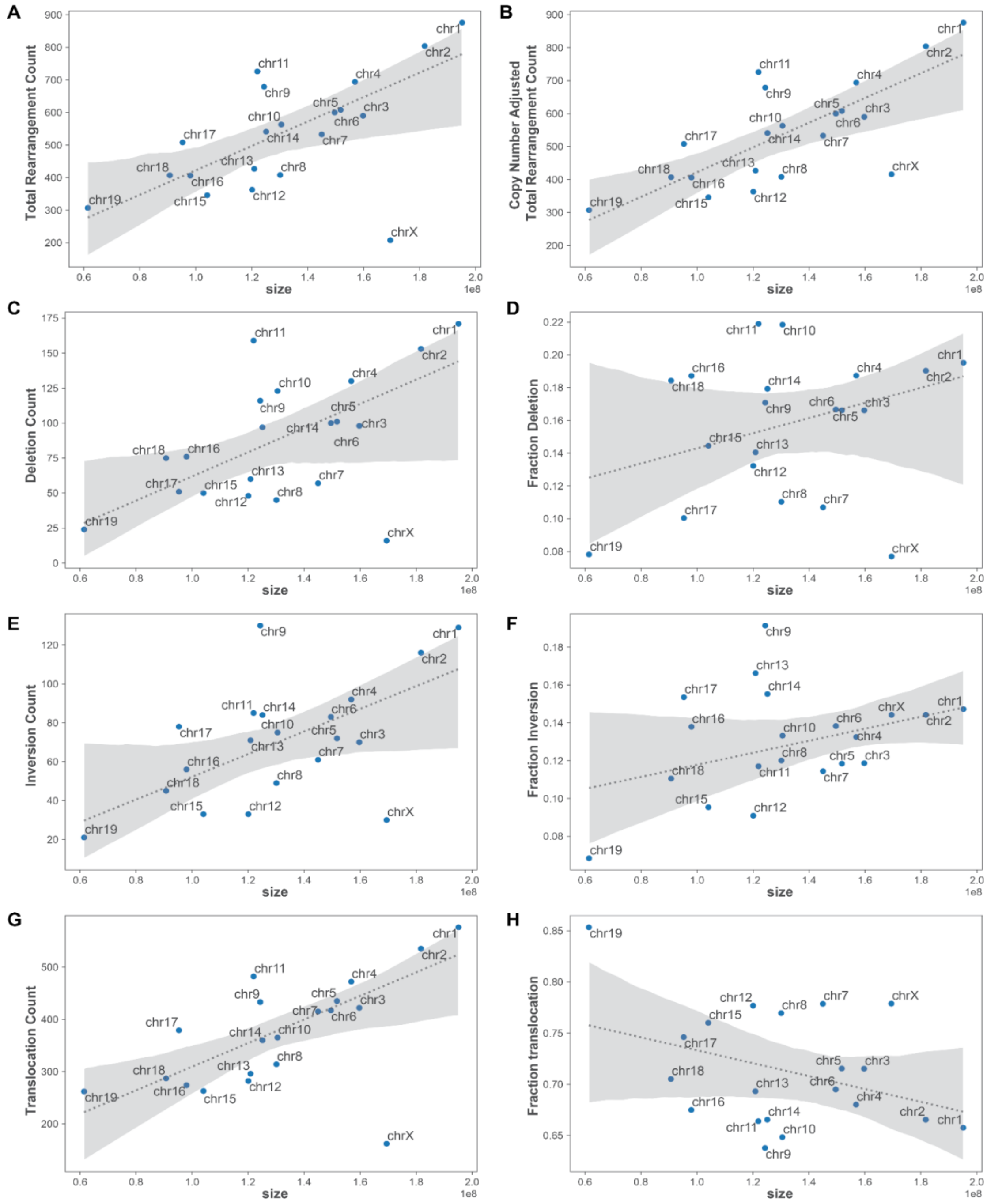

Fig. S6. Distribution of number or fraction of rearrangements of each type across chromosomes. All plots in this figure depict a scatter plot of the distribution of all rearrangements detected at 72h post-Cre transfection across mouse chromosomes except chrY, with the size of the chromosomes depicted along the *X*-axis. The dotted line indicates linear regression model fit and the shaded gray areas the 95% confidence interval. The *y*-axis of each panel is either: **A)** the total number of events detected; **B)** the total number of events detected with the number of events on the X chromosome multiplied by 2 to normalize for copy number; **C)**, **E)**, **G)** the total number of deletions **(C)**, inversions **(E)** or translocations **(G)**, respectively; **D)**, **F)**, **H)** the proportion of rearrangements on a given chromosome that are deletions **(D)**, inversions **(E)** and **(G)** translocations, respectively.

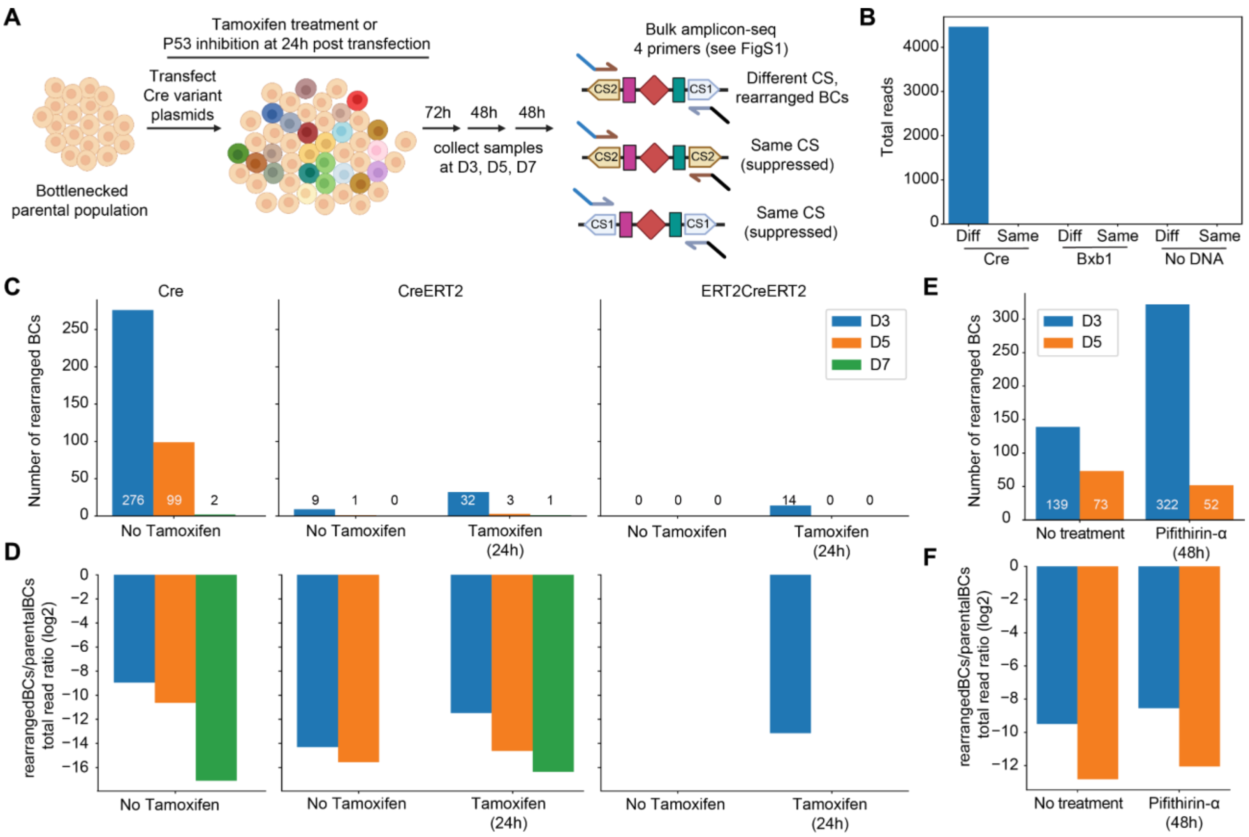

Fig. S7. Rearrangements are not stably maintained in the post-Cre induction cell population and cannot be rescued by inducible Cre variants nor by p53 inhibition. A) Schematic of the long-term culture experiments with Cre variants or p53 inhibition. The possible products from the 4 primer amplicon-seq strategy (also see **Fig. S1**) are depicted to the right. **B)** Total number of reads from 4 primer amplicon-seq data generated from Cre, Bxb1 or No DNA transfected cells that contain rearranged barcode pairs. Bars are split based on whether the reads contain the same or different (diff) capture sequence on the same molecule. **C)** Number of rearranged barcode (BC) combinations detected at day 3, 5 or 7 post transfection with Cre, CreERT2 or ERT2CreERT2. Cells were either untreated or treated with tamoxifen (0.5µM) for 24 hours. **D)** Log2 ratio of total reads with rearranged BC combinations to parental BC combinations in each sample. **E)** Similar to panel **C**, the number of rearranged BCs detected at day 3 or 5 for Cre-transfected cells with or without p53 inhibitor (Pifithrin-α, 20µM). **F)** Similar to panel **D**, Log2 ratio of total reads with rearranged BC combinations to parental BC combinations for samples with or without p53 inhibitor (Pifithrin-α, 20µM). Data presented in this figure is from one replicate.

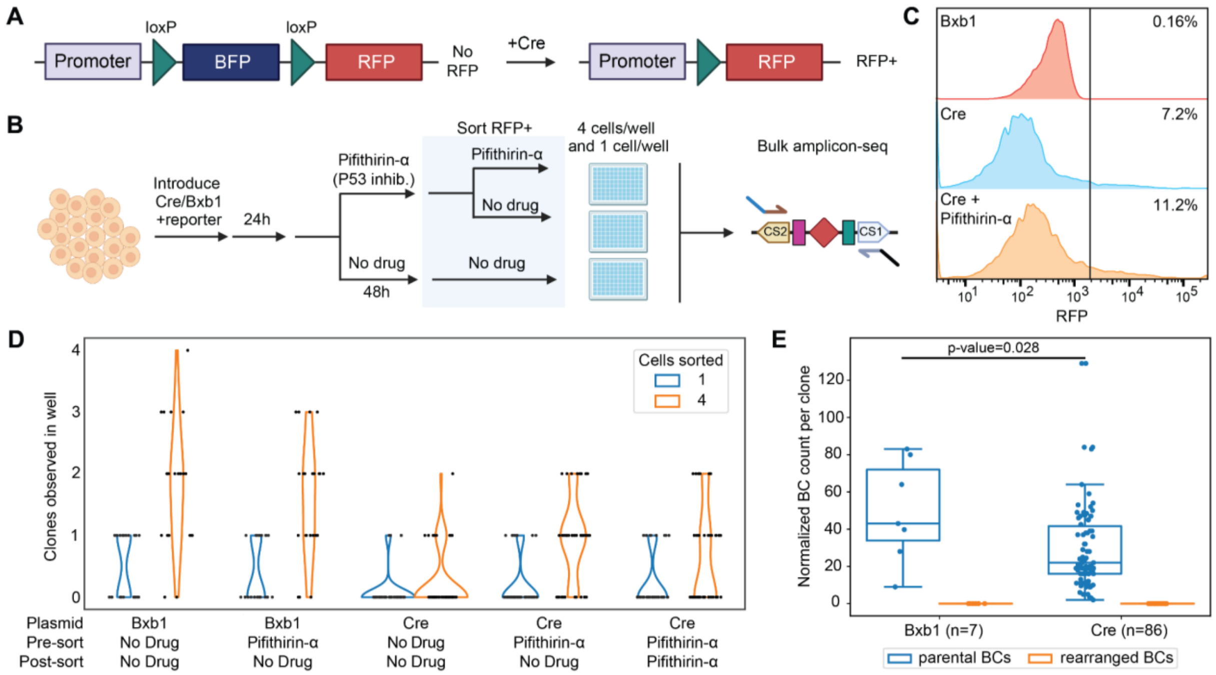

Fig. S8. Single-cell sorting does not yield long-lived clones with rearrangements. A) The reporter used for this experiment encodes a floxed blue fluorescent protein (BFP) gene which is excised in the presence of Cre to constitutively express a red fluorescent protein (RFP), thus serving as a marker for Cre activity. **B)** Schematic of the single-cell sorting experiment. Cells were transfected with either Cre or Bxb1 recombinase and optionally treated with the P53 inhibitor Pifithrin-α for 48 hours before sorting out either 4 or 1 RFP positive cell(s) into single wells of 96 well plates. Genomic DNA was extracted from clones and barcodes they contained were detected using bulk amplicon-seq. **C)** Flow cytometry traces of cell populations transfected and treated as indicated. The percentage in each panel reflects the RFP positive proportion of the population. **D)** Violin plots depicting the number of clones observed by eye per well 7 days after sorting, separated by the number of cells initially sorted into that well. **E)** Boxplots of the number of parental or rearranged barcode combinations (BCs) observed per well, normalized for the number of clones that were observed in that well. The horizontal solid line indicates the median, the length of the box depicts the interquartile range and the whiskers depict the extent of the distribution minus outliers. Data presented in this figure is from one replicate.

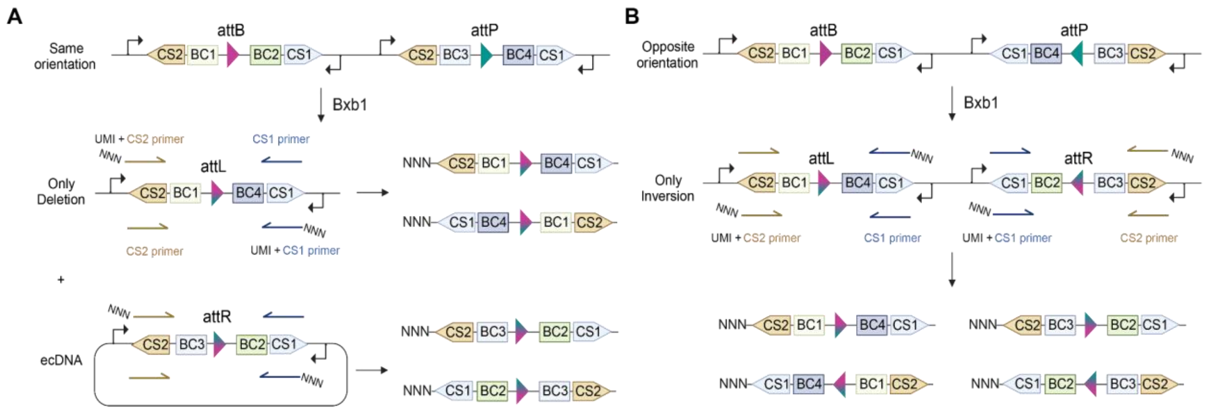

Fig. S9. Schematic of Bxb1 *Genome-shuffle-seq* and amplicon-seq library construction. Two Bxb1 shuffle cassettes bearing attB and attP sites respectively are integrated into the genome *in cis*. Sites are flanked by unique barcodes (BC1, BC2) and 10x Genomics capture sequences (CS1, CS2) serve as binding sites for PCR primers for sequencing library construction. Recombination between the two sites results in the formation of novel attL and attR sites that are resistant to further recombination in the presence of Bxb1 alone. **A)** Recombination between two sites in the same orientation, can result only in the formation of a genomic deletion + extrachromosomal circular DNA (ecDNA). **B)** Recombination between two sites in the opposite orientation can result only in the formation of a genomic inversion. Other annotations are the same as in **Fig. S1**. Importantly, no Bxb1 mediated rearrangements lead to the formation of cassettes with the same primer binding site on both sides, eliminating risk of suppressive PCR and ensuring all events are detectable in amplicon-seq.

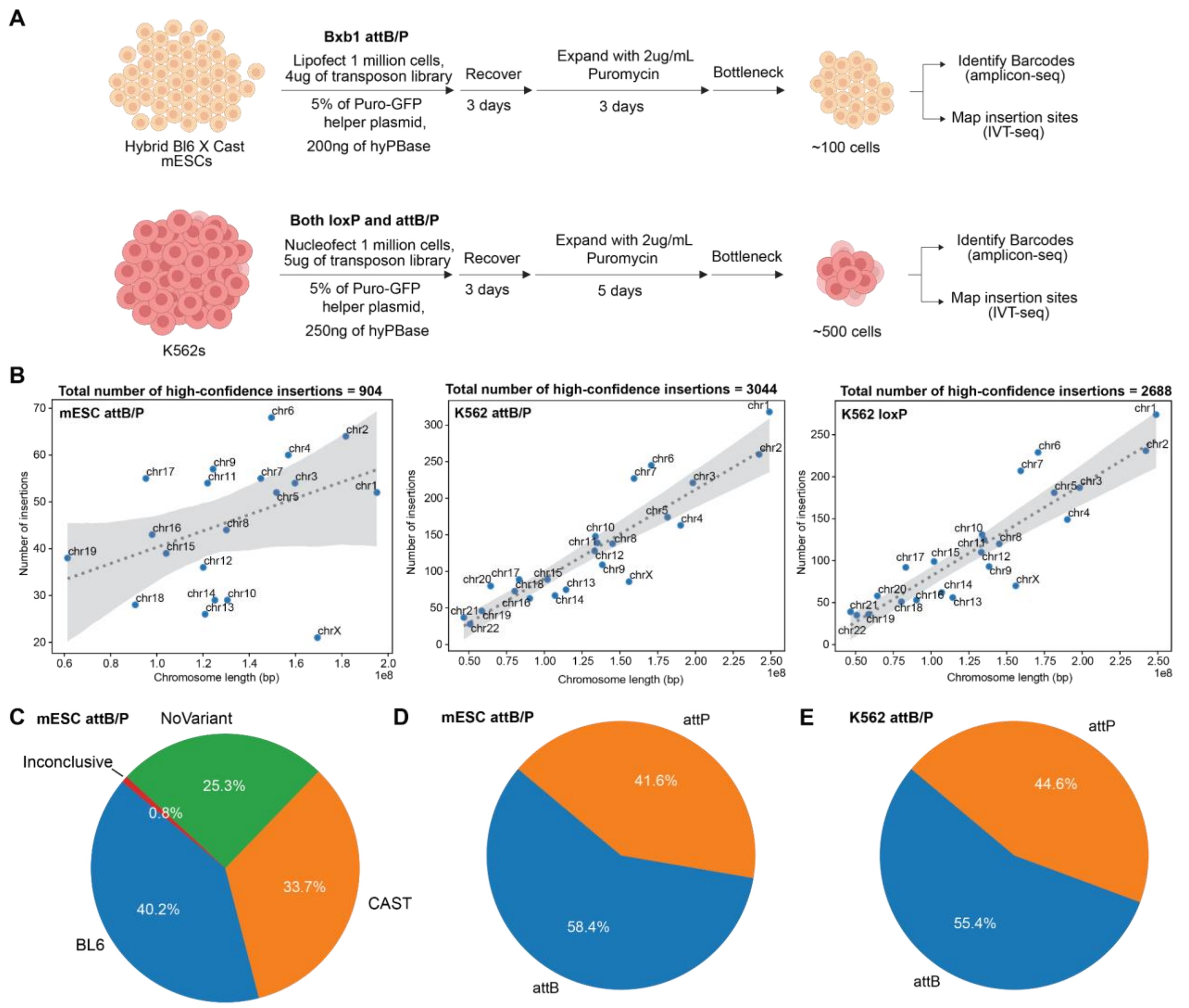

Fig. S10. Integration and mapping of attB/P and loxP shuffle cassette libraries in mESCs and K562s. A) Schematic of experimental protocol for the generation of mESC and K562 populations with high MOI integration of shuffle cassettes. **B)** Number of insertion sites with unique barcodes (*y*-axis) across chromosomes of varying lengths (*X*-axis). The dotted line indicates a linear regression model fit and the shaded gray areas the 95% confidence interval. We have not corrected here for chromosomal copy number alterations in either line. **C)** Pie chart depicting the distribution of assignments to BL6 or CAST alleles for attB/P shuffle cassettes whose genomic coordinates were mapped with high confidence in mESCs. **D)** and **E)** Pie chart depicting the distribution of attB and attP sites mapped at high confidence in mESCs and K562s respectively.

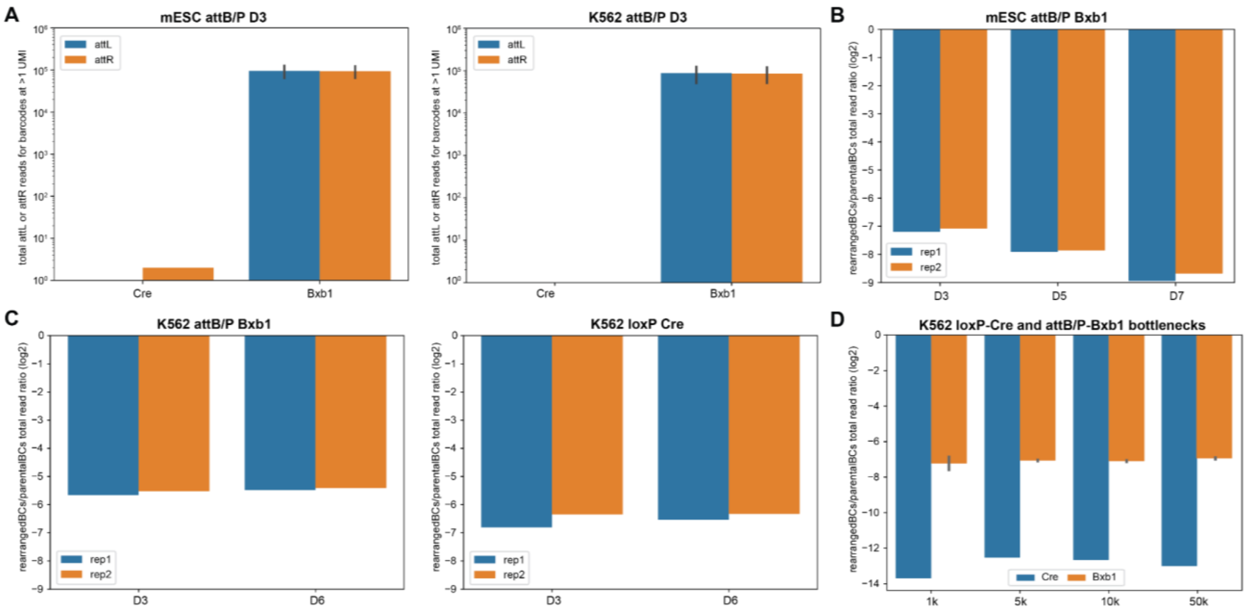

Fig. S11. Bxb1 *Genome-shuffle-seq* is specific and leads to more stable rearrangements than Cre in mESCs and K562s. A) Barplot depicting the number of reads for barcode combinations detected at ≥2 UMI that reflect an attL or attR site in Cre or Bxb1 transfected attB/P mESCs and K562s. The Bxb1 data in this figure reflects two technical replicates, and error bars represent 95% confidence intervals. The Cre data is from one technical replicate. **B-D)** Log2 ratio of total reads with rearranged BC combinations to parental BC combinations in **B)** mESC attB/P cells treated with Bxb1; **C)** K562 attB/P cells treated with Bxb1 and K562 loxP cells treated with Cre; **D)** Bottlenecked and expanded populations derived from K562 loxP cells treated with Cre and K562 attB/P cells treated with Bxb1. Data is from one technical replicate per bottlenecked population. The Bxb1 bars reflect amplicon-seq data from two independent bottlenecks and error bars represent 95% confidence intervals.

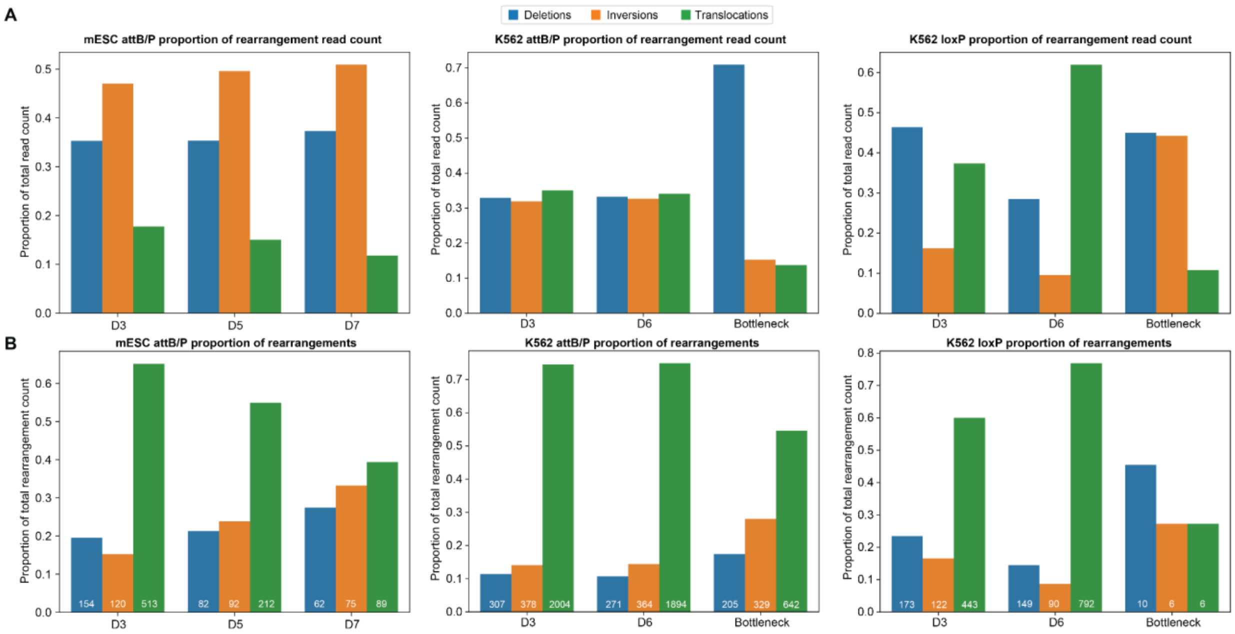

Fig. S12. Distributions of Bxb1-induced rearrangements in mESCs and K562s. A) Barplots depicting the proportion of deletions, inversions and translocation reads in the total number of reads reflecting rearrangements in that sample. **B)** Barplots depicting the proportion of deletions, inversions and translocation counts in the total number of rearrangements detected in that sample. The number at the bottom of each bar is the number of events of that rearrangement class detected at that time point.

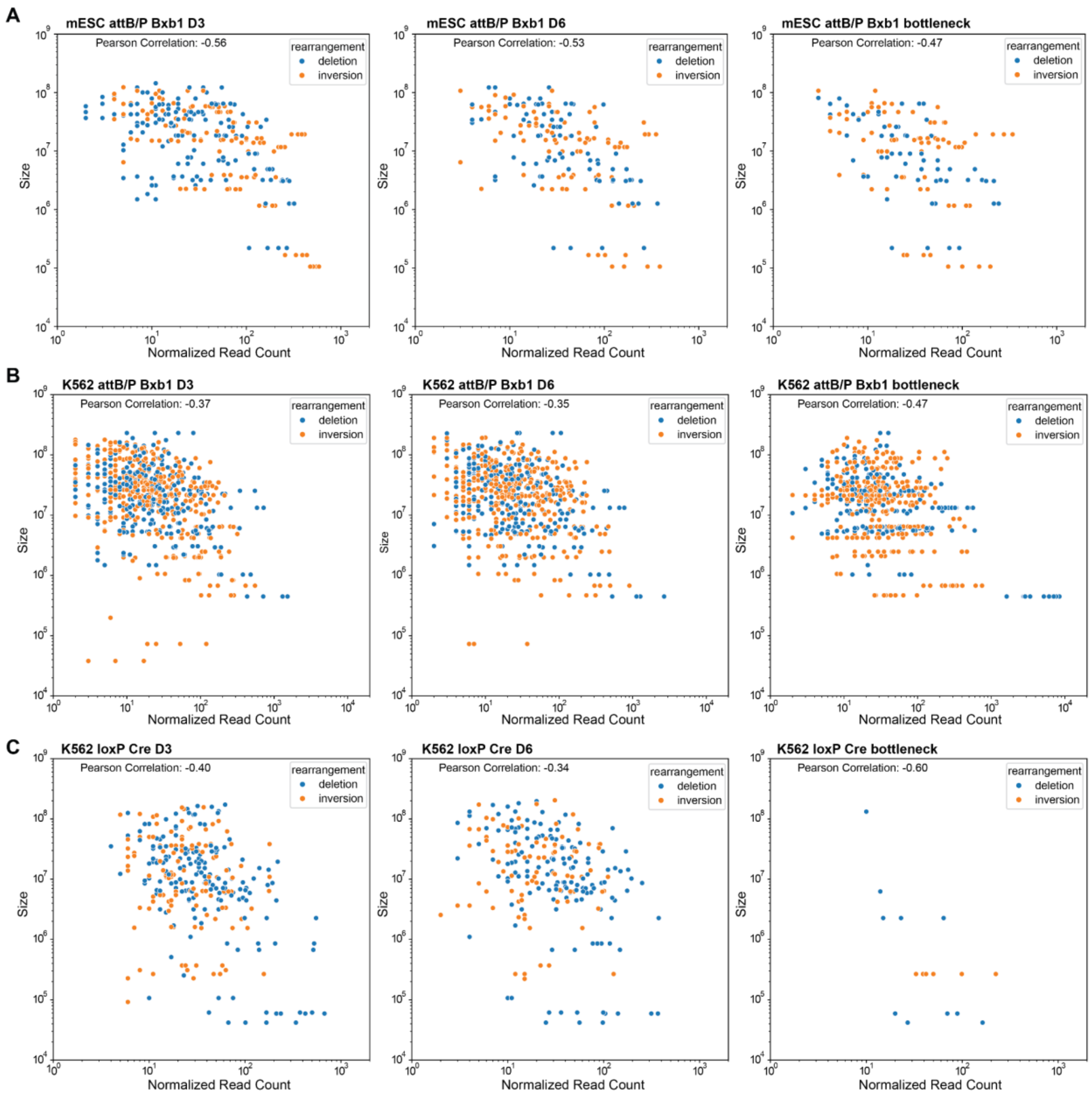

Fig. S13. Abundance and size of inversions and deletions are inversely correlated. Scatter plot of rearrangement size (*y*-axis) vs. normalized read count (*X*-axis) for deletions and inversions detected at the indicated time points in attB/P mESCs treated with Bxb1, attB/P K562s treated with Bxb1 and loxP K562s treated with Cre. Pearson correlation is calculated between the log10 values of the two metrics.

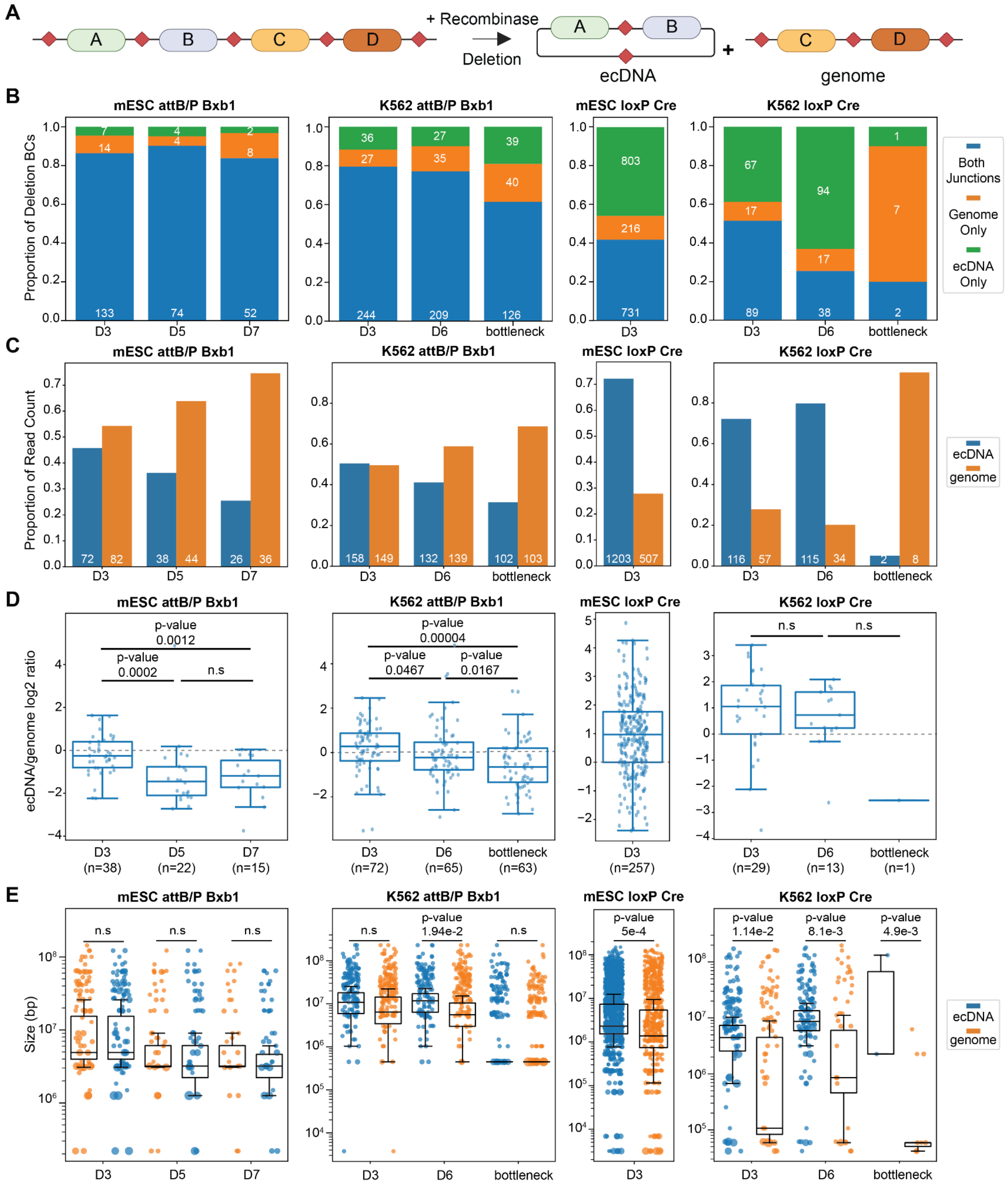

Fig. S14. Hundreds of ecDNAs are launched by *Genome-shuffle-seq*. A) Schematic of extrachromosomal circular DNA (ecDNA) formation during Cre-mediated deletion. The genomic deletion and ecDNA species are expected to have 1:1 stoichiometry at the time of formation. **B)** Stacked barplot depicting the proportion of rearranged barcodes called as deletions for which a matched barcode pair reflecting the other junction from the deletion event is detected within the

same biological sample. In this analysis, technical amplicon-seq replicates coming from the same biological replicate were treated as one and independent transfections or bottlenecking events were treated as separate biological replicates. Inset number reflects the number of events in each category. **C)** Barplot depicting the proportion of total read counts reflecting deletion events that come from the ecDNA vs. the genomic deletion scar in the samples at the indicated time points. Number at the base of the bar reflects the number of events in each category. **D)** Boxplot of the log2 ratio of read counts for the barcode pairs that represent the ecDNA vs. genomic copy for the set of ecDNA-genomic deletion pairs that were both detected in the same sample. In this analysis, technical amplicon-seq replicates coming from the same biological replicate were treated as one and independent transfections or bottlenecking events were treated as separate biological replicates. The number of such pairs is indicated underneath each sample. The horizontal solid line indicates the median, the length of the box depicts the interquartile range of the distribution and the whiskers depict the rest of the distribution excluding outliers. Depicted p-value is calculated using the non-parametric Mann-Whitney U test. **E)** Boxplots of the size of ecDNAs and genomic deletion scars, weighted by their read count at the indicated time point. The horizontal solid line indicates the median, the length of the box depicts the interquartile range and the whiskers depict the extent of the distribution minus outliers. The underlying distribution is depicted by the overlaid points, with the size of each bubble reflecting the relative read count. Depicted p-value is calculated using a bootstrap analysis with 10,000 iterations, resampling the distribution with replacement.

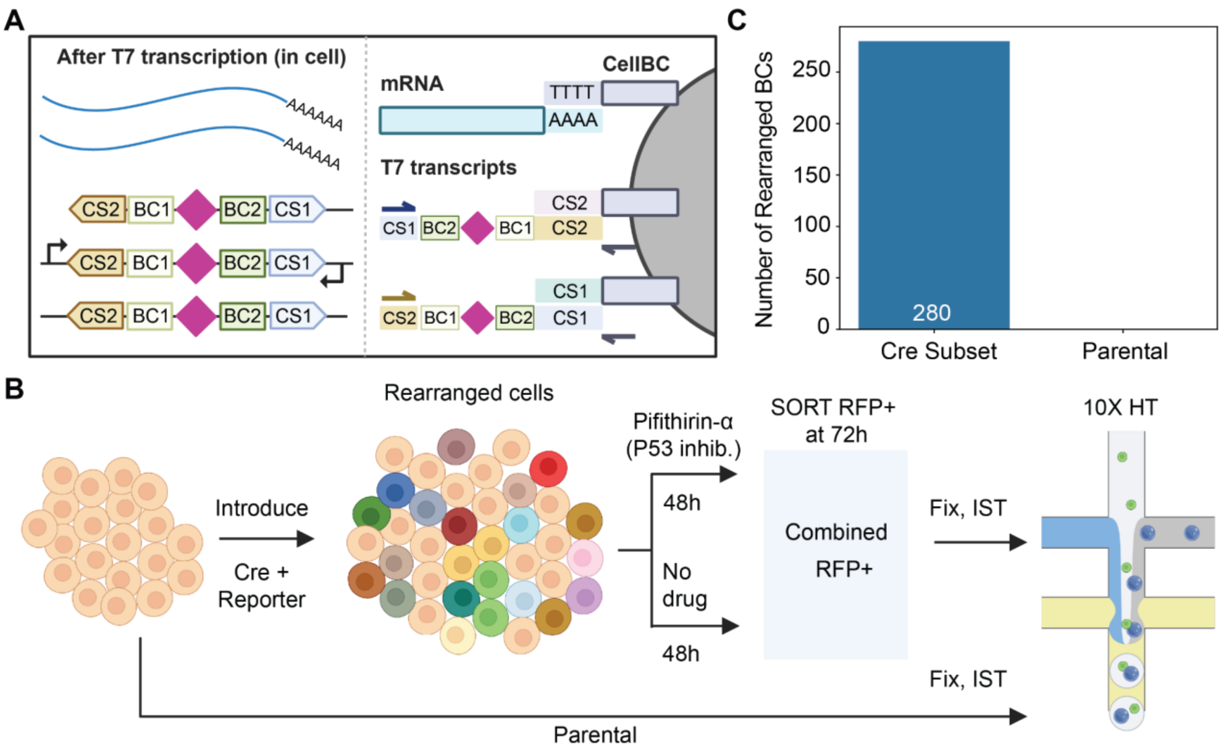

Fig. S15. Single-cell detection of rearranged barcodes is specific to recombinase-treated cells. A) After fixation and IVT with T7 polymerase, cells contain both mRNA from endogenous genes as well as RNA from shuffle cassettes. Both of these RNA species can be captured using 10X Genomics gel beads that contain complementary sequences for the polyA on mRNA and capture sequence 1 and 2 (CS1, CS2) found on the T7 derived shuffle transcripts. **B)** Experimental schematic. At 72h post transfection, cells were sorted based on the activity of the Cre reporter, methanol-fixed, subjected to T7 IVT and then sc-RNA-seq on the 10x Genomics platform. **C)** Number of novel barcode combinations detected either in the whole parental sample or a downsampled subset of the Cre sample in scRNA-seq.

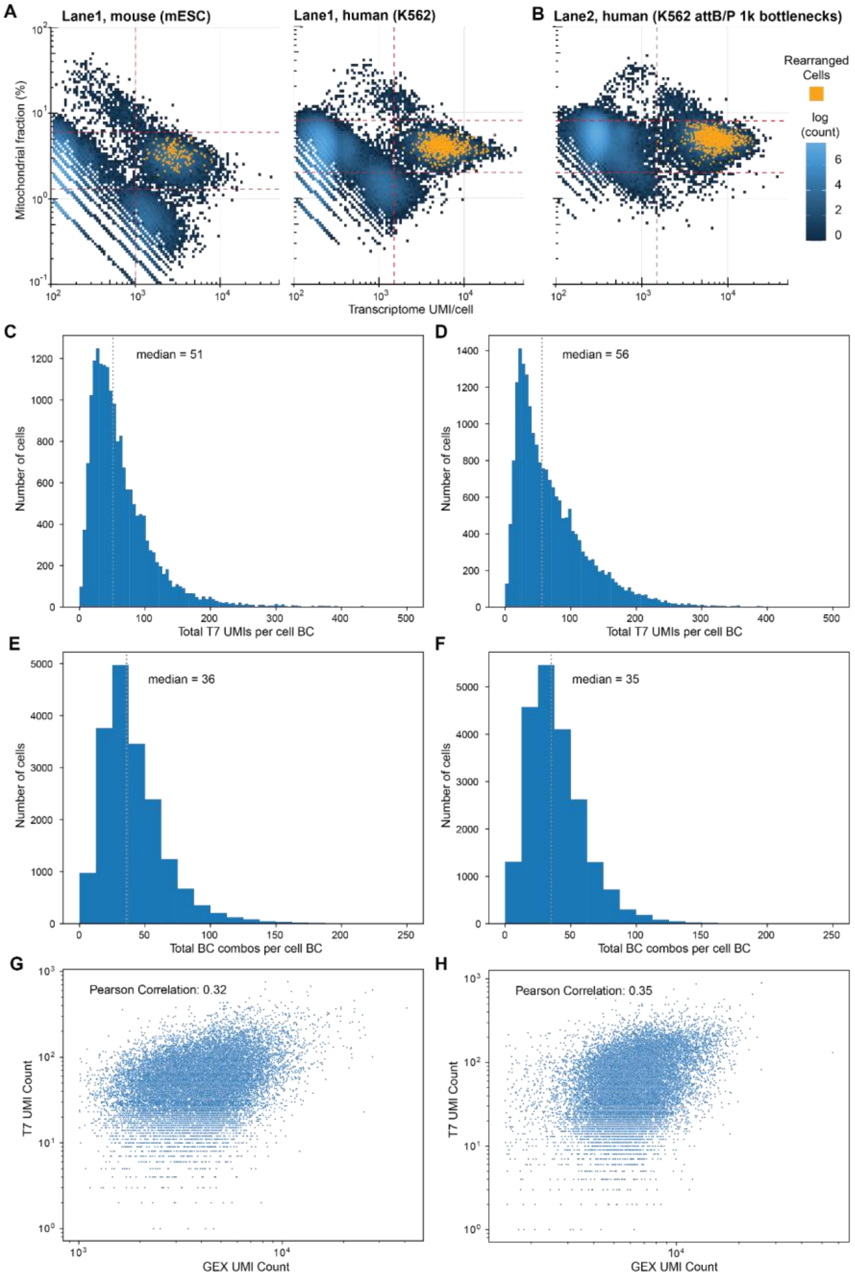

Fig. S16. High-quality transcriptomes are recovered along with T7-transcribed barcodes post methanol fixation. A) and B) Scatter plots of mitochondrial RNA fraction vs. transcriptome unique molecular identifier (UMI) counts per cell detected in Lane 1 and Lane 2. Red lines indicate thresholds that indicate the cells that were carried forward in the analysis. All cells within these thresholds that are associated with a rearranged barcode pair at ≥2 UMI are colored yellow. **C) and D)** Histogram of total T7 derived UMIs per cell barcode (BC) with the median value represented by a vertical dotted gray line in Lane 1 and Lane 2 respectively. **E) and F)** Histogram of total shuffle BC combinations detected per cell in T7 transcripts in Lane 1 and Lane 2 respectively. Median value is again depicted by a vertical dotted gray line. **G) and H)** Scatter plot of total transcriptome UMI counts (*X*-axis) versus total T7 UMI count per cell (*y*-axis) in Lane 1 and Lane 2 respectively.

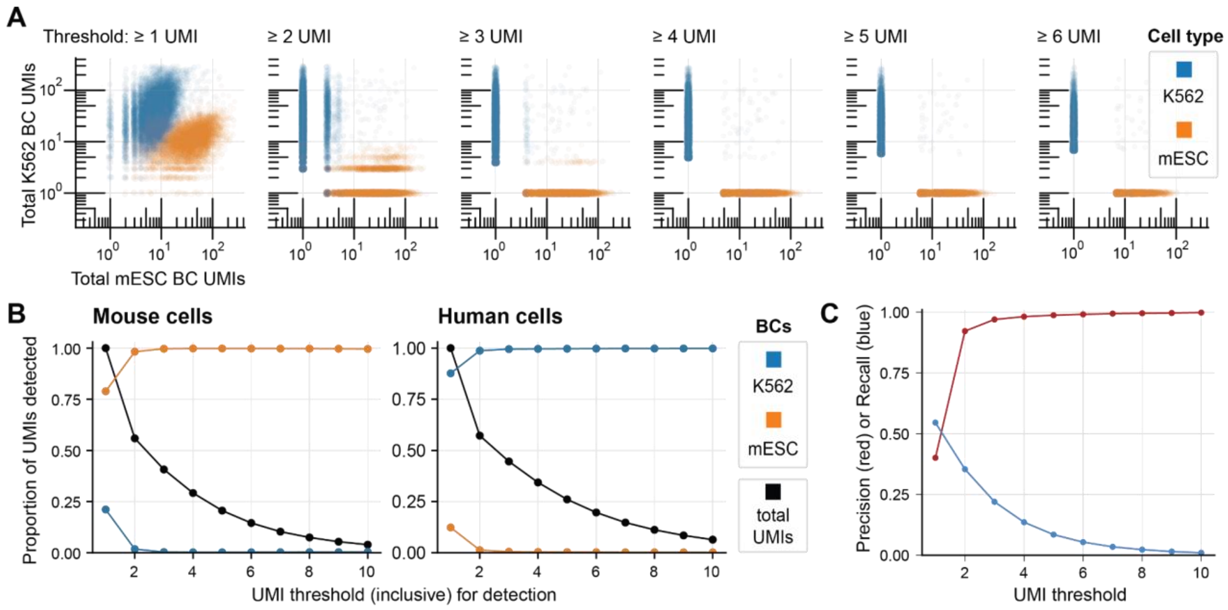

Fig. S17. Precision of T7 transcript detection is high at the ≥2 UMI threshold. A) Barnyard plots of total T7 UMIs detected in mESCs or K562 cells at various UMI thresholds per barcode pair. Each point represents a cell, coloured by assignment to mESC or K562. *X*-axis represents counts from shuffle-cassette barcodes originating from mESCs and *y*-axis represents counts from shuffle-cassette barcodes originating from K562s. Different panels correspond to different UMI threshold for detection of shuffle-cassette barcodes. **B)** Proportion of UMIs for species-specific barcodes from T7 transcripts detected in mouse or human cells at various UMI thresholds. The black line indicates the proportion of UMIs detected in cells of the indicated species at the respective UMI thresholds **C**) Mean precision and recall of detected T7 barcodes in Lane 1 cells compared to the expected barcodes list from respective clonotypes to which the cells are assigned (*y*-axis), at various UMI thresholds (*X*-axis). Only major clonotypes (n=123) with 10 cells or more assigned are included in the precision recall analysis.

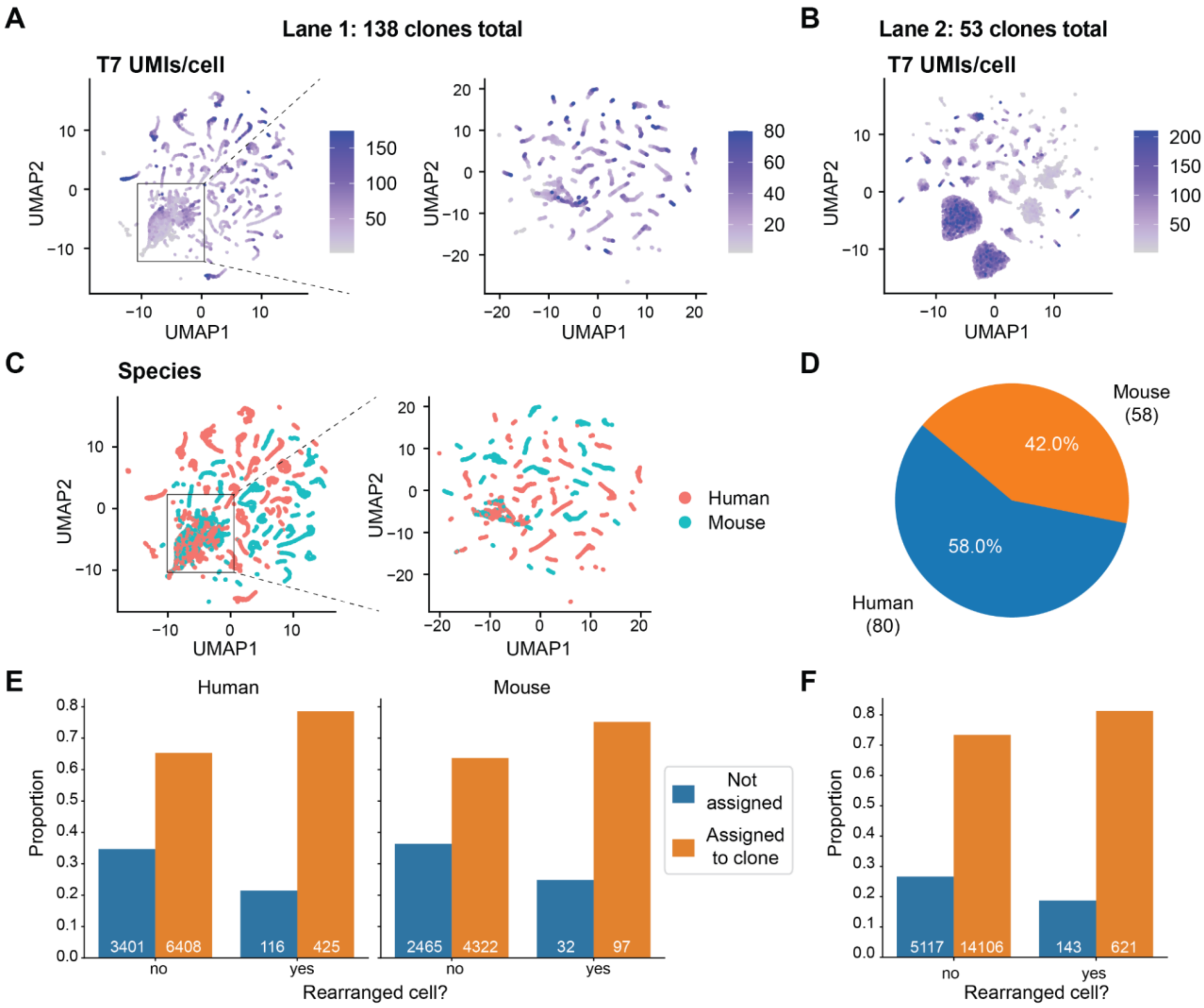

Fig. S18. Assignment of cells to clonotypes based on the complement of T7 barcodes detected. **A)** In Lane 1, 11,252 cells were assigned to 138 independent clonotypes. Here, these cells are visualized in UMAP space, colored by clone assignment. The plot on the right was generated by iterative dimensional reduction on the indicated subset of cells from the global UMAP on the left. Cells are colored by the total number of T7 derived unique molecular identifiers (UMIs) detected in that cell. **B)** In Lane 2, 14,727 cells were assigned to 53 clonotypes. Other annotations are the same as in panel A). **C)** The cells assigned to clones in Lane 1, colored by the species of origin based on the transcriptome of each cell. Clones are largely composed of cells coming from a single species as expected. **D)** Pie chart depicting the number of human (K562) and mouse (mESC) clones detected in single-cell data from Lane 1. **E-F)** Barplot of the proportion of cells in each category that were assigned to a clone for Lane 1 (**E**) or Lane 2 (**F**). Clonotype assignment is determined by the set of T7 barcodes (BCs) within them, detected with ≥2 UMI. Cells were considered assigned to a clone if at least 75% of the T7 BCs detected in that cell at ≥2 UMI belong to that specific clone. The number of cells in each category is indicated by the number at the base of each bar.

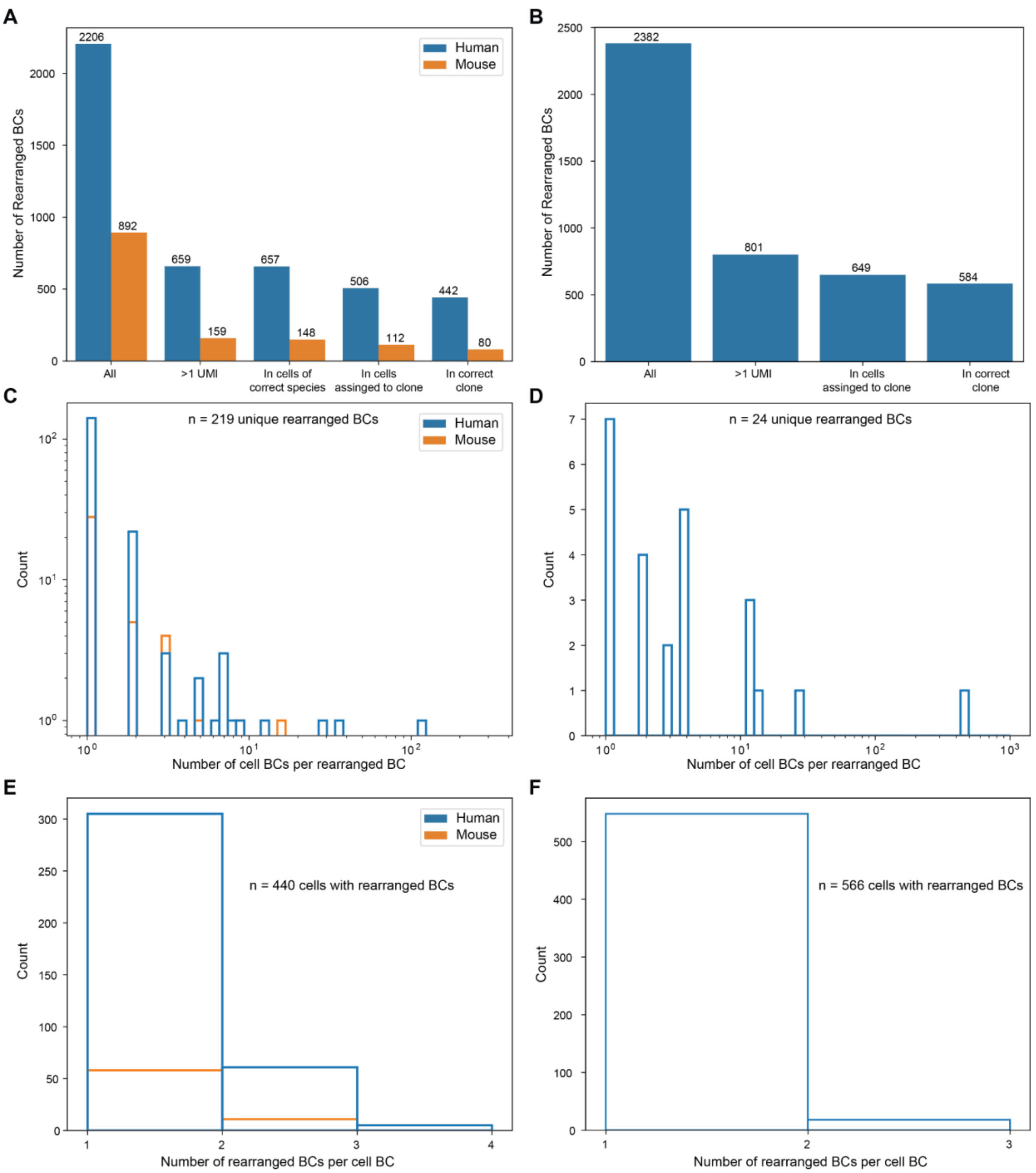

Fig. S19. Characteristics of rearranged T7 derived barcodes in single-cell data. A-B) Number of rearranged barcode (BC) combinations that are detected in Lane 1 (**A**) and Lane 2 (**B**), at successive stages of filtering: all cells, rearrangements detected at ≥2 UMI, rearrangements at ≥2 UMI in cells that could be assigned to a clonotype and lastly, rearrangements for which the identity of the rearranged BC pair was congruent with the clonotype assignment. That is, both BCs were detected in the same parental clone. **C-D)** Histogram depicting the number of unique cell barcodes associated with a particular rearrangement in Lane 1 (**C**) and Lane 2 (**D**). **E-F)** Histogram depicting the number of rearranged barcodes detected per cell BC in Lane 1 (**E**) and Lane 2 (**F**).

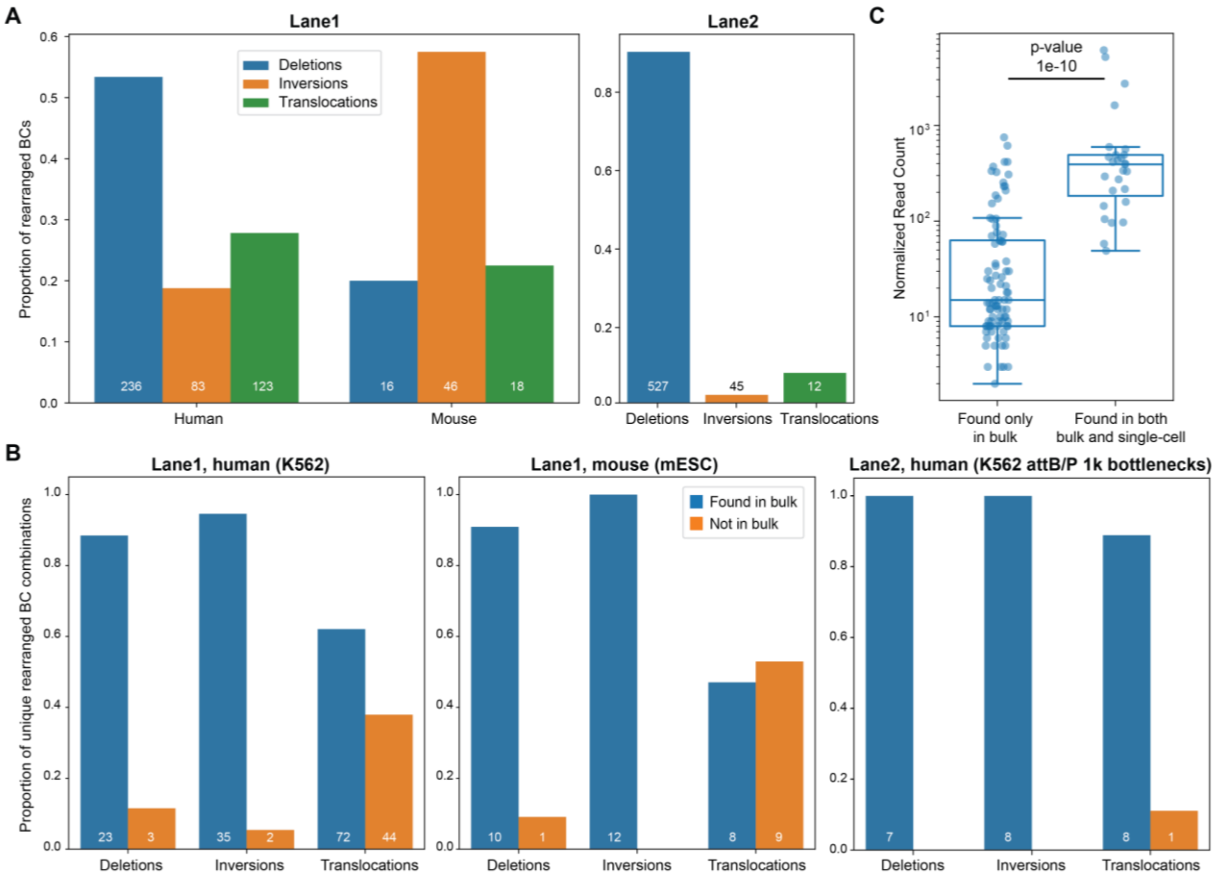

Fig. S20. Distribution of rearrangements detected in single-cell data and comparison to bulk amplicon-seq. A) Barplot depicting the proportion of total rearranged barcodes detected across all cells, filtered as described in S18A for Lane 1 and Lane 2. The number of events in each category are indicated at the base of the bar. **B)** The proportion of the unique set of rearranged barcodes corresponding to inferred deletions, inversions and translocations in each sample and their detection status in matched, bulk amplicon-seq data is depicted as a barplot. **C)** Boxplot of the normalized read count from bulk amplicon-seq data for the set of rearranged barcodes found only in the bulk amplicon-seq data from the K562 1k bottlenecks, or those detected in both the bulk and Lane 2 single-cell data. The p-value is calculated using the non-parametric Mann-Whitney U-test.

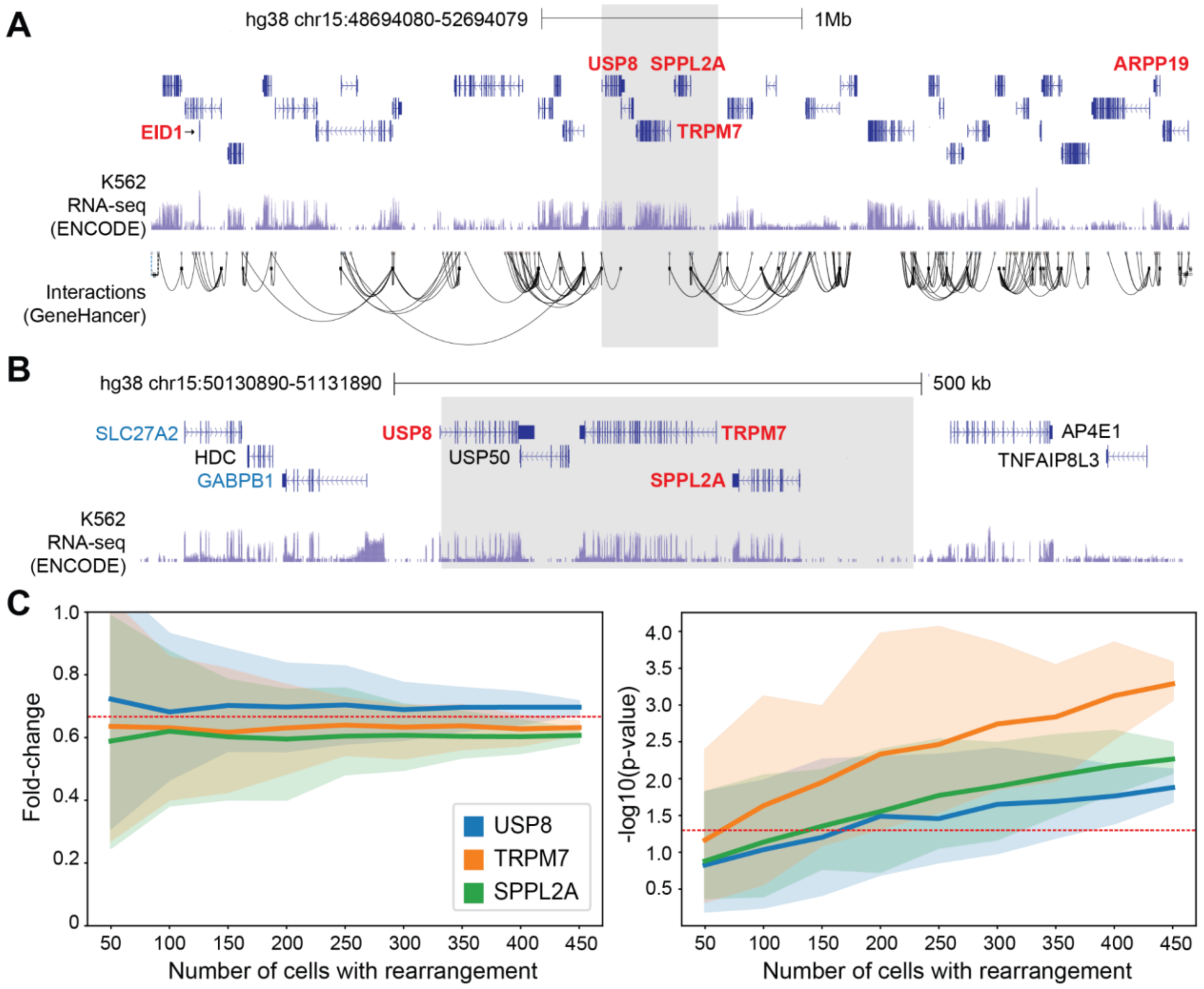

Fig. S21. Genomic neighborhood of the chromosome 15 deletion and downsampling analysis. **A-B)** Graphics from the UCSC Genome Browser of the indicated coordinates. K562 bulk RNA-seq data is from the ENCODE project and gene-enhancer interactions are from the GeneHancer database *79, 80*). Genes with nominally statistically significant decreases in gene expression in deletion-containing cells are labeled in red. In panel **B**, genes tested but insignificant are also labeled in blue, and genes insufficiently expressed to qualify for being tested are labeled in black. The gray shaded region indicates the extent of the deletion. **C)** Effects of sample size of cells with the rearrangement on p-value and estimated fold-change for the three genes in the deleted region. Shaded regions represent the 95th percentile interval of outcomes after 100 sampling trials. Horizontal dotted lines represent the significance level of $p < 0.05$ and the expected fold-change of 0.66, respectively, without correction for multiple hypothesis testing.

List of Supplementary Tables

Table S1. List of primers and their sequences used in this study.

Table S2. List of parental shuffle cassette insertion positions in loxPsym+ mESCs.

Table S3. All rearranged barcodes detected in 2-primer bulk amplicon-seq at day 3 post Cre treatment in loxPsym+ mESCs (data used in Figs. 3, 5, S4-6, S11).

Table S4. All rearranged barcodes detected in both technical replicates of 2-primer bulk amplicon-seq at day 3 post Cre treatment in loxPsym+ mESCs (data used in Figs. 3, 5, S4-6, S11).

Table S5. List of parental shuffle cassette insertion positions in attB/P+ mESCs.

Table S6. List of parental shuffle cassette insertion positions in loxPsym+ K562s.

Table S7. List of parental shuffle cassette insertion positions in attB/P+ K562s.

Table S8. All rearranged barcodes detected in 4-primer bulk amplicon-seq at days 3, 5 and 7, post Bxb1 treatment in attB/P+ mESCs (data used in Figs. 4, S11-14).

Table S9. All rearranged barcodes detected in 4-primer bulk amplicon-seq at days 3, 6 and bottlenecked populations, post Cre treatment in loxPsym+ K562s (data used in Figs. 4, S11-14).

Table S10. All rearranged barcodes detected in 4-primer bulk amplicon-seq at days 3, 6 and bottlenecked populations, post Bxb1 treatment in attB/P+ K562s (data used in Figs. 4, S11-14).

Table S11. All cells detected in Lane 1 of attB/P+ K562/mESC 10X experiment with associated characteristics (data used in Figs. 5, S16-20).

Table S12. All rearranged barcodes detected in Lane 1 of attB/P+ K562/mESC 10X experiment (data used in Figs.5, S16,18-20).

Table S13. Filtered rearranged barcodes detected in Lane 1 of attB/P+ K562/mESC 10X experiment at ≥2 UMI, and congruent with the assigned clone of that cell (data used in Figs. 5, S16,19-20).

Table S14. All cells detected in Lane 2 of attB/P+ K562 10X experiment with associated characteristics (data used in Figs. 5, S16,18-21).

Table S15. All rearranged barcodes detected in Lane 2 of attB/P+ K562 10X experiment (data used in Figs. 5, S16,18-21).

Table S16. Filtered rearranged barcodes detected in Lane 2 of attB/P+ K562 10X experiment at ≥2 UMI, and congruent with the assigned clone of that cell (data used in Figs. 5, S16,18-21).

Table S17. List of clonotypes and associated barcodes identified from Lane 1 and Lane 2 of attB/P+ K562/mESCs single-cell RNA sequencing.

Table S18. Assignment of cells to clonotypes based on the set of T7-derived barcodes detected within them for Lane 1 of attB/P+ K562/mESC 10X experiment.

Table S19. Assignment of cells to clonotypes based on the set of T7-derived barcodes detected within them for Lane 2 of attB/P+ K562 10X experiment.

Table S20. Details of all sequencing libraries presented in this study, as well as description of processed file and description of wells from the single-cell sorting experiment described in Fig. S8.

References and Notes

1. R. E. Mills, K. Walter, C. Stewart, R. E. Handsaker, K. Chen, C. Alkan, A. Abyzov, S. C. Yoon, K. Ye, R. K. Cheetham, A. Chinwalla, D. F. Conrad, Y. Fu, F. Grubert, I. Hajirasouliha, F. Hormozdiari, L. M. Iakoucheva, Z. Iqbal, S. Kang, J. M. Kidd, M. K. Konkel, J. Korn, E. Khurana, D. Kural, H. Y. K. Lam, J. Leng, R. Li, Y. Li, C.-Y. Lin, R. Luo, X. J. Mu, J. Nemesh, H. E. Peckham, T. Rausch, A. Scally, X. Shi, M. P. Stromberg, A. M. Stütz, A. E. Urban, J. A. Walker, J. Wu, Y. Zhang, Z. D. Zhang, M. A. Batzer, L. Ding, G. T. Marth, G. McVean, J. Sebat, M. Snyder, J. Wang, K. Ye, E. E. Eichler, M. B. Gerstein, M. E. Hurles, C. Lee, S. A. McCarroll, J. O. Korbel; 1000 Genomes Project, Mapping copy number variation by population-scale genome sequencing. *Nature* **470**, 59–65 (2011). doi:10.1038/nature09708 Medline

2. 1000 Genomes Project Consortium, A. Auton, L. D. Brooks, R. M. Durbin, E. P. Garrison, H. M. Kang, J. O. Korbel, J. L. Marchini, S. McCarthy, G. A. McVean, G. R. Abecasis, A global reference for human genetic variation. *Nature* **526**, 68–74 (2015). doi:10.1038/nature15393 Medline

3. J. R. Belyeu, H. Brand, H. Wang, X. Zhao, B. S. Pedersen, J. Feusier, M. Gupta, T. J. Nicholas, J. Brown, L. Baird, B. Devlin, S. J. Sanders, L. B. Jorde, M. E. Talkowski, A. R. Quinlan, De novo structural mutation rates and gamete-of-origin biases revealed through genome sequencing of 2,396 families. *Am. J. Hum. Genet.* **108**, 597–607 (2021). doi:10.1016/j.ajhg.2021.02.012 Medline

4. J. Weischenfeldt, O. Symmons, F. Spitz, J. O. Korbel, Phenotypic impact of genomic structural variation: Insights from and for human disease. *Nat. Rev. Genet.* **14**, 125–138 (2013). doi:10.1038/nrg3373 Medline

5. C. Chiang, A. J. Scott, J. R. Davis, E. K. Tsang, X. Li, Y. Kim, T. Hadzic, F. N. Damani, L. Ganel, S. B. Montgomery, A. Battle, D. F. Conrad, I. M. Hall; GTEx Consortium, The impact of structural variation on human gene expression. *Nat. Genet.* **49**, 692–699 (2017). doi:10.1038/ng.3834 Medline

6. G. Fudenberg, K. S. Pollard, Chromatin features constrain structural variation across evolutionary timescales. *Proc. Natl. Acad. Sci. U.S.A.* **116**, 2175–2180 (2019). doi:10.1073/pnas.1808631116 Medline

7. R. L. Collins, H. Brand, K. J. Karczewski, X. Zhao, J. Alföldi, L. C. Francioli, A. V. Khera, C. Lowther, L. D. Gauthier, H. Wang, N. A. Watts, M. Solomonson, A. O'Donnell-Luria, A. Baumann, R. Munshi, M. Walker, C. W. Whelan, Y. Huang, T. Brookings, T. Sharpe, M. R. Stone, E. Valkanas, J. Fu, G. Tiao, K. M. Laricchia, V. Ruano-Rubio, C. Stevens, N. Gupta, C. Cusick, L. Margolin, K. D. Taylor, H. J. Lin, S. S. Rich, W. S. Post, Y. I. Chen, J. I. Rotter, C. Nusbaum, A. Philippakis, E. Lander, S. Gabriel, B. M. Neale, S. Kathiresan, M. J. Daly, E. Banks, D. G. MacArthur, M. E. Talkowski, J. Gentry, N. Gupta, T. Jeandet, D. Kaplan, K. J. Karczewski, K. M. Laricchia, C. Llanwarne, E. V. Minikel, R. Munshi, B. M. Neale, S. Novod, A. H. O'Donnell-Luria, N. Petrillo, T. Poterba, D. Roazen, V. Ruano-Rubio, A. Saltzman, K. E. Samocha, M. Schleicher, C. Seed, M. Solomonson, J. Soto, G. Tiao, K. Tibbetts, C. Tolonen, C. Vittal, G. Wade, A. Wang, Q. Wang, J. S. Ware, N. A. Watts, B. Weisburd, N. Whiffin, C. A. A. Salinas, T. Ahmad, C. M. Albert, D. Ardissino, G. Atzmon, J. Barnard, L. Beaugerie, E. J.

Benjamin, M. Boehnke, L. L. Bonnycastle, E. P. Bottinger, D. W. Bowden, M. J. Bown, J. C. Chambers, J. C. Chan, D. Chasman, J. Cho, M. K. Chung, B. Cohen, A. Correa, D. Dabelea, M. J. Daly, D. Darbar, R. Duggirala, J. Dupuis, P. T. Ellinor, R. Elosua, J. Erdmann, T. Esko, M. Färkkilä, J. Florez, A. Franke, G. Getz, B. Glaser, S. J. Glatt, D. Goldstein, C. Gonzalez, L. Groop, C. Haiman, C. Hanis, M. Harms, M. Hiltunen, M. M. Holi, C. M. Hultman, M. Kallela, J. Kaprio, S. Kathiresan, B.-J. Kim, Y. J. Kim, G. Kirov, J. Kooner, S. Koskinen, H. M. Krumholz, S. Kugathasan, S. H. Kwak, M. Laakso, T. Lehtimäki, R. J. F. Loos, S. A. Lubitz, R. C. W. Ma, D. G. MacArthur, J. Marrugat, K. M. Mattila, S. McCarroll, M. I. McCarthy, D. McGovern, R. McPherson, J. B. Meigs, O. Melander, A. Metspalu, B. M. Neale, P. M. Nilsson, M. C. O'Donovan, D. Ongur, L. Orozco, M. J. Owen, C. N. A. Palmer, A. Palotie, K. S. Park, C. Pato, A. E. Pulver, N. Rahman, A. M. Remes, J. D. Rioux, S. Ripatti, D. M. Roden, D. Saleheen, V. Salomaa, N. J. Samani, J. Scharf, H. Schunkert, M. B. Shoemaker, P. Sklar, H. Soininen, H. Sokol, T. Spector, P. F. Sullivan, J. Suvisaari, E. S. Tai, Y. Y. Teo, T. Tiinamaija, M. Tsuang, D. Turner, T. Tusie-Luna, E. Vartiainen, M. P. Vawter, J. S. Ware, H. Watkins, R. K. Weersma, M. Wessman, J. G. Wilson, R. J. Xavier, K. D. Taylor, H. J. Lin, S. S. Rich, W. S. Post, Y.-D. I. Chen, J. I. Rotter, C. Nusbaum, A. Philippakis, E. Lander, S. Gabriel, B. M. Neale, S. Kathiresan, M. J. Daly, E. Banks, D. G. MacArthur, M. E. Talkowski; Genome Aggregation Database Production Team; Genome Aggregation Database Consortium, A structural variation reference for medical and population genetics. *Nature* **581**, 444–451 (2020). doi:10.1038/s41586-020-2287-8 Medline

8. J. Shendure, J. M. Akey, The origins, determinants, and consequences of human mutations. *Science* **349**, 1478–1483 (2015). doi:10.1126/science.aaa9119 Medline

9. R. Cuella-Martin, S. B. Hayward, X. Fan, X. Chen, J.-W. Huang, A. Taglialatela, G. Leuzzi, J. Zhao, R. Rabadan, C. Lu, Y. Shen, A. Ciccia, Functional interrogation of DNA damage response variants with base editing screens. *Cell* **184**, 1081–1097.e19 (2021). doi:10.1016/j.cell.2021.01.041 Medline

10. M. Kircher, C. Xiong, B. Martin, M. Schubach, F. Inoue, R. J. A. Bell, J. F. Costello, J. Shendure, N. Ahituv, Saturation mutagenesis of twenty disease-associated regulatory elements at single base-pair resolution. *Nat. Commun.* **10**, 3583 (2019). doi:10.1038/s41467-019-11526-w Medline

11. G. M. Findlay, R. M. Daza, B. Martin, M. D. Zhang, A. P. Leith, M. Gasperini, J. D. Janizek, X. Huang, L. M. Starita, J. Shendure, Accurate classification of BRCA1 variants with saturation genome editing. *Nature* **562**, 217–222 (2018). doi:10.1038/s41586-018-0461-z Medline

12. J. S. Takahashi, L. H. Pinto, M. H. Vitaterna, Forward and reverse genetic approaches to behavior in the mouse. *Science* **264**, 1724–1733 (1994). doi:10.1126/science.8209253 Medline

13. D. E. Bauer, S. C. Kamran, S. Lessard, J. Xu, Y. Fujiwara, C. Lin, Z. Shao, M. C. Canver, E. C. Smith, L. Pinello, P. J. Sabo, J. Vierstra, R. A. Voit, G.-C. Yuan, M. H. Porteus, J. A. Stamatoyannopoulos, G. Lettre, S. H. Orkin, An erythroid enhancer of BCL11A subject to genetic variation determines fetal hemoglobin level. *Science* **342**, 253–257 (2013). doi:10.1126/science.1242088 Medline

14. R. Schmidt, C. C. Ward, R. Dajani, Z. Armour-Garb, M. Ota, V. Allain, R. Hernandez, M. Layeghi, G. Xing, L. Goudy, D. Dorovskyi, C. Wang, Y. Y. Chen, C. J. Ye, B. R. Shy, L. A. Gilbert, J. Eyquem, J. K. Pritchard, S. E. Dodgson, A. Marson, Base-editing mutagenesis maps alleles to tune human T cell functions. *Nature* **625**, 805–812 (2024). doi:10.1038/s41586-023-06835-6 Medline

15. D. Pradella, M. Zhang, R. Gao, M. A. Yao, K. M. Gluchowska, Y. C. Florez, T. Mishra, G. L. Rocca, M. Weigl, Z. Jiao, H. H. M. Nguyen, F. Grimm, M. Lisi, C. Mastroleo, K. Chen, J. Luebeck, V. Bafna, C. R. Antonescu, A. Ventura, Immortalization and transformation of primary cells mediated by engineered ecDNAs. bioRxiv 2023.06.25.546239 [Preprint] (2023); doi:10.1101/2023.06.25.546239.

16. A. A. Mills, A. Bradley, From mouse to man: Generating megabase chromosome rearrangements. *Trends Genet.* **17**, 331–339 (2001). doi:10.1016/S0168-9525(01)02321-6 Medline

17. B. Zheng, M. Sage, E. A. Sheppeard, V. Jurecic, A. Bradley, Engineering mouse chromosomes with Cre-loxP: Range, efficiency, and somatic applications. *Mol. Cell. Biol.* **20**, 648–655 (2000). doi:10.1128/MCB.20.2.648-655.2000 Medline

18. K. Kraft, S. Geuer, A. J. Will, W. L. Chan, C. Paliou, M. Borschiwer, I. Harabula, L. Wittler, M. Franke, D. M. Ibrahim, B. K. Kragesteen, M. Spielmann, S. Mundlos, D. G. Lupiáñez, G. Andrey, Deletions, Inversions, Duplications: Engineering of Structural Variants using CRISPR/Cas in Mice. *Cell Rep.* **10**, 833–839 (2015). doi:10.1016/j.celrep.2015.01.016 Medline

19. K. Boroviak, B. Fu, F. Yang, B. Doe, A. Bradley, Revealing hidden complexities of genomic rearrangements generated with Cas9. *Sci. Rep.* **7**, 12867 (2017). doi:10.1038/s41598-017-12740-6 Medline

20. Y. Liu, G. Ma, Z. Gao, J. Li, J. Wang, X. Zhu, R. Ma, J. Yang, Y. Zhou, K. Hu, Y. Zhang, Y. Guo, Global chromosome rearrangement induced by CRISPR-Cas9 reshapes the genome and transcriptome of human cells. *Nucleic Acids Res.* **50**, 3456–3474 (2022). doi:10.1093/nar/gkac153 Medline

21. M. Bilodeau, S. Girard, J. Hébert, G. Sauvageau, A retroviral strategy that efficiently creates chromosomal deletions in mammalian cells. *Nat. Methods* **4**, 263–268 (2007). doi:10.1038/nmeth1011 Medline

22. S. Fortier, M. Bilodeau, T. Macrae, J.-P. Laverdure, V. Azcoitia, S. Girard, J. Chagraoui, N. Ringuette, J. Hébert, J. Krosl, N. Mayotte, G. Sauvageau, Genome-wide interrogation of Mammalian stem cell fate determinants by nested chromosome deletions. *PLOS Genet.* **6**, e1001241 (2010). doi:10.1371/journal.pgen.1001241 Medline

23. J. S. Dymond, S. M. Richardson, C. E. Coombes, T. Babatz, H. Muller, N. Annaluru, W. J. Blake, J. W. Schwerzmann, J. Dai, D. L. Lindstrom, A. C. Boeke, D. E. Gottschling, S. Chandrasegaran, J. S. Bader, J. D. Boeke, Synthetic chromosome arms function in yeast and generate phenotypic diversity by design. *Nature* **477**, 471–476 (2011). doi:10.1038/nature10403 Medline

24. S. Zhou, Y. Wu, Y. Zhao, Z. Zhang, L. Jiang, L. Liu, Y. Zhang, J. Tang, Y.-J. Yuan, Dynamics of synthetic yeast chromosome evolution shaped by hierarchical chromatin organization. *Natl. Sci. Rev.* **10**, nwad073 (2023). doi:10.1093/nsr/nwad073 Medline

25. Y. Shen, G. Stracquadanio, Y. Wang, K. Yang, L. A. Mitchell, Y. Xue, Y. Cai, T. Chen, J. S. Dymond, K. Kang, J. Gong, X. Zeng, Y. Zhang, Y. Li, Q. Feng, X. Xu, J. Wang, J. Wang, H. Yang, J. D. Boeke, J. S. Bader, SCRaMbLE generates designed combinatorial stochastic diversity in synthetic chromosomes. *Genome Res.* **26**, 36–49 (2016). doi:10.1101/gr.193433.115 Medline

26. I. Ovcharenko, G. G. Loots, M. A. Nobrega, R. C. Hardison, W. Miller, L. Stubbs, Evolution and functional classification of vertebrate gene deserts. *Genome Res.* **15**, 137–145 (2005). doi:10.1101/gr.3015505 Medline

27. M. A. Nóbrega, Y. Zhu, I. Plajzer-Frick, V. Afzal, E. M. Rubin, Megabase deletions of gene deserts result in viable mice. *Nature* **431**, 988–993 (2004). doi:10.1038/nature03022 Medline

28. N. A. Leypold, M. R. Speicher, Evolutionary conservation in noncoding genomic regions. *Trends Genet.* **37**, 903–918 (2021). doi:10.1016/j.tig.2021.06.007 Medline

29. F. M Real, S. A. Haas, P. Franchini, P. Xiong, O. Simakov, H. Kuhl, R. Schöpflin, D. Heller, M.-H. Moeinzadeh, V. Heinrich, T. Krannich, A. Bressin, M. F. Hartmann, S. A. Wudy, D. K. N. Dechmann, A. Hurtado, F. J. Barrionuevo, M. Schindler, I. Harabula, M. Osterwalder, M. Hiller, L. Wittler, A. Visel, B. Timmermann, A. Meyer, M. Vingron, R. Jiménez, S. Mundlos, D. G. Lupiáñez, The mole genome reveals regulatory rearrangements associated with adaptive intersexuality. *Science* **370**, 208–214 (2020). doi:10.1126/science.aaz2582 Medline

30. A. Kapusta, A. Suh, C. Feschotte, Dynamics of genome size evolution in birds and mammals. *Proc. Natl. Acad. Sci. U.S.A.* **114**, E1460–E1469 (2017). doi:10.1073/pnas.1616702114 Medline

31. R. H. Waterston, K. Lindblad-Toh, E. Birney, J. Rogers, J. F. Abril, P. Agarwal, R. Agarwala, R. Ainscough, M. Alexandersson, P. An, S. E. Antonarakis, J. Attwood, R. Baertsch, J. Bailey, K. Barlow, S. Beck, E. Berry, B. Birren, T. Bloom, P. Bork, M. Botcherby, N. Bray, M. R. Brent, D. G. Brown, S. D. Brown, C. Bult, J. Burton, J. Butler, R. D. Campbell, P. Carninci, S. Cawley, F. Chiaromonte, A. T. Chinwalla, D. M. Church, M. Clamp, C. Clee, F. S. Collins, L. L. Cook, R. R. Copley, A. Coulson, O. Couronne, J. Cuff, V. Curwen, T. Cutts, M. Daly, R. David, J. Davies, K. D. Delehaunty, J. Deri, E. T. Dermitzakis, C. Dewey, N. J. Dickens, M. Diekhans, S. Dodge, I. Dubchak, D. M. Dunn, S. R. Eddy, L. Elnitski, R. D. Emes, P. Eswara, E. Eyras, A. Felsenfeld, G. A. Fewell, P. Flicek, K. Foley, W. N. Frankel, L. A. Fulton, R. S. Fulton, T. S. Furey, D. Gage, R. A. Gibbs, G. Glusman, S. Gnerre, N. Goldman, L. Goodstadt, D. Grafham, T. A. Graves, E. D. Green, S. Gregory, R. Guigó, M. Guyer, R. C. Hardison, D. Haussler, Y. Hayashizaki, L. W. Hillier, A. Hinrichs, W. Hlavina, T. Holzer, F. Hsu, A. Hua, T. Hubbard, A. Hunt, I. Jackson, D. B. Jaffe, L. S. Johnson, M. Jones, T. A. Jones, A. Joy, M. Kamal, E. K. Karlsson, D. Karolchik, A. Kasprzyk, J. Kawai, E. Keibler, C. Kells, W. J. Kent, A. Kirby, D. L. Kolbe, I. Korf, R. S. Kucherlapati, E. J. Kulbokas, D. Kulp, T. Landers, J. P. Leger, S. Leonard, I. Letunic, R. Levine, J. Li, M. Li, C. Lloyd, S. Lucas, B. Ma, D. R.

Maglott, E. R. Mardis, L. Matthews, E. Mauceli, J. H. Mayer, M. McCarthy, W. R. McCombie, S. McLaren, K. McLay, J. D. McPherson, J. Meldrim, B. Meredith, J. P. Mesirov, W. Miller, T. L. Miner, E. Mongin, K. T. Montgomery, M. Morgan, R. Mott, J. C. Mullikin, D. M. Muzny, W. E. Nash, J. O. Nelson, M. N. Nhan, R. Nicol, Z. Ning, C. Nusbaum, M. J. O'Connor, Y. Okazaki, K. Oliver, E. Overton-Larty, L. Pachter, G. Parra, K. H. Pepin, J. Peterson, P. Pevzner, R. Plumb, C. S. Pohl, A. Poliakov, T. C. Ponce, C. P. Ponting, S. Potter, M. Quail, A. Reymond, B. A. Roe, K. M. Roskin, E. M. Rubin, A. G. Rust, R. Santos, V. Sapojnikov, B. Schultz, J. Schultz, M. S. Schwartz, S. Schwartz, C. Scott, S. Seaman, S. Searle, T. Sharpe, A. Sheridan, R. Shownkeen, S. Sims, J. B. Singer, G. Slater, A. Smit, D. R. Smith, B. Spencer, A. Stabenau, N. Stange-Thomann, C. Sugnet, M. Suyama, G. Tesler, J. Thompson, D. Torrents, E. Trevaskis, J. Tromp, C. Ucla, A. Ureta-Vidal, J. P. Vinson, A. C. Von Niederhausern, C. M. Wade, M. Wall, R. J. Weber, R. B. Weiss, M. C. Wendl, A. P. West, K. Wetterstrand, R. Wheeler, S. Whelan, J. Wierzbowski, D. Willey, S. Williams, R. K. Wilson, E. Winter, K. C. Worley, D. Wyman, S. Yang, S.-P. Yang, E. M. Zdobnov, M. C. Zody, E. S. Lander, Initial sequencing and comparative analysis of the mouse genome. *Nature* **420**, 520–562 (2002). doi:10.1038/nature01262 Medline

32. J. B. Noer, O. K. Hørsdal, X. Xiang, Y. Luo, B. Regenberg, Extrachromosomal circular DNA in cancer: History, current knowledge, and methods. *Trends Genet.* **38**, 766–781 (2022). doi:10.1016/j.tig.2022.02.007 Medline

33. M. L. Leibowitz, C.-Z. Zhang, D. Pellman, Chromothripsis: A New Mechanism for Rapid Karyotype Evolution. *Annu. Rev. Genet.* **49**, 183–211 (2015). doi:10.1146/annurev-genet-120213-092228 Medline

34. R. H. Hoess, A. Wierzbicki, K. Abremski, The role of the loxP spacer region in P1 site-specific recombination. *Nucleic Acids Res.* **14**, 2287–2300 (1986). doi:10.1093/nar/14.5.2287 Medline

35. J. M. Replogle, T. M. Norman, A. Xu, J. A. Hussmann, J. Chen, J. Z. Cogan, E. J. Meer, J. M. Terry, D. P. Riordan, N. Srinivas, I. T. Fiddes, J. G. Arthur, L. J. Alvarado, K. A. Pfeiffer, T. S. Mikkelsen, J. S. Weissman, B. Adamson, Combinatorial single-cell CRISPR screens by direct guide RNA capture and targeted sequencing. *Nat. Biotechnol.* **38**, 954–961 (2020). doi:10.1038/s41587-020-0470-y Medline

36. A. Askary, L. Sanchez-Guardado, J. M. Linton, D. M. Chadly, M. W. Budde, L. Cai, C. Lois, M. B. Elowitz, In situ readout of DNA barcodes and single base edits facilitated by in vitro transcription. *Nat. Biotechnol.* **38**, 66–75 (2020). doi:10.1038/s41587-019-0299-4 Medline

37. X. Li, W. Chen, B. K. Martin, D. Calderon, C. Lee, J. Choi, F. M. Chardon, T. A. McDiarmid, R. M. Daza, H. Kim, J.-B. Lalanne, J. F. Nathans, D. S. Lee, J. Shendure, Chromatin context-dependent regulation and epigenetic manipulation of prime editing. *Cell* **187**, 2411–2427.e25 (2024). doi:10.1016/j.cell.2024.03.020 Medline

38. M. A. Eckersley-Maslin, D. Thybert, J. H. Bergmann, J. C. Marioni, P. Flicek, D. L. Spector, Random monoallelic gene expression increases upon embryonic stem cell differentiation. *Dev. Cell* **28**, 351–365 (2014). doi:10.1016/j.devcel.2014.01.017 Medline

39. R. Kalhor, K. Kalhor, L. Mejia, K. Leeper, A. Graveline, P. Mali, G. M. Church, Developmental barcoding of whole mouse via homing CRISPR. *Science* **361**, eaat9804 (2018). doi:10.1126/science.aat9804 Medline

40. T. M. Keane, L. Goodstadt, P. Danecek, M. A. White, K. Wong, B. Yalcin, A. Heger, A. Agam, G. Slater, M. Goodson, N. A. Furlotte, E. Eskin, C. Nellåker, H. Whitley, J. Cleak, D. Janowitz, P. Hernandez-Pliego, A. Edwards, T. G. Belgard, P. L. Oliver, R. E. McIntyre, A. Bhomra, J. Nicod, X. Gan, W. Yuan, L. van der Weyden, C. A. Steward, S. Bala, J. Stalker, R. Mott, R. Durbin, I. J. Jackson, A. Czechanski, J. A. Guerra-Assunção, L. R. Donahue, L. G. Reinholdt, B. A. Payseur, C. P. Ponting, E. Birney, J. Flint, D. J. Adams, Mouse genomic variation and its effect on phenotypes and gene regulation. *Nature* **477**, 289–294 (2011). doi:10.1038/nature10413 Medline

41. P. D. Siebert, A. Chenchik, D. E. Kellogg, K. A. Lukyanov, S. A. Lukyanov, An improved PCR method for walking in uncloned genomic DNA. *Nucleic Acids Res.* **23**, 1087–1088 (1995). doi:10.1093/nar/23.6.1087 Medline

42. C. Plessy, N. Bertin, H. Takahashi, R. Simone, M. Salimullah, T. Lassmann, M. Vitezic, J. Severin, S. Olivarius, D. Lazarevic, N. Hornig, V. Orlando, I. Bell, H. Gao, J. Dumais, P. Kapranov, H. Wang, C. A. Davis, T. R. Gingeras, J. Kawai, C. O. Daub, Y. Hayashizaki, S. Gustincich, P. Carninci, Linking promoters to functional transcripts in small samples with nanoCAGE and CAGEscan. *Nat. Methods* **7**, 528–534 (2010). doi:10.1038/nmeth.1470 Medline

43. A. Loonstra, M. Vooijs, H. B. Beverloo, B. A. Allak, E. van Drunen, R. Kanaar, A. Berns, J. Jonkers, Growth inhibition and DNA damage induced by Cre recombinase in mammalian cells. *Proc. Natl. Acad. Sci. U.S.A.* **98**, 9209–9214 (2001). doi:10.1073/pnas.161269798 Medline

44. M. Kurachi, S. F. Ngiow, J. Kurachi, Z. Chen, E. J. Wherry, Hidden Caveat of Inducible Cre Recombinase. *Immunity* **51**, 591–592 (2019). doi:10.1016/j.immuni.2019.09.010 Medline

45. J. Zhu, M.-T. Nguyen, E. Nakamura, J. Yang, S. Mackem, Cre-mediated recombination can induce apoptosis in vivo by activating the p53 DNA damage-induced pathway. *Genesis* **50**, 102–111 (2012). doi:10.1002/dvg.20799 Medline

46. T. Matsuda, C. L. Cepko, Controlled expression of transgenes introduced by in vivo electroporation. *Proc. Natl. Acad. Sci. U.S.A.* **104**, 1027–1032 (2007). doi:10.1073/pnas.0610155104 Medline

47. E. D. Tichy, Z. A. Stephan, A. Osterburg, G. Noel, P. J. Stambrook, Mouse embryonic stem cells undergo charontosis, a novel programmed cell death pathway dependent upon cathepsins, p53, and EndoG, in response to etoposide treatment. *Stem Cell Res.* **10**, 428–441 (2013). doi:10.1016/j.scr.2013.01.010 Medline

48. G. Ayaz, H. Yan, N. Malik, J. Huang, An Updated View of the Roles of p53 in Embryonic Stem Cells. *Stem Cells* **40**, 883–891 (2022). doi:10.1093/stmcls/sxac051 Medline

49. Z. Xu, L. Thomas, B. Davies, R. Chalmers, M. Smith, W. Brown, Accuracy and efficiency define Bxb1 integrase as the best of fifteen candidate serine recombinases for the integration of DNA into the human genome. *BMC Biotechnol.* **13**, 87 (2013). doi:10.1186/1472-6750-13-87 Medline

50. M. Jelicic, L. T. Schmitt, M. Paszkowski-Rogacz, A. Walder, N. Schubert, J. Hoersten, D. Sürün, F. Buchholz, Discovery and characterization of novel Cre-type tyrosine site-specific recombinases for advanced genome engineering. *Nucleic Acids Res.* **51**, 5285–5297 (2023). doi:10.1093/nar/gkad366 Medline

51. B. Jusiak, K. Jagtap, L. Gaidukov, X. Duportet, K. Bandara, J. Chu, L. Zhang, R. Weiss, T. K. Lu, Comparison of Integrases Identifies Bxb1-GA Mutant as the Most Efficient Site-Specific Integrase System in Mammalian Cells. *ACS Synth. Biol.* **8**, 16–24 (2019). doi:10.1021/acssynbio.8b00089 Medline

52. V. Barra, D. Fachinetti, The dark side of centromeres: Types, causes and consequences of structural abnormalities implicating centromeric DNA. *Nat. Commun.* **9**, 4340 (2018). doi:10.1038/s41467-018-06545-y Medline

53. M. D. Luecken, F. J. Theis, Current best practices in single-cell RNA-seq analysis: a tutorial. *Mol. Syst. Biol.* **15**, e8746 (2019). doi:10.15252/msb.20188746 Medline

54. M. D. Young, S. Behjati, SoupX removes ambient RNA contamination from droplet-based single-cell RNA sequencing data. *GigaScience* **9**, giaa151 (2020). doi:10.1093/gigascience/giaa151 Medline

55. A. Sziraki, Z. Lu, J. Lee, G. Banyai, S. Anderson, A. Abdulraouf, E. Metzner, A. Liao, J. Banfelder, A. Epstein, C. Schaefer, Z. Xu, Z. Zhang, L. Gan, P. T. Nelson, W. Zhou, J. Cao, A global view of aging and Alzheimer's pathogenesis-associated cell population dynamics and molecular signatures in human and mouse brains. *Nat. Genet.* **55**, 2104–2116 (2023). doi:10.1038/s41588-023-01572-y Medline

56. P. Datlinger, A. F. Rendeiro, T. Boenke, M. Senekowitsch, T. Krausgruber, D. Barreca, C. Bock, Ultra-high-throughput single-cell RNA sequencing and perturbation screening with combinatorial fluidic indexing. *Nat. Methods* **18**, 635–642 (2021). doi:10.1038/s41592-021-01153-z Medline

57. B. K. Martin, C. Qiu, E. Nichols, M. Phung, R. Green-Gladden, S. Srivatsan, R. Blecher-Gonen, B. J. Beliveau, C. Trapnell, J. Cao, J. Shendure, Optimized single-nucleus transcriptional profiling by combinatorial indexing. *Nat. Protoc.* **18**, 188–207 (2023). doi:10.1038/s41596-022-00752-0 Medline

58. A. Dixit, O. Parnas, B. Li, J. Chen, C. P. Fulco, L. Jerby-Arnon, N. D. Marjanovic, D. Dionne, T. Burks, R. Raychowdhury, B. Adamson, T. M. Norman, E. S. Lander, J. S. Weissman, N. Friedman, A. Regev, Perturb-Seq: Dissecting Molecular Circuits with Scalable Single-Cell RNA Profiling of Pooled Genetic Screens. *Cell* **167**, 1853–1866.e17 (2016). doi:10.1016/j.cell.2016.11.038 Medline

59. P. Datlinger, A. F. Rendeiro, C. Schmidl, T. Krausgruber, P. Traxler, J. Klughammer, L. C. Schuster, A. Kuchler, D. Alpar, C. Bock, Pooled CRISPR screening with single-cell transcriptome readout. *Nat. Methods* **14**, 297–301 (2017). doi:10.1038/nmeth.4177 Medline

60. S. Pinglay, M. Bulajić, D. P. Rahe, E. Huang, R. Brosh, N. E. Mamrak, B. R. King, S. German, J. A. Cadley, L. Rieber, N. Easo, T. Lionnet, S. Mahony, M. T. Maurano, L. J. Holt, E. O. Mazzoni, J. D. Boeke, Synthetic regulatory reconstitution reveals principles

of mammalian *Hox* cluster regulation. *Science* **377**, eabk2820 (2022). doi:10.1126/science.abk2820 Medline

61. J. Koeppel, R. Ferreira, T. Vanderstichele, L. M. Riedmayr, E. M. Peets, G. Girling, J. Weller, F. G. Liberante, T. Ellis, G. M. Church, L. Parts, Randomizing the human genome by engineering recombination between repeat elements. bioRxiv 2024.01.22.576745 [Preprint] (2020); doi:10.1101/2024.01.22.576745.

62. X. Xu, F. Meier, B. A. Blount, I. S. Pretorius, T. Ellis, I. T. Paulsen, T. C. Williams, Trimming the genomic fat: Minimising and re-functionalising genomes using synthetic biology. *Nat. Commun.* **14**, 1984 (2023). doi:10.1038/s41467-023-37748-7 Medline

63. J.-B. Lalanne, S. G. Regalado, S. Domcke, D. Calderon, B. K. Martin, X. Li, T. Li, C. C. Suiter, C. Lee, C. Trapnell, J. Shendure, Multiplex profiling of developmental cis-regulatory elements with quantitative single-cell expression reporters. *Nat. Methods* **21**, 983–993 (2024). doi:10.1038/s41592-024-02260-3 Medline

64. K. Yusa, L. Zhou, M. A. Li, A. Bradley, N. L. Craig, A hyperactive piggyBac transposase for mammalian applications. *Proc. Natl. Acad. Sci. U.S.A.* **108**, 1531–1536 (2011). doi:10.1073/pnas.1008322108 Medline

65. R. Brosh, J. M. Laurent, R. Ordoñez, E. Huang, M. S. Hogan, A. M. Hitchcock, L. A. Mitchell, S. Pinglay, J. A. Cadley, R. D. Luther, D. M. Truong, J. D. Boeke, M. T. Maurano, A versatile platform for locus-scale genome rewriting and verification. *Proc. Natl. Acad. Sci. U.S.A.* **118**, e2023952118 (2021). doi:10.1073/pnas.2023952118 Medline

66. M. Martin, Cutadapt removes adapter sequences from high-throughput sequencing reads. *EMBnet. J.* **17**, 10–12 (2011). doi:10.14806/ej.17.1.200

67. H. Li, R. Durbin, Fast and accurate short read alignment with Burrows-Wheeler transform. *Bioinformatics* **25**, 1754–1760 (2009). doi:10.1093/bioinformatics/btp324 Medline

68. H. Li, B. Handsaker, A. Wysoker, T. Fennell, J. Ruan, N. Homer, G. Marth, G. Abecasis, R. Durbin; 1000 Genome Project Data Processing Subgroup, The Sequence Alignment/Map format and SAMtools. *Bioinformatics* **25**, 2078–2079 (2009). doi:10.1093/bioinformatics/btp352 Medline

69. S. Neph, M. S. Kuehn, A. P. Reynolds, E. Haugen, R. E. Thurman, A. K. Johnson, E. Rynes, M. T. Maurano, J. Vierstra, S. Thomas, R. Sandstrom, R. Humbert, J. A. Stamatoyannopoulos, BEDOPS: High-performance genomic feature operations. *Bioinformatics* **28**, 1919–1920 (2012). doi:10.1093/bioinformatics/bts277 Medline

70. A. R. Quinlan, I. M. Hall, BEDTools: A flexible suite of utilities for comparing genomic features. *Bioinformatics* **26**, 841–842 (2010). doi:10.1093/bioinformatics/btq033 Medline

71. J. T. Robinson, H. Thorvaldsdóttir, W. Winckler, M. Guttman, E. S. Lander, G. Getz, J. P. Mesirov, Integrative genomics viewer. *Nat. Biotechnol.* **29**, 24–26 (2011). doi:10.1038/nbt.1754 Medline

72. G. Yu, L.-G. Wang, Q.-Y. He, ChIPseeker: An R/Bioconductor package for ChIP peak annotation, comparison and visualization. *Bioinformatics* **31**, 2382–2383 (2015). doi:10.1093/bioinformatics/btv145 Medline

73. M. Hideto, M. Base, D. Bullock, bw2, morityun, snajder-r, ponnhide/pyCircos: pyCircos: Circos plot in matplotlib, Version v0.3.0, Zenodo (2022), https://zenodo.org/records/6477641.

74. Y. Hao, S. Hao, E. Andersen-Nissen, W. M. Mauck 3rd, S. Zheng, A. Butler, M. J. Lee, A. J. Wilk, C. Darby, M. Zager, P. Hoffman, M. Stoeckius, E. Papalexi, E. P. Mimitou, J. Jain, A. Srivastava, T. Stuart, L. M. Fleming, B. Yeung, A. J. Rogers, J. M. McElrath, C. A. Blish, R. Gottardo, P. Smibert, R. Satija, Integrated analysis of multimodal single-cell data. *Cell* **184**, 3573–3587.e29 (2021). doi:10.1016/j.cell.2021.04.048 Medline

75. S. L. Wolock, R. Lopez, A. M. Klein, Scrublet: Computational Identification of Cell Doublets in Single-Cell Transcriptomic Data. *Cell Syst.* **8**, 281–291.e9 (2019). doi:10.1016/j.cels.2018.11.005 Medline

76. Y. Wang, S. Xie, D. Armendariz, G. C. Hon, Computational identification of clonal cells in single-cell CRISPR screens. *BMC Genomics* **23**, 135 (2022). doi:10.1186/s12864-022-08359-1 Medline

77. A. M. Ribeiro-Dos-Santos, M. S. Hogan, R. D. Luther, R. Brosh, M. T. Maurano, Genomic context sensitivity of insulator function. *Genome Res.* **32**, 425–436 (2022). doi:10.1101/gr.276449.121 Medline

78. F. A. Wolf, P. Angerer, F. J. Theis, SCANPY: Large-scale single-cell gene expression data analysis. *Genome Biol.* **19**, 15 (2018). doi:10.1186/s13059-017-1382-0 Medline

79. A. Frankish, M. Diekhans, I. Jungreis, J. Lagarde, J. E. Loveland, J. M. Mudge, C. Sisu, J. C. Wright, J. Armstrong, I. Barnes, A. Berry, A. Bignell, C. Boix, S. Carbonell Sala, F. Cunningham, T. Di Domenico, S. Donaldson, I. T. Fiddes, C. García Girón, J. M. Gonzalez, T. Grego, M. Hardy, T. Hourlier, K. L. Howe, T. Hunt, O. G. Izuogu, R. Johnson, F. J. Martin, L. Martínez, S. Mohanan, P. Muir, F. C. P. Navarro, A. Parker, B. Pei, F. Pozo, F. C. Riera, M. Ruffier, B. M. Schmitt, E. Stapleton, M.-M. Suner, I. Sycheva, B. Uszczynska-Ratajczak, M. Y. Wolf, J. Xu, Y. T. Yang, A. Yates, D. Zerbino, Y. Zhang, J. S. Choudhary, M. Gerstein, R. Guigó, T. J. P. Hubbard, M. Kellis, B. Paten, M. L. Tress, P. Flicek, GENCODE 2021. *Nucleic Acids Res.* **49**, D916–D923 (2021). doi:10.1093/nar/gkaa1087 Medline

80. S. Pinglay, J.-B. Lalanne, S. Kottapalli, Genome Shuffle Seq: Multiplex generation and single cell analysis of structural variants in mammalian genomes, Version v1, Zenodo (2024); https://zenodo.org/records/14207585.

81. S. Fishilevich, R. Nudel, N. Rappaport, R. Hadar, I. Plaschkes, T. Iny Stein, N. Rosen, A. Kohn, M. Twik, M. Safran, D. Lancet, D. Cohen, GeneHancer: Genome-wide integration of enhancers and target genes in GeneCards. *Database* **2017**, bax028 (2017). doi:10.1093/database/bax028 Medline

82. Y. Luo, B. C. Hitz, I. Gabdank, J. A. Hilton, M. S. Kagda, B. Lam, Z. Myers, P. Sud, J. Jou, K. Lin, U. K. Baymuradov, K. Graham, C. Litton, S. R. Miyasato, J. S. Strattan, O. Jolanki, J.-W. Lee, F. Y. Tanaka, P. Adenekan, E. O'Neill, J. M. Cherry, New developments on the Encyclopedia of DNA Elements (ENCODE) data portal. *Nucleic Acids Res.* **48**, D882–D889 (2020). doi:10.1093/nar/gkz1062 Medline
